# De Novo Generation-Based Design of Potential Computational Hits Targeting the GluN1-GluN2A Receptor

**DOI:** 10.3390/molecules31030522

**Published:** 2026-02-02

**Authors:** Yibo Liu, Zhijiang Yang, Yixuan Guo, Tengxin Huang, Li Pan, Junjie Ding, Weifu Dong

**Affiliations:** 1 School of Chemical and Material Engineering, Jiangnan University, Wuxi 214000, China; 6230606077@stu.jiangnan.edu.cn; 2 State Key Laboratory of Chemistry for NBC Hazards Protection, Beijing 102205, China; yzjkid9@gmail.com (Z.Y.); yixuan020214@163.com (Y.G.); h1064482785@163.com (T.H.)

**Keywords:** GluN1-GluN2A receptor antagonists, de novo molecular generation, virtual screening, mechanism of action simulation, organic synthesis, electrophysiology

## Abstract

Central nervous system (CNS) disorders such as depression severely impair human health. Targeted inhibition of the GluN1-GluN2A receptor is a promising therapeutic strategy, but current drugs often have adverse effects. To develop novel candidate drugs, this study utilized the (S)-ketamine and GluN1-GluN2A receptor complex as a structural template and conducted de novo drug design with the DrugFlow platform. An integrated strategy of molecular docking-based virtual screening combined with high-throughput binding free energy (∆G_binding_) calculations from large-scale molecular dynamics (MD) simulations identified three promising antagonists. The ∆G_binding_ values of these compounds are all below −18.98 kcal/mol, indicating stronger binding affinity than (S)-ketamine, and they demonstrate promising drug-like properties and development potential. 200-ns MD simulations confirmed their stable receptor binding and mechanism consistent with (S)-ketamine. Electrophysiological recordings revealed that, at a concentration of 10 μM, Compounds **A1**, **A2**, and **A3** produced concentration-dependent inhibition of GluN1-GluN2A receptor-mediated currents, with fractional inhibition values of 24.26%, 35.36%, and 41.76%, respectively. These findings demonstrate the compounds’ potential as CNS disorder therapeutics, requiring further experiments to validate efficacy and advance development for conditions like depression.

## 1. Introduction

Neurological disorders such as epilepsy [1] and depression [2] are frequently accompanied by debilitating complications, including persecutory delusions [3] and memory dysfunction [4], and have become a significant burden on global health [5]. The N-methyl-D-aspartate (NMDA) receptor is a promising therapeutic target for these conditions [6], as selective inhibition of its excessive activation [7] and modulation of its signaling pathway to regulate mitochondrial autophagy [8] represent effective therapeutic strategies.

The NMDA receptor is an ion channel that mediates excitatory neurotransmission and consists of four principal domains: the amino-terminal domain (ATD), the ligand-binding domain (LBD), the transmembrane domain (TMD), and the carboxyl-terminal domain (CTD) [9]. It is assembled from GluN1 subunits in combination with GluN2 (A–D) or GluN3 (A–B) subunits, with distinct subunit compositions conferring unique pharmacological and electrophysiological properties. Functional NMDA receptors typically consist of two GluN1 and two GluN2 subunits [10]. The GluN1-GluN2A receptor subtype plays a pivotal role in synaptic transmission, synaptic plasticity, learning and memory, and nervous system development [11,12] via voltage- and ligand-dependent mechanisms [13], and is critically involved in the pathophysiology and regulation of central nervous system disorders [14]. Under pathological conditions, excessive secretion of homocysteine [15] selectively overactivates the GluN1-GluN2A receptor, triggering a sustained increase in intracellular Ca^2+^ levels. This cascade reaction will induce the continuous phosphorylation of extracellular signal-regulated kinase (ERK) and mitogen-activated protein kinase (MAPK), ultimately leading to neuronal cell death. This process is closely related to central nervous system diseases such as depression, epilepsy and post-traumatic stress disorder [16,17].

Ketamine and other ion channel blockers modulate this pathological cascade by inhibiting GluN1-GluN2A overactivation [18,19,20]. Ketamine is a racemic mixture composed of (R)-ketamine (arketamine) and (S)-ketamine (esketamine). (S)-ketamine was the first antidepressant to receive FDA breakthrough therapy designation [21], yet it is associated with adverse effects such as neuropsychiatric disturbances, hepatobiliary and urinary toxicity, and encephalatrophy [22]. In contrast, (R)-ketamine demonstrates stronger and more sustained antidepressant efficacy with fewer psychotropic side effects [23,24], undergoes hepatic cytochrome P450-mediated N-demethylation to form (R)-norketamine [25], and has been developed into an injectable formulation by Jiangsu Nhwa Pharmaceutical Co., Ltd. (Xuzhou, China) [26]. Furthermore, ketamine’s metabolite (2R,6R)-hydroxynorketamine (HNK) [27] and its five-membered ring analogue Ketamir-2 [28] are key candidates in NMDA receptor-targeted drug discovery. However, these compounds derived from (S)-ketamine share structural similarities and may have similar potential side effects to (S)-ketamine, highlighting the necessity of obtaining structurally diverse alternatives through de novo molecular generation [29]. To overcome the high costs and prolonged timelines of traditional drug development [30], computer-aided approaches offer accelerated pipelines [31,32], prompting this study to adopt ligand-receptor interaction-guided de novo design.

The work by Zhang et al. [33] provides critical theoretical support. Their findings show that (S)-ketamine blocks NMDA receptor overactivation by forming hydrogen bonds with GluN1-Asn616 and hydrophobic interactions with GluN2A-Leu642 and GluN1-Val644, thereby reversing stress-induced dendritic spine loss and neural circuit dysfunction [34,35], which underlies its therapeutic effect. The ResGen model developed by Zhang et al. [36] enables de novo molecule design within a defined protein binding pocket, offering technical feasibility for generating novel ligands based on receptor structural information. In this study, based on the complex of (S)-ketamine with GluN1-GluN2A receptor as a structural template, three potential antagonists were identified through de novo molecular generation, virtual screening, and high-throughput molecular mechanics-Poisson-Boltzmann surface area (MM-PBSA) binding free energy calculations (https://github.com/kotori-y/gmx_batch (accessed on 3 October 2024)). 200-ns molecular dynamics (MD) simulations confirmed that these compounds act as NMDA receptor ion channel blockers, functionally analogous to (S)-ketamine, and form structurally stable complexes with the GluN1-GluN2A receptor. As determined by ECFP4 fingerprints [37] fingerprint analysis, the three potential antagonists show a Tanimoto similarity of less than 0.50 with (S)-ketamine and other known active compounds, suggesting a distinct structural profile that may help circumvent the adverse effects commonly associated with (S)-ketamine [38].

To evaluate their in vitro activity, the selected compounds were synthesized via organic synthesis and tested for inhibitory effects on GluN1-GluN2A receptor currents at two concentrations using manual patch-clamp electrophysiology. The preliminary results indicate that these molecules exert inhibitory effects on the GluN1-GluN2A receptor. Nevertheless, further experimental validation is required to confirm both the potency of these antagonists and their in vivo efficacy.

## 2. Results

### 2.1. Results of Molecular Generation and Virtual Screening

**Figure 1:**
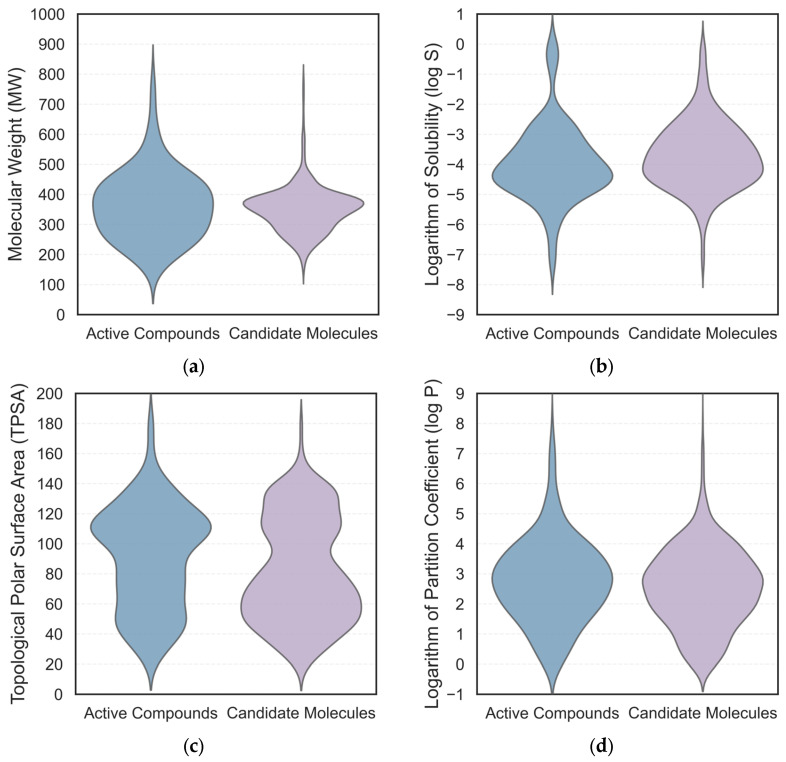
Comparative analysis of the physicochemical properties between active compounds and candidate molecules. (**a**) Comparative analysis of molecular weight (MW) distributions between active compounds and candidate molecules. (**b**) Comparative analysis of logarithm of water solubility (log S) distributions between active compounds and candidate molecules. (**c**) Comparative analysis of topological polar surface area (TPSA) distributions between active compounds and candidate molecules. (**d**) Comparative analysis of the octanol-water partition coefficient (log P) distributions between active compounds and candidate molecules. Blue: Active Compounds. Purple: Candidate Molecules.

In this study, 100,000 initial molecules were generated through de novo design. The structural similarity of these molecules to known active compounds was evaluated using ECFP4 fingerprints [37], and molecules with a Tanimoto similarity below 0.5 were retained. Then, these molecules were further screened based on four key physicochemical criteria. The screening conditions were a molecular weight (MW) of 200 to 400, a logarithm of a logarithm of water solubility (log S) of −6 to −1, a topological polar surface area (TPSA) of 20 to 140, and the octanol-water partition coefficient (log P) of 0 to 5. Ultimately, 20,000 candidate molecules were obtained, whose physicochemical properties were highly similar to those of the highly active antagonists, as shown in Figure 1. Therefore, these 20,000 molecules that met all four strict physicochemical criteria were selected for subsequent virtual screening.

The study utilized the KarmaDock [39] molecular docking tool to conduct an initial docking analysis of 20,000 molecules, aiming to investigate the interaction patterns between ligands and proteins [40]. The screening criteria required that molecules form hydrogen bond interactions with GluN1-Asn616 and exhibit hydrophobic interactions with GluN1-Val644 and GluN2A-Leu642. A second round of molecular docking was performed using CasriDock [41], and the results were re-scored with RTMScore [42] to evaluate the binding affinity of the candidate molecules toward the GluN1-GluN2A receptor. Based on the outcomes of the two docking analyses, 10,000 molecules were selected. To further screen for potential active compounds, the Glide XP docking method in Maestro V.15.2025 [43] was utilized for molecular docking, and SAscore (https://github.com/GeauxEric/SAscore (accessed on 23 September 2025)) [44] together with MultiSAScore V.202310 were employed to evaluate the synthetic accessibility of the compounds. A comprehensive screening was performed on 10,000 molecules.

Since the molecules to be screened in this study have relatively low structural similarity to (S)-ketamine, to ensure the biological relevance of the docking results, the molecular docking box was determined based on the key binding residues GluN2A-Leu642, GluN1-Asn616, and GluN1-Val644. According to Zhang et al. [33], the binding of (S)-ketamine to the GluN1-GluN2A receptor is a dynamic process: in the upper region of the ion channel, it is stabilized by hydrophobic interactions with GluN2A-Leu642, whereas in the lower region, hydrogen bonding with GluN1-Asn616 helps maintain its pharmacologically active conformation. To better recapitulate this dual-mode binding mechanism, after establishing baseline docking parameters, an additional hydrogen bond constraint targeting GluN1-Asn616 was incorporated into the docking protocol to more accurately simulate the binding mode.

**Figure 2:**
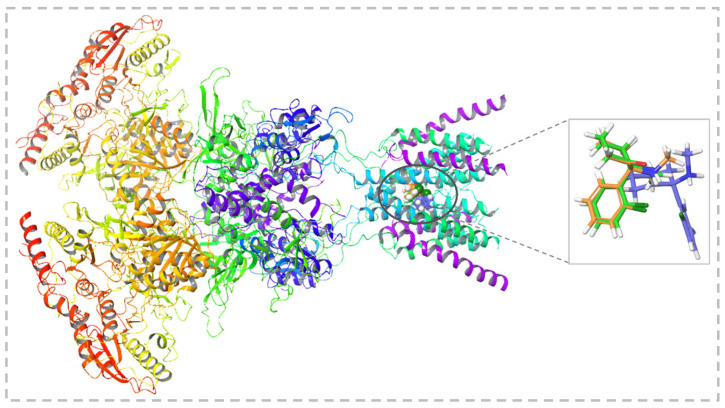
Analysis of the molecular docking result of the standard compound (S)-ketamine. Green: Ligand retrieved from the PDB database. Orange: Ligand resulting from molecular docking. Purple: Ligand derived from hydrogen bond-constrained molecular docking.

To verify the reliability of the Glide XP docking method, (S)-ketamine was used to validate the set docking parameters. As shown in Figure 2, under the condition without hydrogen bond constraints, the conformation of (S)-ketamine obtained from docking was highly consistent with the crystal structure, with no significant spatial displacement, and the RMSD value was 0.74 Å; while after introducing hydrogen bond constraints, the RMSD increased to 4.80 Å, indicating that the molecular conformation was directionally adjusted to meet the requirements of hydrogen bond formation, which is consistent with the dynamic binding model proposed by Zhang et al.

**Figure 3:**
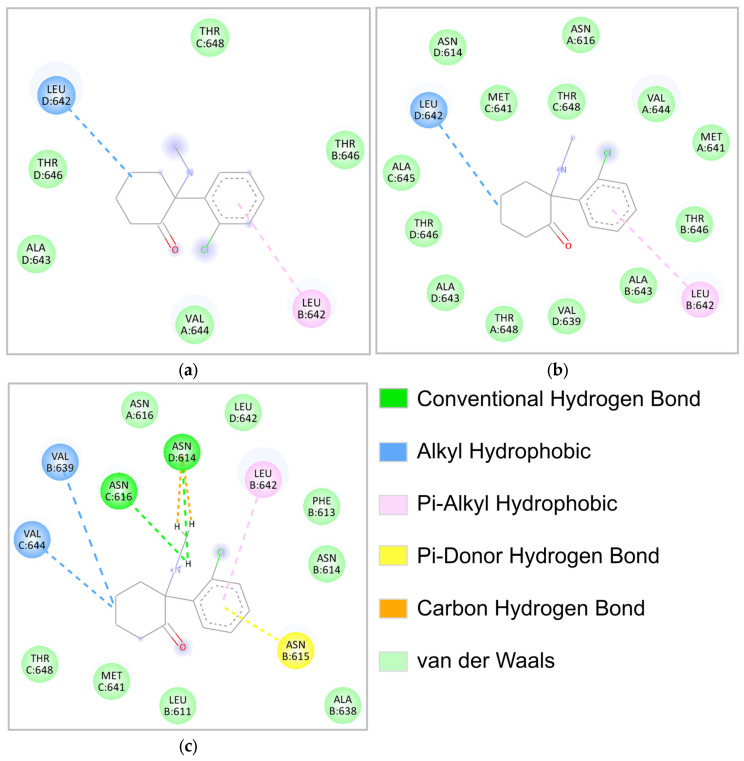
Comparison of the interaction between (S)-ketamine and the GluN1-GluN2A receptor prior to and following molecular docking. (**a**): Interaction between (S)-ketamine and the GluN1-GluN2A receptor prior to molecular docking. (**b**): Interaction between (S)-ketamine and the GluN1-GluN2A receptor following molecular docking. (**c**): Interaction of (S)-ketamine with the GluN1-GluN2A receptor following hydrogen bond-constrained molecular docking.

As shown in Figure 3, the molecular interactions after molecular docking were consistent with existing studies. In the GluN1-GluN2A receptor, chain A and chain C correspond to the GluN1 subunit, and chain B and chain D correspond to the GluN2A subunit. The original (S)-ketamine had alkyl interactions with Leu642 of chain D and π-alkyl interactions with Leu642 of chain B; in the unconstrained docking, these hydrophobic contacts were retained. In the hydrogen bond constrained docking, (S)-ketamine not only formed conventional hydrogen bonds with GluN1-Asn616 and GluN2A-Asn614, but also formed a carbon-hydrogen bond with GluN2A-Asn614 and a π-donor hydrogen bond with GluN2A-Asn615. In addition, it still maintained hydrophobic contacts with Leu642 through alkyl and π-alkyl interactions, and formed alkyl interactions with Val639 and Val644.

The above results validate the accuracy and rationality of the two molecular docking strategies adopted in this study. Given that the aim of this research is to screen out new candidate molecules with a similar mechanism of action to (S)-ketamine and with a lower similarity in molecular scaffold structure, if only the consistency of spatial position and interaction with the original (S)-ketamine after molecular docking is pursued, it will lead to experimental artifacts. Therefore, the docking protocol including the GluN1-Asn616 hydrogen bond constraint was ultimately selected as the standard process for subsequent virtual screening in this study. The aim is to first filter out molecules that cannot form a hydrogen bond with GluN1-Asn616 and cannot form a hydrophobic interaction with GluN2A-Leu642 through Glide XP molecular docking, and then select molecules with a docking score better than (S)-ketamine based on the docking score.

**Figure 4:**
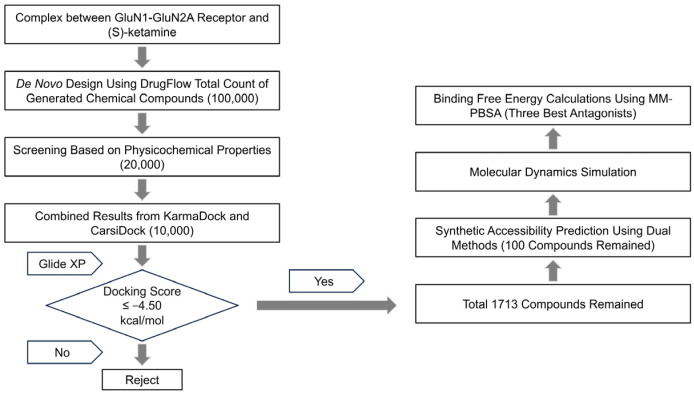
Comparative analysis of the physicochemical properties between active compounds and candidate molecules.

Approximately 3000 molecules that met the docking conditions were obtained through Glide XP molecular docking with hydrogen bond constraints. Then, by screening for molecules with docking scores lower than −4.50 kcal/mol of (S)-ketamine, about 2000 molecules were selected. The synthetic accessibility of these 2000 molecules was evaluated using SAscore [44] and MultiSAScore V.202310. As shown in Figure 4, through repeated screening processes, approximately 100 molecules that met the criteria were obtained. These molecules were first subjected to preliminary molecular dynamics simulations and then binding free energy calculations.

### 2.2. ∆G_binding_ Calculations Using the MM-PBSA Approach

This study utilized the MM-PBSA approach to calculate the binding free energy (∆G_binding_) and assess the binding affinity of candidate molecules for the GluN1-GluN2A receptor, thus identifying potential novel antagonists. The MM-PBSA method is more accurate than static scoring functions such as molecular docking. The MM-PBSA method can analyze the contribution of individual residues or specific energy terms through free energy decomposition, revealing the energy characteristics at the residue level and identifying key interactions in the molecular binding process, thereby providing a theoretical basis for personalized drug design [45]. The lower the ∆G_binding_ of a molecule, the stronger its binding affinity for the GluN1-GluN2A receptor. To enhance the reliability of the binding free energy values obtained from the MM-PBSA method, this study performed three independent replicate simulations, and the final result was derived by averaging the calculated values across these replicates.

The results of the ∆G_binding_ calculations are presented in Supplementary Materials ESM_T1. It can be seen from the results that the ∆G_binding_ of over 40% of the molecules is lower than that of (S)-ketamine at −18.98 kcal/mol, demonstrating superior binding affinity to the GluN1-GluN2A receptor and promising inhibitory potential. Furthermore, this study performed residue-wise binding free energy decomposition to identify key amino acid residues in the GluN1-GluN2A receptor that make significant contributions to the overall binding affinity. This analysis enables a preliminary assessment of whether the candidate molecules engage in interaction patterns with the receptor comparable to those of (S)-ketamine, particularly in terms of critical residue-level interactions. In this study, the root mean square deviation (RMSD) of the compound–receptor complex system was calculated throughout the molecular dynamics simulation to assess the structural stability of the complex. To further identify promising novel antagonists of the GluN1-GluN2A receptor, this study evaluated the drug-likeness and pharmacokinetic properties of the aforementioned molecules.

### 2.3. Pharmacokinetic and Drug-likeness Evaluation

Drugs are a vaguely defined class of chemical entities, which, other than displaying affinity to the intended therapeutic target, must also fulfill certain criteria viz., bioavailability and efficacy [46]. The majority of drug candidates failed during the development process due to unfavorable pharmacokinetic (PK) properties. Therefore, ADMET properties are considered key determinants in the screening of ligands, based on established drug-likeness criteria such as Lipinski’s rule of five, Ghose’s filter, and Veber’s rules [47]. In this study, the absorption (A), distribution (D), metabolism (M), excretion (E) properties, and drug-likeness of the selected molecules were evaluated using the ADMETlab 3.0 platform [48], to further assess their therapeutic potential. This study comprehensively evaluated the quantitative estimate of drug-likeness (QED), human intestinal absorption (HIA) potential, blood-brain barrier (BBB) permeability, and plasma clearance (CL_plasma_) of the candidate antagonists. The drug-likeness and pharmacokinetic profiles of the candidate antagonists are summarized in Supplementary Materials ESM_T1.

**Figure 5:**
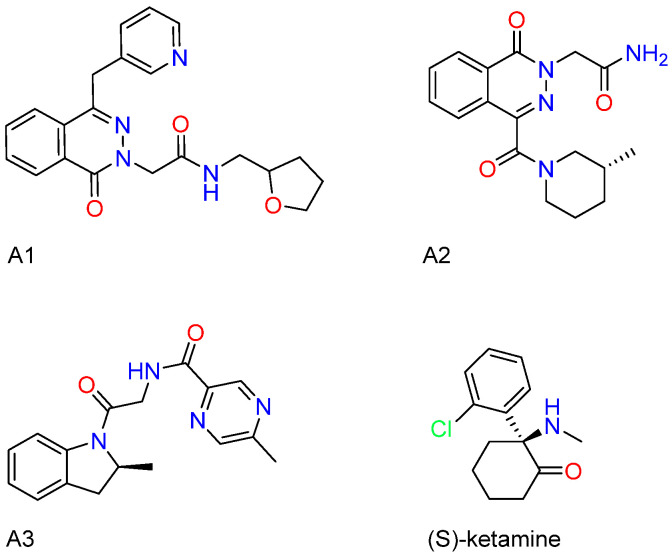
Two-dimensional structural diagrams of compounds **A1**, **A2**, **A3**, and (S)-ketamine.

This study screened out three potential antagonists for further research through a comprehensive analysis of the binding free energy results of each molecule calculated by the MM-PBSA method, the contribution of key amino acid residues when calculating the binding free energy of each molecule with the receptor by the MM-PBSA method, the stability of the ligand and GluN1-GluN2A receptor complex system during the dynamic simulation process, the drug-likeness and pharmacokinetic calculation evaluation results of the candidate molecules, and the expert’s assessment of the synthetic difficulty of these compounds. The two-dimensional structures of these molecules are shown in Figure 5. The standard antagonist (S)-ketamine was selected as the experimental control throughout the entire research process.

The results of ∆G_binding_ and four energy terms for the three selected molecules and (S)-ketamine are shown in Table 1. As shown in Table 1, the binding free energies of the three selected molecules are −26.33 kJ/mol, −21.49 kJ/mol, and −20.70 kJ/mol, respectively, all of which are lower than that of (S)-ketamine. Therefore, all the selected novel molecules demonstrate higher binding affinity toward the protein and show promising potential as inhibitors of the GluN1-GluN2A receptor.

As shown in Table 2, the QED values of the three molecules are all above 0.7, and their molecular weights (MW) fall within the range of 200–400 g/mol. The above results suggest that the selected molecules possess favorable drug-like properties and hold promising therapeutic potential. HIA^−^ represents the probability that the drug molecule lacks human intestinal absorption (HIA) capability, while BBB^+^ denotes the probability that the molecule exhibits good blood-brain barrier (BBB) penetration ability. As shown in Table 2, the three candidate antagonists exhibit favorable human intestinal absorption (HIA) and blood-brain barrier (BBB) permeability. The topological polar surface area (TPSA) values of the three candidate molecules are 86.11 Å^2^, 98.29 Å^2^, and 75.19 Å^2^ respectively, which are higher than the TPSA threshold recommended by the pharmacopoeia for CSN drugs. However, based on the comprehensive assessment results from the ADMETlab 3.0 platform, these molecules still show good potential for blood-brain barrier (BBB) penetration. Given that BBB permeability is influenced by multiple factors, a single parameter like TPSA is not sufficient to rule out the possibility of their central nervous system (CNS) activity. Therefore, this study believes that the selected molecules have a certain ability to penetrate the blood-brain barrier and have the potential to be developed as candidate drugs for the treatment of CNS diseases. CL_plasma_ represents the sum of drug clearance rates in organs such as the liver and kidneys, reflecting the body’s capacity to eliminate drugs, with units expressed in mL/min/kg. As shown in Table 2, these three potential antagonists display moderate plasma clearance (CL_plasma_), effectively balancing efficient drug elimination with sustained pharmacological activity. Collectively, compounds **A1**, **A2**, and **A3** exhibit strong promise as candidates for therapeutic development.

### 2.4. Molecular Interaction Analysis

**Figure 6:**
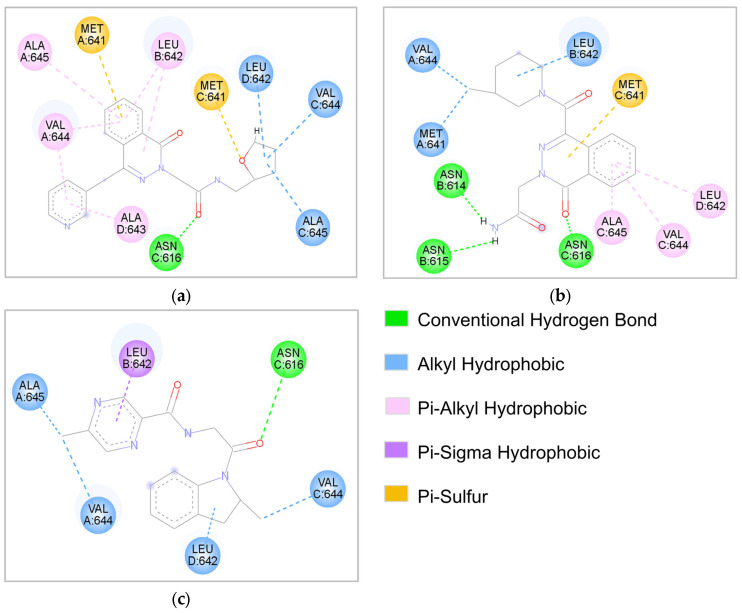
Binding interactions between potential antagonists and the GluN1-GluN2A receptor. (**a**): Interaction mode of **A1** with the GluN1-GluN2A receptor. (**b**): Interaction mode of **A2** with the GluN1-GluN2A receptor. (**c**): Interaction mode of **A3** with the GluN1-GluN2A receptor.

To further confirm the research value of these molecules, Figure 6 presents the specific and detailed interaction modes of the selected molecules with the GluN1-GluN2A receptor during the Glide XP molecular docking process. Compound **A1** forms a stable docking complex with the GluN1-GluN2A receptor through hydrogen bonds and hydrophobic interactions. Compound **A1** forms a hydrogen bond interaction with the amino acid residue Asn616, a π-sulfur interaction with Met641 in the A chain, and a sulfur-oxygen interaction with Met641 in the C chain. In addition to these key interactions, **A1** establishes hydrophobic contacts with Leu642 in the B chain, Val644 and Ala645 in the A chain, and Ala643 in the D chain via π-alkyl interactions; it also forms hydrophobic contacts with Val644 and Ala645 in the C chain and Leu642 in the D chain through alkyl interactions, thereby stabilizing the overall conformation of the complex. In the docking analysis, the binding affinity between the GluN1-GluN2A receptor and compound **A1** was determined to be −6.78 kcal/mol. Molecular interaction analysis of **A2** with the GluN1-GluN2A receptor revealed that **A2** forms hydrogen bond interactions with GluN1-Asn616, GluN2A-Asn614, and GluN2A-Asn615, as well as a π-sulfur interaction with Met641 in the C chain. Additionally, **A2** establishes hydrophobic contacts with Met641 and Val644 in the A chain and Leu642 in the B chain via alkyl interactions, and with Ala645 and Leu642 in the D chain and Val644 in the C chain through π-alkyl interactions. Docking results indicate that the binding affinities of **A2** and **A3** are −6.35 kcal/mol and −5.70 kcal/mol, respectively. Compound **A3** forms a hydrogen bond interaction with Asn616 and a hydrophobic contact with Leu642 in the B chain via a π-sigma interaction. Furthermore, **A3** engages in hydrophobic contacts with Ala645, Val644, and Leu642 in the D chain through alkyl interactions, contributing to complex stabilization.

These three potential antagonists exhibit lower docking scores of −6.78, −6.35, and −5.70 kcal/mol, respectively, and more favorable molecular mechanics–Poisson–Boltzmann–surface area (MM-PBSA) binding free energies of −26.33, −21.49, and −20.70 kcal/mol, respectively, compared with (S)-ketamine (docking score: −4.50 kcal/mol; MM-PBSA Δ_Gbinding_: −18.98 kcal/mol). This collectively indicates stronger binding affinity and enhanced capacity for stable, functionally relevant receptor engagement. In summary, molecular interaction analysis between ligands and the GluN1-GluN2A receptor through molecular docking provides a structural basis for subsequent molecular dynamics simulations. To further investigate the dynamic behavior of the designed compounds as GluN1-GluN2A receptor antagonists, the aforementioned docking complexes were subjected to 200 ns all-atom molecular dynamics simulations.

### 2.5. Binding Stability Analysis Based on Post-MD Simulations

**Figure 7:**
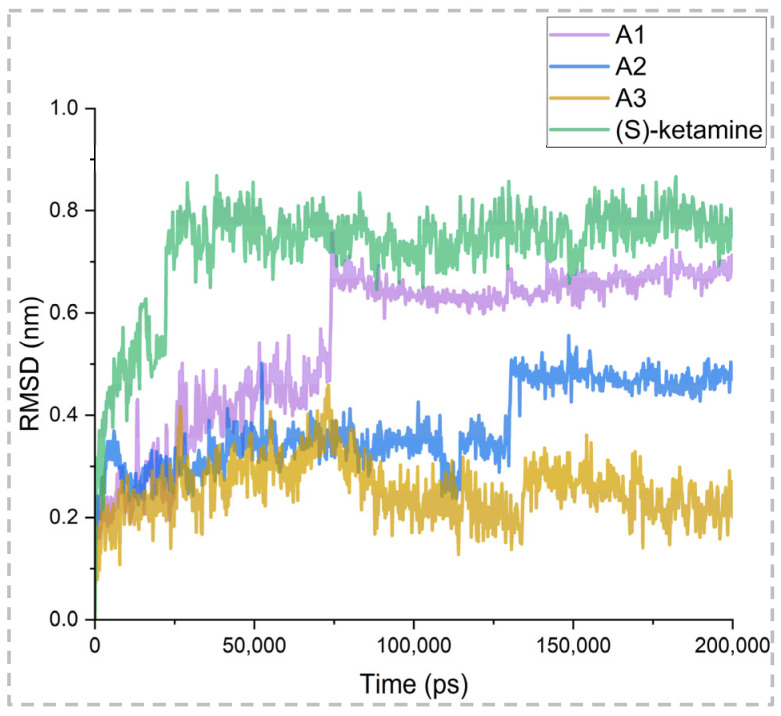
Backbone RMSD of GluN1-GluN2A receptor bound with **A1**, **A2**, **A3**, and (S)-ketamine.

Molecular dynamics simulations can not only resolve spatial steric hindrance conflicts between proteins and ligands but also provide accurate insights into the binding modes and binding affinities of compounds [49]. To further explain the mechanism of action of the three selected molecules and observe their binding stability with ion channels under physiological conditions, this study calculated the root mean square deviation (RMSD) of the backbone, the root mean square fluctuation (RMSF) of amino acid residues, the contribution of key residues within a 4 Å range of the binding site to the binding free energy, the gyration radius (Rg) of the ligand molecules, and the gyration radius (Rg) of the complex system for the three potential antagonists and (S)-ketamine with the receptor complex. The root mean square deviation (RMSD) reflects the dynamic stability of ligand binding and protein conformational changes. All complex systems reached equilibrium within 200 ns of MD simulation. As shown in Figure 7, at approximately 70 ns, the backbone of the GluN1-GluN2A receptor bound to **A1** exhibited a deviation of up to 0.2 nm and eventually stabilized at around 0.7 nm. The GluN1-GluN2A receptors bound to the other compounds remained structurally stable throughout the simulation. As shown in Table 3, the differences between the maximum and average RMSD when bound to **A1**, **A2**, **A3**, and (S)-ketamine were 0.21, 0.18, 0.21, and 0.14 nm, respectively. No significant structural deviations were observed during the MD simulation, indicating that the system remained in a stable conformational state throughout.

**Figure 8:**
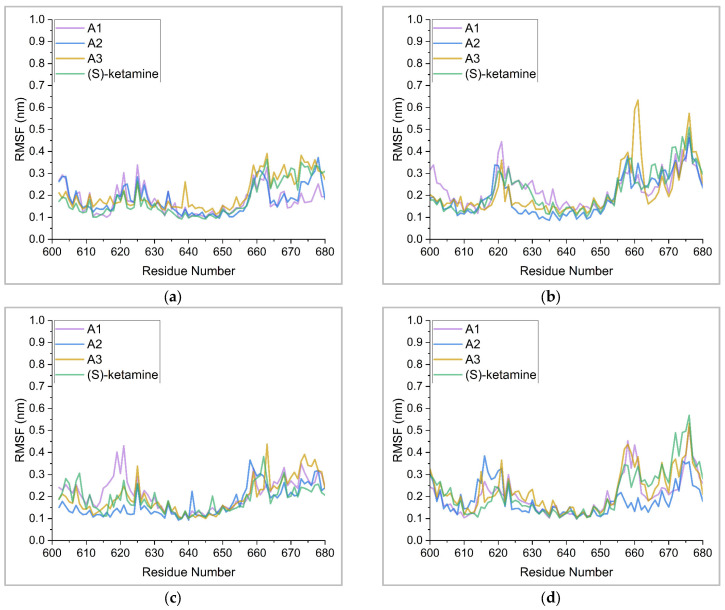
RMSF of amino acid residues in the GluN1-GluN2A receptor bind with A1, A2, A3, and (S)-ketamine. (**a**): RMSF of amino acid residues in the protein chain A bound with **A1**, **A2**, **A3**, and (S)-ketamine. (**b**): RMSF of amino acid residues in the protein chain B bound with **A1**, **A2**, **A3**, and (S)-ketamine. (**c**): RMSF of amino acid residues in the protein chain C bound with **A1**, **A2**, **A3**, and (S)-ketamine. (**d**): RMSF of amino acid residues in the protein chain D bound with **A1**, **A2**, **A3**, and (S)-ketamine.

Amino acids play a critical role in maintaining the structural stability of protein-ligand complexes. In this study, the root mean square fluctuations (RMSF) of amino acid residues in the GluN1-GluN2A receptor were calculated, and the results are presented in Figure 8. The RMSF values are summarized in Table 4. The maximum and minimum RMSF values of the A chain when bound to **A1**, **A2**, **A3**, and (S)-ketamine were 0.34 and 0.10, 0.37 and 0.10, 0.39 and 0.11, and 0.36 and 0.09 nanometers, respectively. The maximum and minimum RMSF values of the B chain when bound to **A1**, **A2**, **A3**, and (S)-ketamine were 0.48 and 0.12, 0.46 and 0.09, 0.63 and 0.12, and 0.51 and 0.10 nanometers, respectively. The maximum and minimum RMSF values of the C chain when bound to **A1**, **A2**, **A3**, and (S)-ketamine were 0.43 and 0.10, 0.37 and 0.09, 0.44 and 0.10, and 0.38 and 0.10 nanometers, respectively. The maximum and minimum RMSF values of the D chain when bound to **A1**, **A2**, **A3**, and (S)-ketamine were 0.51 and 0.10, 0.38 and 0.11, 0.53 and 0.10, and 0.57 and 0.10 nanometers, respectively. The relatively low RMSF values indicate that the amino acid residues in the GluN1-GluN2A receptor exhibit minimal conformational fluctuations following binding to the proposed molecules.

In addition, the contribution of amino acid residues to the binding free energy provides a valuable metric for identifying key residues involved in ligand binding. In this study, the energetic contributions of amino acid pairs within a 4 Å distance from the binding site to the binding free energy of the selected molecules were calculated, and the results are presented in Supplementary Materials ESM_T2–ESM_T5. Notably, residues Asn616, Leu642, and Val644 consistently exhibited high energetic contributions, indicating their critical role in stabilizing ligand binding. Thus, these residues are likely to be the primary interactors with the potential antagonist molecules. Furthermore, most amino acid residues in proximity to the binding site displayed positive energy contributions, with only a few exceptions.

**Figure 9:**
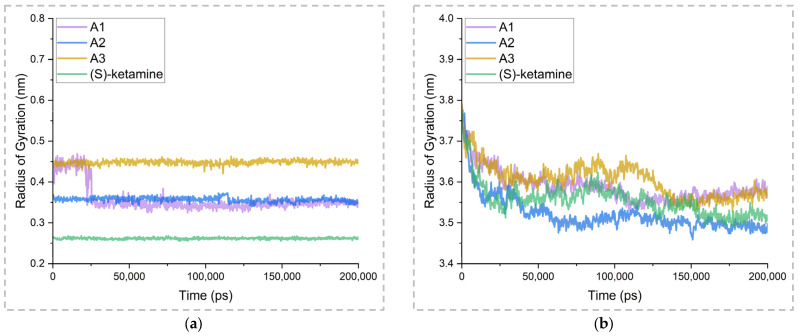
Radius of gyration of the proposed inhibitors and their corresponding complexes. (**a**): Radius of gyration of the proposed inhibitors; (**b**): Radius of gyration of GluN1-GluN2A receptor bound with proposed inhibitors.

The radius of gyration (Rg) of the ligand during the MD simulation serves as an indicator of ligand conformational stability. The Rg values for the selected molecules and (S)-ketamine were calculated. As shown in Figure 9a, all molecules maintained conformational stability throughout the MD simulation. To further assess the conformational stability of each molecule, the difference between the maximum and average Rg values was calculated. As shown in Table 5, the differences between the maximum Rg value and the average Rg value for **A1**, **A2**, **A3**, and (S)-ketamine were 0.11 nm, 0.01 nm, 0.01 nm, and 0.01 nm, respectively. These results indicate that the selected molecules exhibited no significant conformational deviation during the simulation. The calculations of root mean square deviation (RMSD), root mean square fluctuation (RMSF), and radius of gyration (Rg) in this study indicate that the proposed molecules can stably reside within the ion channel of the GluN1-GluN2A receptor under dynamic conditions. The radius of gyration (Rg) of the complex system reflects the overall compactness of the ligand-protein complex and the trend of conformational changes during the MD simulation. As shown in Figure 9b, all systems maintained overall structural compactness throughout the MD simulation. To further assess the structural stability of each system, the difference between the maximum and average Rg values was calculated. As shown in Table 5, the differences between the maximum Rg value and the average Rg value for **A1**, **A2**, **A3**, and (S)-ketamine complexes with the protein were 0.20 nm, 0.26 nm, 0.18 nm, and 0.22 nm, respectively. These results indicate that the selected molecules can maintain normal structural compactness of the complex upon binding to the target and prevent excessive loosening. The calculations of root mean square deviation (RMSD), root mean square fluctuation (RMSF), and radius of gyration (Rg) in this study indicate that the proposed molecules stably reside within the ion channel of the GluN1-GluN2A receptor under dynamic conditions.

### 2.6. Compounds Synthesis and Characterization

#### 2.6.1. Synthesis and Characterization of Compound **A1**

**Scheme 1:**
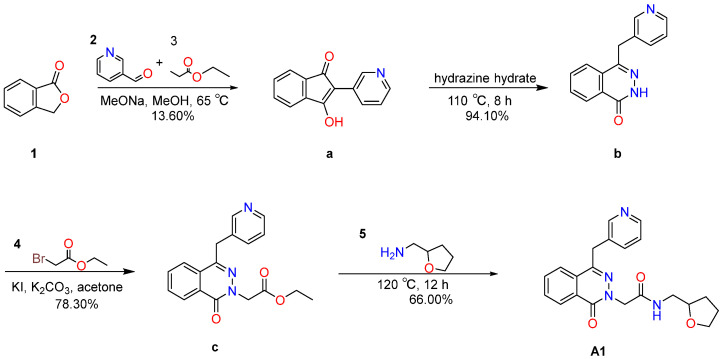
Synthesis flowchart of compound **A1**.

The synthesis process of compound **A1** is shown in Scheme 1. Starting from 200 mg of compound **1**, the intermediate compound a was obtained as a yellow solid (45 mg, yield 13.60%). It was identified by liquid chromatography-mass spectrometry (S195-1-B1), with a retention time (t_R_) of 0.960 min and an observed [M+H]^+^ ion mass of 224.10, which was in high agreement with the calculated value of C_14_H_9_NO_2_ (224.06). Comprehensive characterization by ^1^H NMR (400 MHz, DMSO-d_6_) revealed signals fully consistent with the proposed structure: δ 9.69–9.79 (m, 1H), 9.43–9.52 (m, 1H), 8.23 (d, *J* = 8.4 Hz, 1H), 7.81 (dd, *J* = 8.4, 1.6 Hz, 1H), 7.37–7.44 (m, 2H), 7.28–7.36 (m, 2H). Subsequent conversion of Compound **a** afforded intermediate Compound **b** as a yellow solid (45 mg, 94.10% yield). LC–MS analysis confirmed its molecular identity (t_R_ = 0.715 min; [M+H]^+^ at *m*/*z* 238.10), matching the calculated mass for C_14_H_11_N_3_O (238.09). ^1^H NMR (400 MHz, DMSO-d_6_) data further supported the assigned structure: δ 12.59 (s, 1H), 8.63 (dd, *J* = 4.8, 1.6 Hz, 1H), 8.42–8.45 (m, 1H), 8.27–8.30 (m, 1H), 8.03 (d, *J* = 8.4 Hz, 1H), 7.86–7.95 (m, 2H), 7.74–7.79 (m, 1H), 7.33–7.35 (m, 1H), 4.37 (s, 2H). Compound **b** was then transformed into intermediate Compound **c**, isolated as a yellow solid (48 mg, 78.30% yield). LC–MS (S195-3-A1) confirmed the formation of Compound **c** (t_R_ = 1.003 min; [M+H]^+^ at *m*/*z* 324.10), consistent with the theoretical mass of C_18_H_17_N_3_O_3_ (324.13). ^1^H NMR (400 MHz, DMSO-d_6_) analysis corroborated structural assignment: δ 8.63 (s, 1H), 8.44 (d, *J* = 8.4 Hz, 1H), 8.31 (d, *J* = 8.0 Hz, 1H), 8.04 (d, *J* = 8.4 Hz, 1H), 7.86–7.98 (m, 2H), 7.71 (d, *J* = 8.0 Hz, 1H), 7.33 (dd, *J* = 8.4, 1.6 Hz, 1H), 4.96 (s, 1H), 4.40 (s, 1H), 4.19 (q, *J* = 7.2 Hz, 2H), 2.52 (s, 4H), 1.23 (t, *J* = 7.2 Hz, 3H). Finally, Compound **c** was successfully elaborated to target Compound **A1**, isolated as a pale yellow solid (37 mg, 66.00% yield).

Comprehensive characterization data for Compound **A1** are summarized below. LC-MS analysis (S195-5-C3) showed a t_R_ of 0.853 min and an [M+H]^+^ ion at *m*/*z* 379.20, in excellent agreement with the calculated value for C_21_H_22_N_4_O_3_ (379.17). HPLC analysis confirmed high purity (t_R_ = 1.664 min; 99.27% area normalized). The ^1^H NMR spectrum (400 MHz, DMSO-d_6_, S195-5-C3; see ESM_F1) displayed characteristic resonances consistent with the target structure: δ 8.65–8.60 (m, 1H), 8.42 (d, *J* = 8.4 Hz, 1H), 8.32–8.27 (m, 1H), 8.22 (t, *J* = 7.6 Hz, 1H), 7.98 (s, 1H), 7.94–7.81 (m, 2H), 7.74–7.68 (m, 1H), 7.31 (d, *J* = 8.0 Hz, 1H), 4.77 (s, 2H), 4.36 (s, 2H), 3.86 (m, 1H), 3.78 (m, 1H), 3.63 (m, 1H), 3.18 (m, 2H), 1.85 (d, *J* = 6.8 Hz, 3H), 1.54 (s, 1H). ^13^C NMR (101 MHz, DMSO-d_6_) exhibited 21 distinct carbon signals at δ 167.43, 159.00, 150.21, 148.26, 144.72, 136.47, 134.11, 133.95, 132.28, 129.19, 128.10, 126.94, 126.03, 123.97, 77.54, 67.65, 53.68, 43.23, 35.17, 28.93, and 25.62 ppm; fully consistent with the expected carbon count and substitution pattern of C_21_H_22_N_4_O_3_. Collectively, orthogonal spectroscopic (^1^H NMR, ^13^C NMR) and chromatographic (LC-MS, HPLC) data unambiguously confirm the successful synthesis of Compound **A1** in high chemical purity and with complete structural fidelity. 

#### 2.6.2. Synthesis and Characterization of Compound **A2**

**Scheme 2:**
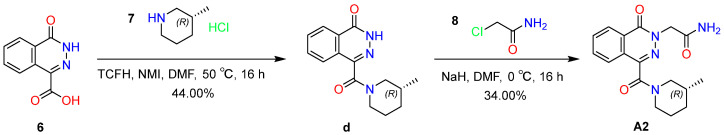
Synthesis flowchart of compound **A2**.

The synthesis of Compound **A2** is outlined in Scheme 2. Initially, (R)-4-(3-methylpiperidin-1-carbonyl)-1H-phthalazin-1-one (Compound **d**) was prepared via amide coupling between 4-oxo-3,4-dihydrophthalazine-1-carboxylic acid (Compound **6**) and (R)-3-methylpiperidine hydrochloride (Compound **7**), using TCFH as the coupling reagent and DIEA as the non-nucleophilic base in anhydrous DMF. Purification by preparative HPLC (mobile phase: 0.04% aqueous ammonium formate, acetonitrile/water = 30:70 *v*/*v*) afforded Compound **d** as a white solid (105 mg, 44% yield). Its structure was clearly confirmed by liquid chromatography-mass spectrometry (LC-MS), which showed a [M+H]^+^ ion at *m*/*z* 272.10, in excellent agreement with the calculated value of C_15_H_17_N_3_O_2_ (272.13). Comprehensive characterization data for Compound **d** are as follows: ^1^H NMR (400 MHz, DMSO-d_6_) δ 12.79 (br s, 1H), 8.30 (d, *J* = 8.0 Hz, 1H), 7.85–8.04 (m, 2H), 7.68 (d, *J* = 7.6 Hz, 1H), 4.39 (t, *J* = 7.2 Hz, 1H), 3.50 (d, *J* = 12.4 Hz, 1H), 2.87–3.07 (m, 1H), 2.60–2.76 (m, 1H), 1.62–1.85 (m, 2H), 1.44–1.60 (m, 1H), 1.13–1.38 (m, 2H), 0.68–1.01 (m, 3H); specific rotation [α]^23^D = −33.83 (c 1.00, MeOH); enantiomeric excess >99.9% ee (SFC, Chiralpak IC column; mobile phase: n-hexane/i-PrOH = 80:20 *v*/*v*; flow rate: 1.0 mL/min; temperature: 25 °C). Compound **A2** was subsequently synthesized via regioselective O-alkylation of Compound **d** with 2-chloroacetamide (b8), followed by spontaneous intramolecular cyclization under strictly anhydrous conditions and controlled basicity (NaH, 1.2 equiv, anhydrous DMF, 0 °C → rt, 16 h).

The reaction mixture was carefully quenched with saturated aqueous NH_4_Cl solution to ensure complete and safe decomposition of residual NaH. After standard aqueous workup, the crude product was purified by preparative HPLC (mobile phase: 0.04% aqueous ammonium formate in acetonitrile/water = 30:70 *v*/*v*), affording Compound **A2** as a white solid (25 mg, 34% yield). High-resolution LC–MS analysis unambiguously confirmed the molecular formula: [M+H]^+^ at *m*/*z* 329.30 (calculated for C_17_H_20_N_4_O_3_: 329.15). Structural assignment was rigorously supported by comprehensive NMR characterization: ^1^H NMR (400 MHz, DMSO-d_6_; see ESM_F2) δ 8.32–8.28 (m, 1H), 7.98–7.87 (m, 2H), 7.74–7.68 (m, 1H), 7.58 (s, 1H), 7.21 (d, *J* = 7.5 Hz, 1H), 4.79–4.57 (m, 2H), 4.43–4.29 (m, 1H), 3.64–3.57 (m, 1H), 3.02–2.85 (m, 1H), 2.70–2.56 (m, 1H), 1.81–1.61 (m, 2H), 1.55–1.44 (m, 1.5H), 1.32–1.12 (m, 1.5H), 0.94 (d, *J* = 6.6 Hz, 1.5H), 0.70 (d, *J* = 6.6 Hz, 1.5H); ^13^C NMR (101 MHz, DMSO-d_6_) δ 169.05, 162.40, 158.81, 141.67–142.22 (br, 1C), 134.32, 132.89, 127.54–128.28 (br, 1C), 126.84, 125.98, 53.45–53.99 (br, 1C), 42.21, 32.80, 32.01, 31.28, 25.00, 19.37, 18.75; specific rotation [α]^23^D = −19.47 (c 1.00, MeOH); enantiomeric excess >99.9% ee (SFC, Chiralpak IC column; n-hexane/i-PrOH = 80:20 *v*/*v*; 1.0 mL/min; 25 °C).

#### 2.6.3. Synthesis and Characterization of Compound **A3**

**Scheme 3:**
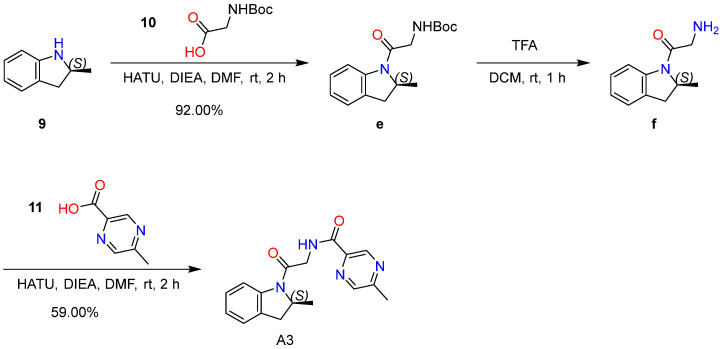
Synthesis flowchart of compound **A3**.

The synthesis of Compound **A3** is outlined in Scheme 3. At room temperature, (S)-tert-butyl (2-(2-methylindolin-1-yl)-2-oxoethyl)carbamate (Compound **e**) was synthesized via amide coupling between (S)-2-methylindoline (Compound **9**) and 2-((tert-butoxycarbonyl)amino)acetic acid (Compound **10**), using HATU as the coupling reagent and DIEA as the non-nucleophilic base in anhydrous DMF. The reaction mixture was quenched with ice-cold water, extracted with ethyl acetate (EtOAc), washed sequentially with saturated aqueous NaHCO_3_ and brine, dried over anhydrous Na_2_SO_4_, filtered, and concentrated under reduced pressure. The crude residue was purified by flash column chromatography (petroleum ether/EtOAc = 3:1, *v*/*v*), affording Compound **e** as a yellow oil (201 mg, 92.00% yield). Analytical TLC (petroleum ether/EtOAc = 3:1) confirmed complete consumption of Compound **9** (Rf = 0.1), with Compound **e** exhibiting Rf = 0.5. Structural identity was unambiguously confirmed by LC–MS, which displayed a protonated molecular ion at *m*/*z* 291.10 ([M+H]^+^), in excellent agreement with the calculated value for C_16_H_22_N_2_O_3_ (291.16). ^1^H NMR (400 MHz, CDCl_3_) δ 8.11 (d, *J* = 8.0 Hz, 1H), 7.23 (d, *J* = 8.0 Hz, 2H), 7.09–7.07 (m, 1H), 5.56 (br s, 1H), 4.48 (br s, 1H), 4.08–4.29 (m, 2H), 3.46 (dd, *J* = 12.4, 4.0 Hz, 1H), 2.71 (d, *J* = 4.8 Hz, 1H), 1.47 (s, 9H), 1.25–1.33 (m, 3H).

Deprotection of Compound **e** was performed by treating with trifluoroacetic acid (TFA) in dichloromethane at room temperature, affording (S)-2-amino-1-(2-methylindolin-1-yl)ethenone (Compound **f**) as a reactive intermediate. The reaction mixture was concentrated under reduced pressure, and the crude residue was carried forward directly into the next step without purification. LC–MS analysis confirmed the formation of Compound **f**: [M+H]^+^ observed at *m*/*z* 191.10 (calculated for C_11_H_14_N_2_O: 191.11). Structural assignment was corroborated by comprehensive characterization: ^1^H NMR (400 MHz, DMSO-d_6_) δ 8.02 (br s, 1H), 7.97 (d, *J* = 8.0 Hz, 1H), 7.25 (t, *J* = 7.6 Hz, 1H), 7.01 (t, *J* = 7.6 Hz, 1H), 4.67 (br s, 1H), 3.60 (d, *J* = 12.4 Hz, 1H), 3.43 (d, *J* = 12.4 Hz, 1H), 3.35 (dd, *J* = 12.4, 4.0 Hz, 1H), 2.64 (d, *J* = 4.8 Hz, 1H), 1.74 (br s, 2H), 1.17 (d, *J* = 6.8 Hz, 3H); specific rotation [α]^23^D = +66.00 (c 0.20, CHCl_3_); enantiomeric excess = 99.68% ee (SFC, Chiralpak IC column; n-hexane/i-PrOH = 80:20 *v*/*v*; 1.0 mL/min; 25 °C). Compound **A3** was then synthesized via amide coupling between crude Compound **f** and 5-methylpyrazine-2-carboxylic acid (Compound **11**), using HATU as the coupling reagent and DIEA as the non-nucleophilic base in anhydrous DMF. Upon completion, the reaction was quenched with ice-cold water, extracted with ethyl acetate (EtOAc), washed with brine, dried over anhydrous Na_2_SO_4_, filtered, and concentrated under reduced pressure. Purification by flash column chromatography (petroleum ether/EtOAc = 1:1, *v*/*v*) afforded Compound **A3** as a white solid (35 mg, 59.00% yield over two steps).

High-resolution mass spectrometry (HRMS) data were fully consistent with the expected molecular formula, showing a protonated molecular ion at *m*/*z* 310.90 ([M+H]^+^; calculated for C_17_H_16_N_4_O_2_: 311.14). HPLC analysis (retention time = 5.413 min) indicated a purity of 98.83%. As shown in the Supplementary Materials (ESM_F3), the ^1^H NMR spectrum (400 MHz, DMSO-d_6_) displayed characteristic resonances: δ 9.06 (d, *J* = 1.4 Hz, 1H), 8.95 (t, *J* = 5.6 Hz, 1H), 8.66 (dd, *J* = 1.5, 0.7 Hz, 1H), 7.95 (s, 1H), 7.28 (d, *J* = 7.4 Hz, 1H), 7.17 (t, *J* = 7.7 Hz, 1H), 7.03 (t, *J* = 7.3 Hz, 1H), 4.73 (hept, *J* = 7.1 Hz, 1H), 4.44 (dd, *J* = 16.6, 5.8 Hz, 1H), 4.26 (dd, *J* = 16.8, 5.6 Hz, 1H), 3.41–3.33 (m, 1H), 2.67 (d, *J* = 16.2 Hz, 1H), 2.60 (s, 3H), 1.25 (d, *J* = 6.3 Hz, 3H), all in full agreement with the assigned structure. ^13^C NMR (101 MHz, DMSO-d_6_) δ 166.56, 163.63, 157.69, 143.56, 142.83, 142.12, 129.84–131.76 (br, 1C), 127.56, 125.31–126.53 (br, 1C), 124.31, 116.41–118.33 (br, 1C), 54.08–55.73 (br, 1C), 41.78–42.92 (br, 1C), 35.65–36.83 (br, 1C), 21.88; specific rotation [α]^23^D = +45.93 (c 1.00, CHCl_3_); enantiomeric excess = 99.92% ee (SFC, Chiralpak IC column; n-hexane/i-PrOH = 80:20 *v*/*v*; 1.0 mL/min; 25 °C).

### 2.7. Electrophysiological Results

**Figure 10:**
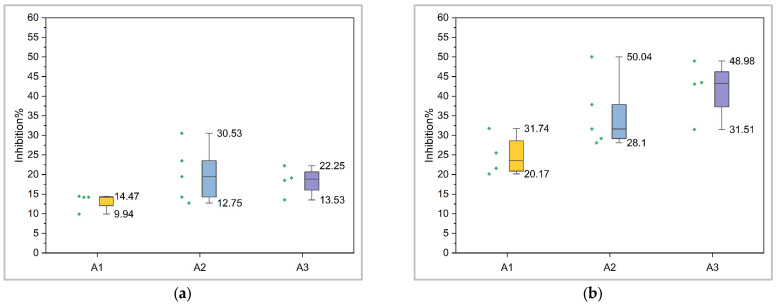
The inhibitory effect of the selected compounds on the current mediated by the GluN1-GluN2A receptor. (**a**): The inhibitory effect of the selected compounds at a concentration of 1 μM on the current mediated by the GluN1-GluN2A receptor; (**b**): The inhibitory effect of the selected compounds at a concentration of 10 μM on the current mediated by the GluN1-GluN2A receptor.

As shown in Figure 10, the inhibitory effects of the three potential antagonists on GluN1-GluN2A receptor-mediated currents at concentrations of 1 μM and 10 μM were relatively consistent and reproducible, indicating the reliability of the experiment. Preliminary results showed that at 1 μM, **A1**, **A2**, and **A3** inhibited channel activity by 13.20% ± 2.18%, 20.11% ± 7.23%, and 18.36% ± 3.61%, respectively. When the concentration was increased to 10 μM, the inhibitory effects increased to 24.26% ± 5.18%, 35.36% ± 9.03%, and 41.76% ± 7.34%, respectively. (S)-ketamine at 10 μM produced a strong inhibitory effect, with inhibition rates of 99.20% ± 0.71%, 99.33% ± 0.38%, and 98.64% ± 0.96%, respectively, confirming that the cells in each test group maintained normal receptor function and reactivity. All three compounds exhibited significant concentration-dependent inhibitory effects, indicating their potential as effective NMDA receptor antagonists. These findings suggest that **A1**, **A2**, and **A3** show preliminary but measurable inhibitory activity against the GluN1-GluN2A receptor, supporting further research on them as candidate therapeutic agents for central nervous system disorders, including depression. Future studies should include comprehensive in vivo evaluations using relevant animal models to confirm these effects and describe their pharmacological characteristics.

## 3. Discussion

Under pathological conditions, overactivation of NMDA receptors triggers excitotoxic cascades, leading to neuronal death and contributing to a range of neurological disorders [50,51]. This study successfully identified three potential antagonist candidates through the development of an integrated research framework integrating de novo molecular generation, mechanistic simulation, and experimental prediction. Comprehensive 200-ns molecular dynamics simulations demonstrated that these compounds exert their inhibitory effects through a mechanism analogous to that of (S)-ketamine-specifically, by forming hydrogen bonds with GluN1-Asn616 and engaging in hydrophobic interactions with GluN2A-Leu642 and GluN1-Val644, thereby preventing receptor overactivation. These three candidate molecules were synthesized using validated organic synthetic routes, and their inhibitory effects on GluN1-GluN2A receptor currents were assessed via patch-clamp experiments at two distinct concentrations.

The results of this study indicate that the three selected compounds can inhibit the current mediated by GluN1-GluN2A receptors at a concentration of 10 μM, confirming their potential functional antagonistic activity. However, due to the low solubility of the current compounds when preparing physiological experimental working solutions, the IC_50_ values could not be accurately determined. Therefore, subsequent work should focus on systematically modifying the structure to increase their solubility while maintaining or enhancing their targeted inhibitory activity. On this basis, dose-effect relationship studies should be conducted to obtain reliable IC_50_ parameters. Additionally, it is necessary to further explore the quantitative characteristics of their inhibitory efficacy, the correlation between in vivo pharmacokinetics and pharmacodynamics, and the safety of long-term administration. To promote the research on novel potential candidate drugs targeting the NMDA receptor.

## 4. Materials and Methods

### 4.1. Experimental Reagents

Ketamine was obtained from Shanghai Yuansi Standard Science and Technology Co., Ltd. (Shanghai, China); FBS was obtained from Avantor (Radnor, PA, USA); DMEM was obtained from CORNLNG (Corning, NY, USA); 0.25% Trypsin-EDTA was obtained from Gibco (Grand Island, NY, USA); and all other drugs and chemicals were obtained from MilliporeSigma (Burlington, MA, USA) and Solarbio (Beijing, China).

### 4.2. Preparation of Protein and Ligands

The crystal structure of the (S)-ketamine and GluN1-GluN2A receptor complex was obtained from the RCSB Protein Data Bank (PDB), with the PDB ID of 7EU7 and a resolution of 3.5 Å [33]. To select the GluN1-GluN2A receptor, this study evaluated multiple criteria, including resolution, deposition date in the PDB, and the identity of bound small molecules. (S)-ketamine has a wide range of pharmacological effects, including the treatment of depression, suicidal attempts, and status epilepticus, as well as neuroprotection, anti-inflammation, anti-cancer effects, and analgesia [52]. Throughout the entire research process, (S)-ketamine was selected as the control compound for comparative analysis.

Prior to formal generation, this study performed preprocessing of the GluN1-GluN2A receptor (PDB ID: 7EU7) using Maestro V.15.2025 [43] to correct protonation states and minimize energy with the OPLS4 force field. Meanwhile, this study retrieved approximately 500 GluN1-GluN2A receptor inhibitors with half-maximal inhibitory concentration (IC_50_) values below 100 μM from the BindingDB (www.bindingdb.org (accessed on 23 May 2024)) [53] and ChEMBL (https://www.ebi.ac.uk/chembl*/* (accessed on 23 May 2024)) [54] databases, and used these ligands to define the physicochemical property range of candidate molecules. The detailed information of these ligands is available in Supplementary Materials ESM_T6.

### 4.3. De Novo Design Using DrugFlow Platform

Among various structure-based drug design (SBDD) and ligand-based drug design (LBDD) approaches, de novo design stands out as a key strategy for constructing small molecules with optimal pharmacological properties [29]. In this study, the molecular factory module of DrugFlow [55] was employed to design potential GluN1-GluN2A receptor antagonists. DrugFlow is a multifunctional platform for de novo drug design and optimization driven by ligand-receptor interactions. It enables the construction of ligand compounds within a defined binding pocket based on interaction patterns, while automatically filtering out pan-assay interference compounds (PAINS) and structural alerts during molecular generation [56,57]. In this study, molecular design was guided by the three-dimensional crystal structure of the GluN1-GluN2A receptor in complex with (S)-ketamine.

The molecular factory module of DrugFlow supports multiple molecular generation strategies, including de novo design, R-group generation, linker design, and scaffold hopping, and integrates molecular generation models such as ResGen [36] and Delete [58]. In this study, the ResGen model was employed for de novo molecular design. ResGen is a three-dimensional molecular generation framework based on the receptor protein binding pocket, specifically designed for de novo drug molecule design against highly customized targets. Active compounds were utilized as input to fine-tune the ResGen model, thereby enhancing the similarity of the generated compounds in physicochemical properties to the reference actives. Using this approach, one hundred thousand novel molecules were successfully generated.

### 4.4. Virtual Screening Strategies

Virtual screening is a computational technique used to identify promising chemical candidates for specific targets from large-scale compound libraries. In this study, the novel molecules generated via de novo design were subjected to preliminary screening based on physicochemical property analysis. Meanwhile, RDKit [59] was employed to compute the ECFP4 fingerprints [37] of the chemical molecules for assessing the similarity between the novel compounds and the positive control drugs; only molecules with a similarity score below 0.50 were retained.

The study employed the AI-based molecular docking methods KarmaDock [39] and CarsiDock [41] to evaluate the binding affinity of the molecules to the receptor, enabling more efficient screening. KarmaDock [39] is an end-to-end artificial intelligence-based molecular docking and scoring method that surpasses most traditional approaches in both docking speed and accuracy, thereby enabling ultra-high-throughput virtual screening. CarsiDock [41] is a deep learning-based molecular docking method that leverages the Transformer architecture and draws inspiration from the model design of AlphaFold2. By incorporating conformational rationalization, this approach ensures high accuracy in predicting the atomic coordinates of ligand molecules. In this study, Maestro V.15.2025 [43] was employed to perform final molecular docking using the Glide XP method, analyze docking scores and binding free energies of the candidate molecules, and assess their synthetic feasibility. A total of approximately 100 molecules were selected for subsequent molecular dynamics simulations.

### 4.5. Molecular Dynamics Simulation

In this study, the refined structure was embedded into a pre-equilibrated palmitoyloleoylphosphatidylcholine (POPC) lipid bilayer by properly orienting the transmembrane domain (TMD) of the GluN1-GluN2A receptor within the membrane using CHARMM-GUI [60]. The generated system was solvated by TIP3P waters and neutralized with 0.2 M NaCl. Under the AMBER force field, preliminary dynamic simulations of the 100 selected molecules were performed using the GROMACS-2025 V.2025 software package and a custom gmx_batch.py script developed by the author of this study (https://github.com/kotori-y/gmx_batch (accessed on 3 October 2024)). For long-time molecular dynamics simulations, the system was first energy-minimized using the steepest descent algorithm. Subsequently, a series of treatments were applied to the proteins and ligands within the system, followed by equilibration under constant pressure and temperature (NPT ensemble; 310 K, 1 bar). During the simulation, positional restraints on the proteins were gradually released, and molecular dynamics parameter files were generated via CHARMM-GUI. Finally, the simulation trajectories were analyzed using GROMACS tools.

### 4.6. Binding Free Energy Calculations Using MM-PBSA

The energy contributions of residues within 4 Å of the ligand were analyzed based on binding free energy (ΔG_binding_) calculations performed using the MM-PBSA approach via gmx_MMPBSA [61], which excludes entropic contributions and provides an estimate of the free energy without accounting for entropy, rather than yielding the full enthalpic component. Based on the equilibrated dynamic trajectories of the system, a total of 50 snapshots were extracted from the last 2 ns of trajectory at 40-ps intervals. Electrostatic energy (ΔE_EL_) and van der Waals energy (ΔE_VDWAALS_) were calculated using the molecular mechanics force field, while polar solvation energy (ΔE_PB_) was computed with the Poisson-Boltzmann solver (APBS). Nonpolar solvation energy (ΔE_NPOLAR_) was estimated using the solvent-accessible surface area (SASA) method.

### 4.7. Synthetic Procedures

#### 4.7.1. Synthesis of Compound **A1** 

The synthetic procedure for compound **A1** is outlined below:


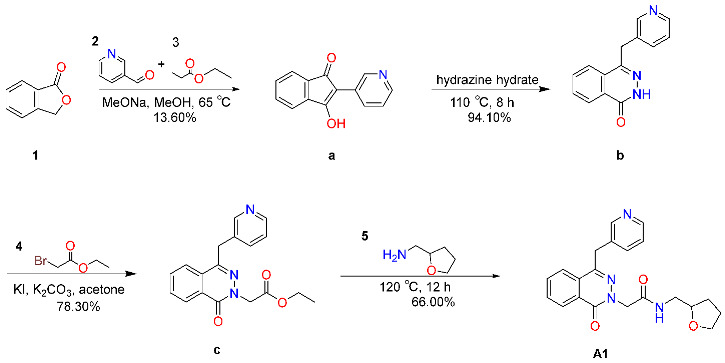


A solution of compound **1** in MeOH was added compound **2**, compound **3** and MeONa at 25 °C, stirred at 25 °C for 0.5 h, then the temperature was raised to 65 °C for reaction for 12 h; the reaction mixture was concentrated to remove MeOH, extracted with EtOAc, washed with H_2_O, the combined aqueous phase was adjusted to pH = 3 with 3 M HCl and stirred for 0.5 h, and the filtrate was concentrated to obtain compound **a**; a solution of compound **a** in hydrazine hydrate was stirred at 110 °C for 8 h, the reaction mixture was extracted with EtOAc, the combined organic layers were concentrated under reduced pressure to give a residue, which was further purified by flash silica gel chromatography (DCM:MeOH = 10:1) and triturated with MTBT, then the filtrate was concentrated to obtain compound **b**; a solution of compound **b** in DMF was added NaH at 0 °C, stirred at 10 °C for 0.5 h, then compound **4** was added at 10 °C, the temperature was raised to 25 °C for reaction for 3 h, the reaction mixture was extracted with EtOAc, the combined organic layers were concentrated under reduced pressure to give a residue, which was further purified by flash silica gel chromatography (DCM:MeOH = 10:1) and concentrated under reduced pressure to obtain compound **c**; a solution of compound **c** in compound **5** was stirred at 120 °C for 12 h until compound **c** was completely consumed, the reaction mixture was extracted with DCM, the combined organic layers were concentrated under reduced pressure to give a residue, which was purified by trituration (EtOA:MTBE = 5:1), then filtrated and the filter cake was concentrated to afford **A1**.

#### 4.7.2. Synthesis of Compound **A2** 

The synthetic procedure for compound **A2** is outlined below:


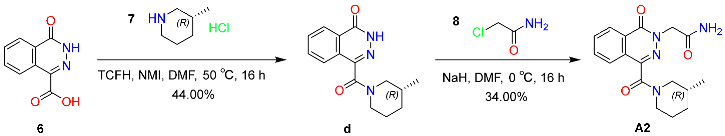


To a solution of 4-oxo-3,4-dihydrophthalazine-1-carboxylic acid (compound **6**), TCFH and DIEA in DMF was added (R)-3-methylpiperidine hydrochloride (compound **7**); the reaction mixture was stirred at 50 °C for 16 h under N_2_ and purified by Pre-HPLC (0.04% Ammonium formate in H_2_O/ACN) to give compound **d**; to a solution of (R)-4-(3-methylpiperidine-1-carbonyl)phthalazin-1(2H)-one (3) in DMF was added NaH, the reaction mixture was stirred at rt for 0.5 h under N_2_, then 2-chloroacetamide (compound **8**) was added and stirred for another 16 h, the resulting mixture was quenched with H_2_O and purified by Pre-HPLC (0.04% Ammonium formate in H_2_O/ACN) to give **A2**.

#### 4.7.3. Synthesis of Compound **A3** 

The synthetic procedure for compound **A3** is outlined below:


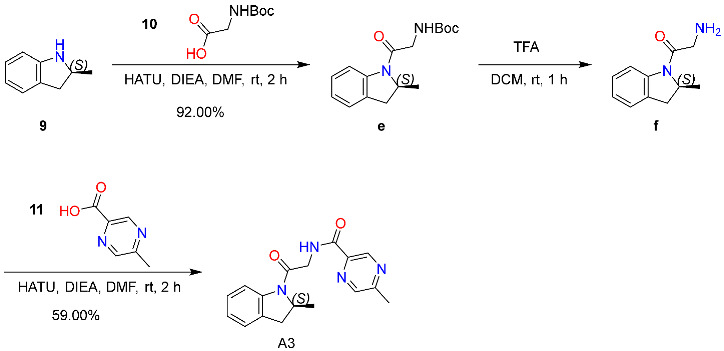


To a solution of 2-((tert-butoxycarbonyl)amino)acetic acid (compound **10**), HATU and DIEA in DMF was added (S)-2-methylindoline (compound **9**); the reaction mixture was stirred at room temperature (rt) for 2 h under N_2_, quenched with H_2_O, extracted with EtOAc, the combined organic layers were washed with brine, dried over anhydrous Na_2_SO_4_, filtered, concentrated and purified by Pre-TLC (PE/EtOAc = 3/1) to give compound **e**; to a solution of (S)-tert-butyl (2-(2-methylindolin-1-yl)-2-oxoethyl)carbamate (compound **e**) in DCM was added TFA, the reaction mixture was stirred at rt for 1 h under N_2_ and concentrated to give crude compound **f** for next step; to a solution of (S)-2-amino-1-(2-methylindolin-1-yl)ethenone (compound **f**, crude) and DIEA in DMF was added 5-methylpyrazine-2-carboxylic acid (compound **11**) and HATU, the reaction mixture was stirred at rt for 2 h under N_2_, quenched with H_2_O, extracted with EtOAc, the combined organic layers were washed with brine, dried over anhydrous Na_2_SO_4_, filtered, concentrated and purified by Pre-TLC (PE/EtOAc = 1/1) to give **A3**.

### 4.8. Electrophysiology

A HEK-293 cell line stably expressing GluN1-GluN2A receptors was cultured in DMEM supplemented with 10% FBS, 10 µg/mL Blasticidin, 100 µg/mL Zeocin, and 200 µg/mL Hygromycin B at 37 °C with 5% CO_2_ (humidified). Prior to patch-clamp recording, cells were dissociated with 0.25% Trypsin-EDTA, seeded on glass coverslips in 24-well plates, and induced with tetracycline plus 1 mM D-AP5 (to prevent premature receptor activation). Cells with confluence <80% were used for recordings after 18 h of induction.

For whole-cell patch-clamp, glass electrodes were filled with intracellular solution (10 mM NaCl, 110 mM CsMeS, 2 mM MgCl_2_·6H_2_O, 10 mM HEPES, 10 mM EGTA, 2 mM Na_2_-ATP, 0.2 mM Na_2_-GTP; pH 7.2, CsOH-adjusted) and immersed in extracellular solution (140 mM NaCl, 4 mM KCl, 2 mM CaCl_2_·2H_2_O, 10 mM HEPES, 5 mM D-glucose; pH 7.4, NaOH-adjusted) to measure baseline electrode resistance (Rpip). A high-resistance seal (>1 GΩ) was formed by gentle negative pressure after electrode contact with the cell membrane. Fast capacitance compensation was applied, followed by membrane patch rupture via gentle suction to establish a stable whole-cell configuration.

Cells were voltage-clamped at −60 mV in whole-cell mode. NMDA receptors were activated by bath application of 100 µM glycine + 100 µM L-glutamate until steady-state current was achieved (~100 s). Test compounds were co-applied cumulatively with agonists (each concentration perfused until current stabilization, ~100 s, before increasing concentration). Then apply 10 µM of (S)-ketamine to verify whether the cells can be normally inhibited. After compound washout with extracellular solution, agonist-induced current recovery was monitored to assess inhibition reversibility. Steady-state current amplitude at each concentration was normalized to the control (100 µM glycine + 100 µM L-glutamate alone), and inhibition rates were calculated to quantify compound potency.

$$Inhibition\%=\left(1-\frac{{Current}_{agonist+compound}}{{Current}_{agonist}}\right)\;*\;100\%$$

For each concentration, inhibition rates were analyzed by calculating the mean, and standard deviation (SD). Data are expressed as mean ± SD to represent both central tendency and the precision of the estimated values.

## 5. Conclusions

This study identified three structurally novel, high-affinity, and drug-like GluN1-GluN2A receptor antagonists with robust in vitro inhibitory activity by integrating computer-aided drug design, mechanistic molecular modeling, and experimental validation. These findings provide new drug target candidates for the treatment of central nervous system diseases such as depression and offer a feasible technical framework and theoretical reference for subsequent drug design targeting the GluN1-GluN2A receptor. Future work must prioritize comprehensive in vivo pharmacodynamic profiling, rigorous toxicological assessment, and formulation optimization to confirm target engagement, therapeutic window, and pharmacokinetic suitability, thereby enabling evidence-based progression from hit compounds to preclinical candidate drugs and accelerating the development of novel, mechanism-driven therapies for NMDA receptor–associated neurological diseases.

## Figures and Tables

**Table 1:** ∆Gbinding of **A1**, **A2**, **A3**, and (S)-ketamine to receptors and four energy terms.

Molecule	ΔG_binding_	ΔE_EL_	ΔE_VDWAALS_	ΔE_NPOLAR_	ΔE_PB_
**A1**	−26.33	−14.57	−41.36	−4.64	34.23
**A2**	−21.49	−15.45	−37.49	−4.25	35.70
**A3**	−20.70	−9.19	−34.05	−4.02	26.55
(S)-ketamine	−18.98	−2.88	−26.73	−3.09	13.71

**Table 2:** The results of the pharmacokinetic and drug-likeness evaluations for these three potential antagonists.

Molecule	QED	MW	TPSA	log S	log P	HIA^−^	BBB^+^	CL_plasma_
**A1**	0.71	378.17	86.11	−2.78	1.36	4.29 × 10^6^	0.60	3.00
**A2**	0.90	328.15	98.29	−2.50	0.86	1.91 × 10^6^	0.80	3.14
**A3**	0.93	310.14	75.19	−1.79	1.34	5.27 × 10^5^	0.89	7.37
(S)-ketamine	0.86	237.09	29.10	−2.90	2.10	1.80 × 10^5^	0.99	7.55

**Table 3:** RMSD of GluN1-GluN2A Receptor bound with proposed inhibitors.

Complex	A1	A2	A3	(S)-Ketamine
Minimum	3.94 × 10^5^	4.93 × 10^5^	4.20 × 10^5^	4.29 × 10^5^
Maximum	0.76	0.56	0.46	0.87
Average	0.55	0.38	0.25	0.73

**Table 4:** RMSF of GluN1-GluN2A receptor bound with proposed inhibitors.

Complex		A1	A2	A3	(S)-Ketamine
Protein A chain	Minimum	0.10	0.10	0.11	0.09
	Maximum	0.34	0.37	0.39	0.36
	Average	0.17	0.18	0.21	0.18
Protein B chain	Minimum	0.12	0.09	0.12	0.10
	Maximum	0.48	0.46	0.63	0.51
	Average	0.23	0.20	0.22	0.22
Protein C chain	Minimum	0.10	0.09	0.10	0.10
	Maximum	0.43	0.37	0.44	0.38
	Average	0.21	0.17	0.20	0.19
Protein D chain	Minimum	0.10	0.11	0.10	0.10
	Maximum	0.51	0.38	0.53	0.57
	Average	0.20	0.18	0.22	0.22

**Table 5:** Radius of gyration of the proposed inhibitors and their complexes with the GluN1-GluN2A receptor.

Inhibitors/Complex		A1	A2	A3	(S)-Ketamine
Inhibitors	Minimum	0.32	0.34	0.42	0.26
	Maximum	0.47	0.37	0.46	0.27
	Average	0.36	0.36	0.45	0.26
Complex	Minimum	3.53	3.46	3.53	3.49
	Maximum	3.79	3.78	3.79	3.78
	Average	3.59	3.52	3.61	3.56

## Data Availability

Data are contained within the article and Supplementary Materials.

## Supplementary Materials

The following supporting information can be downloaded at: https://www.mdpi.com/article/10.3390/molecules31030522/s1.

## Abbreviations

The following abbreviations are used in this manuscript:

RCSB	Research collaboratory for structural bioinformatics
OPLS4	Optimized potentials for liquid simulations 4
ECFP4	Extended connectivity fingerprints 4
CHARMM-GUI	Chemistry at Harvard macromolecular mechanics graphical user interface
TIP3P	Three-point water model
AMBER	Assisted model building with energy refinement
GROMACS	Groningen machine for chemical simulation

## References

[1] Klodowski D.A., Goldenholz S.R., Goldenholz D.M. (2026). Epilepsy and Placebo: A Literature Review. Neurol. Clin..

[2] Malhi G.S., Mann J.J. (2018). Depression. Lancet.

[3] Raffard S., de Connor A., Freeman D., Bortolon C. (2023). Recent developments in the modeling and psychological management of persecutory ideation. L’encephale.

[4] Balmer G.L., Guha S., Poll S. (2025). Engrams across diseases: Different pathologies-unifying mechanisms?. Neurobiol. Learn. Mem..

[5] Feigin V.L., Vos T., Nichols E., Owolabi M.O., Carroll W.M., Dichgans M., Deuschl G., Parmar P., Brainin M., Murray C. (2020). The global burden of neurological disorders: Translating evidence into policy. Lancet Neurol..

[6] Capó T., Rebassa J.B., Raïch I., Lillo J., Badia P., Navarro G., Reyes-Resina I. (2025). Future Perspectives of NMDAR in CNS Disorders. Molecules.

[7] Seillier C., Lesept F., Toutirais O., Potzeha F., Blanc M., Vivien D. (2022). Targeting NMDA Receptors at the Neurovascular Unit: Past and Future Treatments for Central Nervous System Diseases. Int. J. Mol. Sci..

[8] Nakashima M., Suga N., Yoshikawa S., Matsuda S. (2024). Caveolae with GLP-1 and NMDA Receptors as Crossfire Points for the Innovative Treatment of Cognitive Dysfunction Associated with Neurodegenerative Diseases. Molecules.

[9] Hansen K.B., Yi F., Perszyk R.E., Furukawa H., Wollmuth L.P., Gibb A.J., Traynelis S.F. (2018). Structure, function, and allosteric modulation of NMDA receptors. J. Gen. Physiol..

[10] Paoletti P., Bellone C., Zhou Q. (2013). NMDA receptor subunit diversity: Impact on receptor properties, synaptic plasticity and disease. Nat. Rev. Neurosci..

[11] Booker S.A., Wyllie D.J. (2021). NMDA receptor function in inhibitory neurons. Neuropharmacology.

[12] Park D.K., Stein I.S., Zito K. (2022). Ion flux-independent NMDA receptor signaling. Neuropharmacology.

[13] Johnson L.R., Battle A.R., Martinac B. (2019). Remembering Mechanosensitivity of NMDA Receptors. Front. Cell. Neurosci..

[14] Jiang L., Liu N., Zhao F., Huang B., Kang D., Zhan P., Liu X. (2024). Discovery of GluN2A subtype-selective N-methyl-d-aspartate (NMDA) receptor ligands. Acta Pharm. Sin. B.

[15] Deep S.N., Mitra S., Rajagopal S., Paul S., Poddar R. (2019). GluN2A-NMDA receptor-mediated sustained Ca^2+^ influx leads to homocysteine-induced neuronal cell death. J. Biol. Chem..

[16] Yavi M., Lee H., Henter I.D., Park L.T., Zarate C.A. (2022). Ketamine treatment for depression: A review. Discov. Ment. Health.

[17] Gjerulfsen C.E., Krey I., Klöckner C., Rubboli G., Lemke J.R., Møller R.S. (2024). Spectrum of NMDA Receptor Variants in Neurodevelopmental Disorders and Epilepsy. Methods Mol. Biol..

[18] Su T., Lu Y., Fu C., Geng Y., Chen Y. (2023). GluN2A mediates ketamine-induced rapid antidepressant-like responses. Nat. Neurosci..

[19] Michael A., Onisiforou A., Georgiou P., Koumas M., Powels C., Mammadov E., Georgiou A.N., Zanos P. (2025). (2R, 6R)-hydroxynorketamine prevents opioid abstinence-related negative affect and stress-induced reinstatement in mice. Br. J. Pharmacol..

[20] Zanos P., Brown K.A., Georgiou P., Yuan P., Zarate C.A., Thompson S.M., Gould T.D. (2023). NMDA Receptor Activation-Dependent Antidepressant-Relevant Behavioral and Synaptic Actions of Ketamine. J. Neurosci..

[21] Ebert B., Mikkelsen S., Thorkildsen C., Borgbjerg F.M. (1997). Norketamine, the main metabolite of ketamine, is a non-competitive NMDA receptor antagonist in the rat cortex and spinal cord. Eur. J. Pharmacol..

[22] Liu L., Huang H., Li Y., Zhang R., Wei Y., Wu W. (2021). Severe Encephalatrophy and Related Disorders From Long-Term Ketamine Abuse: A Case Report and Literature Review. Front. Psychiatry.

[23] Scotton E., Antqueviezc B., de Vasconcelos M.F., Dalpiaz G., Géa L.P., Goularte J.F., Colombo R., Rosa A.R. (2022). Is (R)-ketamine a potential therapeutic agent for treatment-resistant depression with less detrimental side effects? A review of molecular mechanisms underlying ketamine and its enantiomers. Biochem. Pharmacol..

[24] Ye S., Han Y., Wei Z., Li J. (2023). Binding Affinity and Mechanisms of Potential Antidepressants Targeting Human NMDA Receptors. Molecules.

[25] Yang C., Kobayashi S., Nakao K., Dong C., Han M., Qu Y., Ren Q., Zhang J.C., Ma M., Toki H. (2018). AMPA Receptor Activation-Independent Antidepressant Actions of Ketamine Metabolite (S)-Norketamine. Biol. Psychiatry.

[26] Ding Y., Zhang H., Zhu X., Wu M., Yang L., Yao Z., Xie Q., Liu X., Li C. (2021). Safety, tolerability, pharmacodynamics, and pharmacokinetics of CJC-1134-PC in healthy Chinese subjects and type-2 diabetic subjects. Expert Opin. Investig. Drugs.

[27] Piazza M.K., Kavalali E.T., Monteggia L.M. (2024). Ketamine induced synaptic plasticity operates independently of long-term potentiation. Neuropsychopharmacology.

[28] Angel I., Perelroizen R., Deffains W., Adraoui F.W., Pichinuk E., Aminov E. (2025). KETAMIR-2, a new molecular entity and novel ketamine analog. Front. Pharmacol..

[29] Shinde P.B., Bhowmick S., Alfantoukh E., Patil P.C., Wabaidur S.M., Chikhale R.V., Islam M.A. (2020). De novo design based identification of potential HIV-1 integrase inhibitors: A pharmacoinformatics study. Comput. Biol. Chem..

[30] Kumar S., Chowdhury S., Kumar S. (2017). In silico repurposing of antipsychotic drugs for Alzheimer’s disease. BMC Neurosci..

[31] Li J., Zhang J., Guo R., Dai J., Niu Z., Wang Y., Wang T., Jiang X., Hu W. (2025). Progress of machine learning in the application of small molecule druggability prediction. Eur. J. Med. Chem..

[32] Evren A.E., Hıdır A., Kurban B., Özkan B.N.S., Levent S., Şahin A., Özkay Y., Gündoğdu-Karaburun N. (2025). Latest developments in small molecule analgesics: Heterocyclic scaffolds II. Future Med. Chem..

[33] Zhang Y., Ye F., Zhang T., Lv S., Zhou L., Du D., Lin H., Guo F., Luo C., Zhu S. (2021). Structural basis of ketamine action on human NMDA receptors. Nature.

[34] Yang Y., Cui Y., Sang K., Dong Y., Ni Z., Ma S., Hu H. (2018). Ketamine blocks bursting in the lateral habenula to rapidly relieve depression. Nature.

[35] Moda-Sava R.N., Murdock M.H., Parekh P.K., Fetcho R.N., Huang B.S., Huynh T.N., Witztum J., Shaver D.C., Rosenthal D.L., Alway E.J. (2019). Sustained rescue of prefrontal circuit dysfunction by antidepressant-induced spine formation. Science.

[36] Zhang O., Zhang J., Jin J., Zhang X., Hu R., Shen C., Cao H., Du H., Kang Y., Deng Y. (2023). ResGen is a pocket-aware 3D molecular generation model based on parallel multiscale modelling. Nat. Mach. Intell..

[37] Rogers D., Hahn M. (2010). Extended-Connectivity Fingerprints. J. Chem. Inf. Model..

[38] Chi X., Chen R., Yang X., He X., Pan Z., Yao C., Peng H., Yang H., Huang W., Chen Z. (2025). Discovery of Novel DDR1 Inhibitors through a Hybrid Virtual Screening Pipeline, Biological Evaluation and Molecular Dynamics Simulations. ACS Med. Chem. Lett..

[39] Zhang X., Zhang O., Shen C., Qu W., Chen S., Cao H., Kang Y., Wang Z., Wang E., Zhang J. (2023). Efficient and accurate large library ligand docking with KarmaDock. Nat. Comput. Sci..

[40] Adasme M.F., Linnemann K.L., Bolz S.N., Kaiser F., Salentin S., Haupt V.J., Schroeder M. (2021). PLIP 2021: Expanding the scope of the protein-ligand interaction profiler to DNA and RNA. Nucleic Acids Res..

[41] Cai H., Shen C., Jian T., Zhang X., Chen T., Han X., Yang Z., Dang W., Hsieh C.-Y., Kang Y. (2024). CarsiDock: A deep learning paradigm for accurate protein-ligand docking and screening based on large-scale pre-training. Chem. Sci..

[42] Shen C., Zhang X., Deng Y., Gao J., Wang D., Xu L., Pan P., Hou T., Kang Y. (2022). Boosting Protein-Ligand Binding Pose Prediction and Virtual Screening Based on Residue-Atom Distance Likelihood Potential and Graph Transformer. J. Med. Chem..

[43] Maestro (2025). Schrödinger, Inc. 15.0. https://www.schrodinger.com.

[44] Ertl P., Schuffenhauer A. (2009). Estimation of synthetic accessibility score of drug-like molecules based on molecular complexity and fragment contributions. J. Cheminform..

[45] Wang E., Sun H., Wang J., Wang Z., Liu H., Zhang J.Z., Hou T. (2019). End-point binding free energy calculation with MM/PBSA and MM/GBSA: Strategies and applications in drug design. Chem. Rev..

[46] Swetha R., Sharma A., Singh R., Ganeshpurkar A., Kumar D., Kumar A., Singh S.K. (2022). Combined ligand-based and structure-based design of PDE 9A inhibitors against Alzheimer’s disease. Mol. Divers..

[47] Rajasekhar S., Das S., Karuppasamy R., Musuvathi Motilal B., Chanda K. (2022). Identification of novel inhibitors for Prp protein of Mycobacterium tuberculosis by structure based drug design, and molecular dynamics simulations. J. Comput. Chem..

[48] Fu L., Shi S., Yi J., Wang N., He Y., Wu Z., Peng J., Deng Y., Wang W., Wu C. (2024). ADMETlab 3.0: An updated comprehensive online ADMET prediction platform enhanced with broader coverage, improved performance, API functionality and decision support. Nucleic Acids Res..

[49] Rajasekhar S., Karuppasamy R., Chanda K. (2021). Exploration of potential inhibitors for tuberculosis via structure-based drug design, molecular docking, and molecular dynamics simulation studies. J. Comput. Chem..

[50] Granzotto A., d’Aurora M., Bomba M., Gatta V., Onofrj M., Sensi S.L. (2022). Long-Term Dynamic Changes of NMDA Receptors Following an Excitotoxic Challenge. Cells.

[51] Ehinger R., Kuret A., Matt L., Frank N., Wild K., Kabagema-Bilan C., Bischof H., Malli R., Ruth P., Bausch A.E. (2021). Slack K+ channels attenuate NMDA-induced excitotoxic brain damage and neuronal cell death. FASEB J..

[52] Mohammad Shehata I., Masood W., Nemr N., Anderson A., Bhusal K., Edinoff A.N., Cornett E.M., Kaye A.M., Kaye A.D. (2022). The Possible Application of Ketamine in the Treatment of Depression in Alzheimer’s Disease. Neurol. Int..

[53] Liu T., Hwang L., Burley S.K., Nitsche C.I., Southan C., Walters W.P., Gilson M.K. (2025). BindingDB in 2024: A FAIR knowledgebase of protein-small molecule binding data. Nucleic Acids Res..

[54] Zdrazil B., Felix E., Hunter F., Manners E.J., Blackshaw J., Corbett S., De Veij M., Ioannidis H., Lopez D.M., Mosquera J.F. (2024). The ChEMBL Database in 2023: A drug discovery platform spanning multiple bioactivity data types and time periods. Nucleic Acids Res..

[55] Shen C., Song J., Hsieh C.-Y., Cao D., Kang Y., Ye W., Wu Z., Wang J., Zhang O., Zhang X. (2024). DrugFlow: An AI-driven One-Stop Platform for Innovative Drug Discovery. J. Chem. Inf. Model..

[56] Jiang D., Lei T., Wang Z., Shen C., Cao D., Hou T. (2020). ADMET evaluation in drug discovery. 20. Prediction of breast cancer resistance protein inhibition through machine learning. J. Cheminform..

[57] Xiong G., Wu Z., Yi J., Fu L., Yang Z., Hsieh C., Yin M., Zeng X., Wu C., Lu A. (2021). ADMETlab 2.0: An integrated online platform for accurate and comprehensive predictions of ADMET properties. Nucleic Acids Res..

[58] Zhao H., Zhang H., Zhang X., Su Q., Du H., Shen C., Li D., Wang Z., Pan P., Chen G. (2023). Delete: Deep Lead Optimization Enveloped in Protein Pocket through Unified Deleting Strategies and a Structure-aware Network. arXiv.

[59] RDKit: Open-Source Cheminformatics. https://www.rdkit.org.

[60] Jo S., Kim T., Iyer V.G., Im W. (2008). CHARMM-GUI: A web-based graphical user interface for CHARMM. J. Comput. Chem..

[61] Valdés-Tresanco M.S., Valdés-Tresanco M.E., Valiente P.A., Moreno E. (2021). gmx_MMPBSA: A New Tool to Perform End-State Free Energy Calculations with GROMACS. J. Chem. Theory Comput..

