# Supplementary material for: De Novo Generation-Based Design of Potential Computational Hits Targeting the GluN1-GluN2A Receptor
Source: Molecules. 2026 Feb 2;31(3):522. doi: 10.3390/molecules31030522 (PMC12900030; doi:10.3390/molecules31030522)
Supplement: Supplementary file 1 [file molecules-31-00522-s001.zip › ESM_F1_Characterization of Compounds in Scheme 1/A1-HPLC.pdf]

HPLC Report

Sample Information
Instrument : HPLC-01
Sample Name : A1
Sample ID : P25111900029
Vial# : 76
Injection Volume : 0.7 uL
Method File : HPLC-0.07%TFA-5-95-1.5-02.lcm
Date Acquired : 17/Nov/2025 11:09:12 AM
Date Processed : 17/Nov/2025 11:35:10 AM

Method
Instrument : Shimadzu LC-20AD
Column : Shim-pack VP-ODS , 4.6 mm*50 mm , 5.0 um
Oven Temperature : 40 Flow Rate : 1.5000 mL/min
Mobile Phase : A : H2O+0.07%TFA
Mobile Phase : B : ACN

Time	Module	Command	Value
0.01	Pumps	B.Conc	5
4.20	Pumps	B.Conc	95
5.30	Pumps	B.Conc	95
5.31	Pumps	B.Conc	5
6.00	Controller	Stop	

Chromatogram
mAU

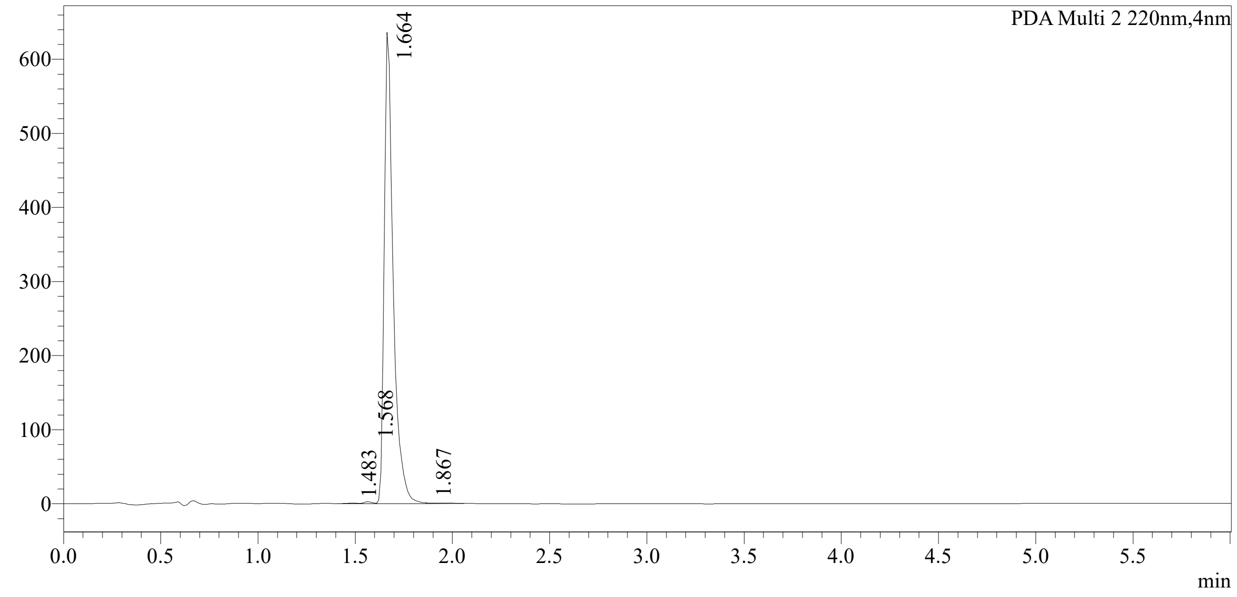

Peak Table
PDA Ch2 220nm

Peak#	Ret. Time	Area	Height	Area%
1	1.483	3687	900	0.178
2	1.568	8082	2675	0.391
3	1.664	2048204	636328	99.155
4	1.867	5681	973	0.275
Total		2065654	640876	100.000

Chromatogram

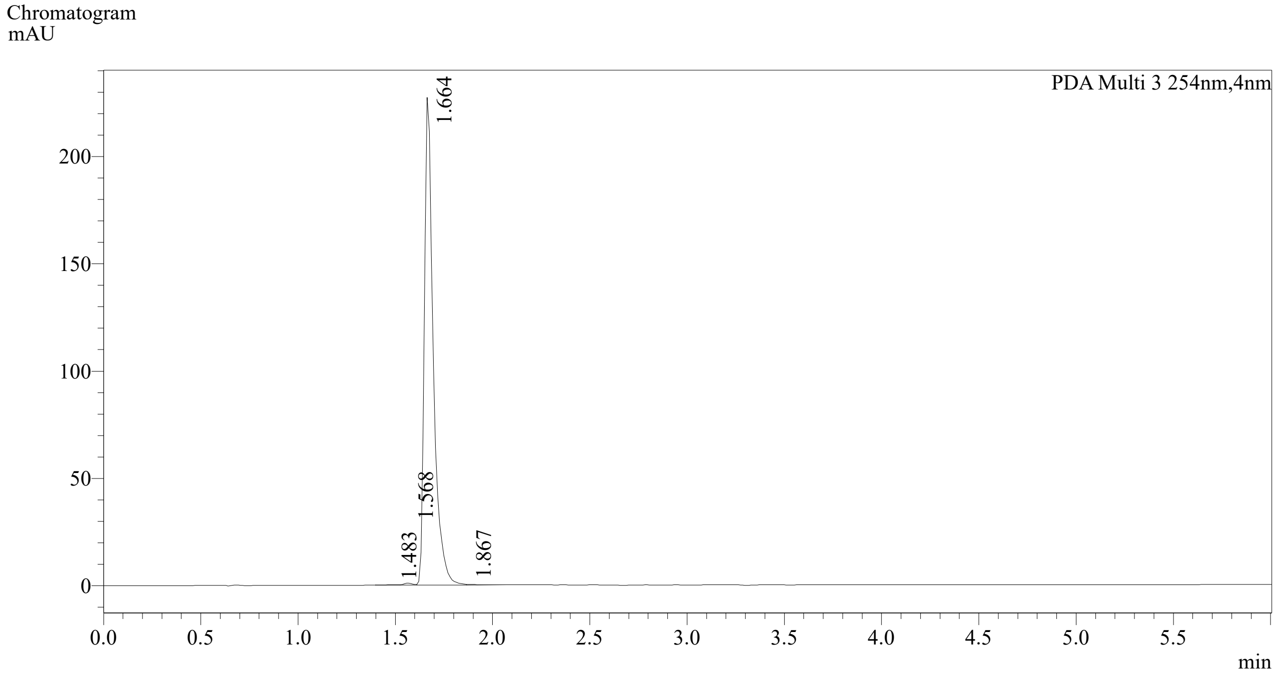

Peak Table
PDA Ch3 254nm

Peak#	Ret. Time	Area	Height	Area%
1	1.483	857	198	0.117
2	1.568	2773	912	0.378
3	1.664	727860	227225	99.313
4	1.867	1405	281	0.192
Total		732894	228616	100.000

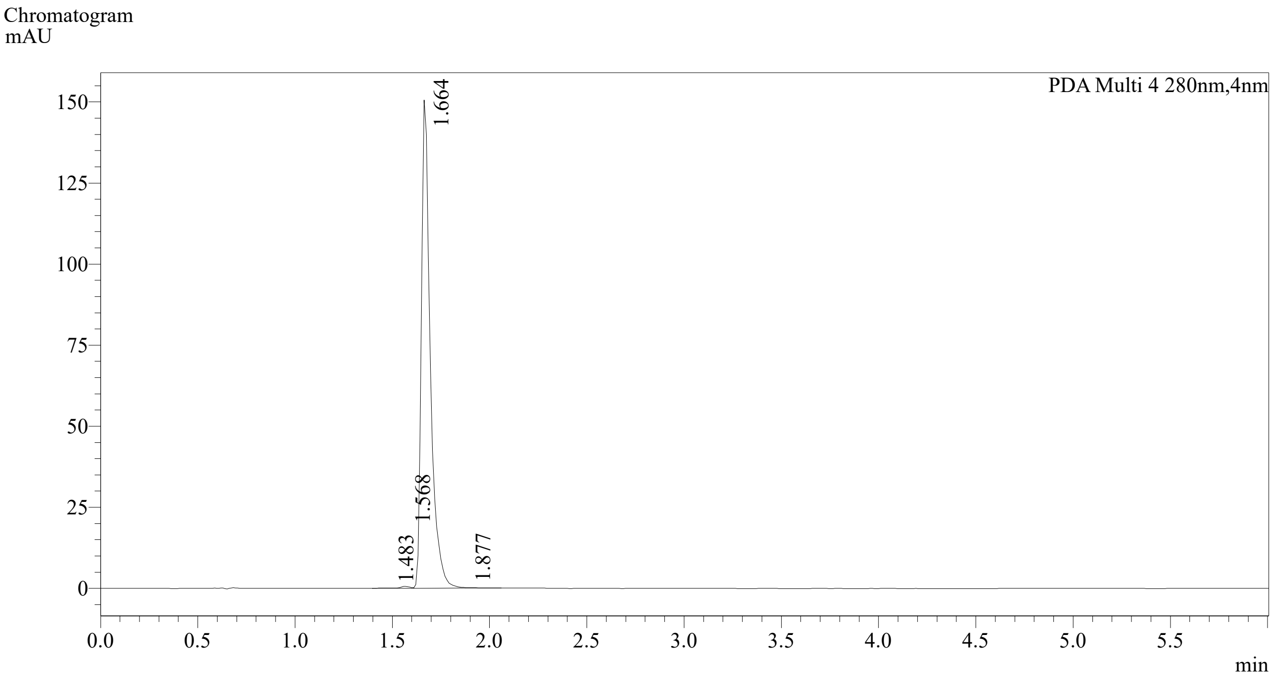

Peak Table
PDA Ch4 280nm

Peak#	Ret. Time	Area	Height	Area%
1	1.483	511	114	0.105
2	1.568	1799	602	0.371
3	1.664	482369	150532	99.349
4	1.877	849	171	0.175
Total		485528	151419	100.000
