# Supplementary material for: De Novo Generation-Based Design of Potential Computational Hits Targeting the GluN1-GluN2A Receptor
Source: Molecules. 2026 Feb 2;31(3):522. doi: 10.3390/molecules31030522 (PMC12900030; doi:10.3390/molecules31030522)

Formula $C_{21}H_{22}N_4O_3$	FW	378.4244

Acquisition Time (sec)	1.3763	***Comment***	20260123A191D-P25111900029			***Date***	24 Jan 2026 17:42:24
Date Stamp	24 Jan 2026 17:42:24						
File Name	C:\Users\Desktop\						
Frequency (MHz)	100.61	***Nucleus***	13C	***Number of Transients***	1800	***Origin***	Avance
Original Points Count	32768	***Owner***	nmrsu	***Points Count***	32768	***Pulse Sequence***	zgpg30
Receiver Gain	31.96	***SW(cyclical) (Hz)***	23809.52	***Solvent***	DMSO-d6	***Spectrum Offset (Hz)***	10061.2803
Spectrum Type	STANDARD	***Sweep Width (Hz)***	23808.80	***Temperature (degree C)***	26.323		

13C NMR (101 MHz, DMSO-d6) d ppm 167.43 (s, 1 C) 159.00 (s, 1 C) 150.21 (s, 1 C) 148.26 (s, 1 C) 144.72 (s, 1 C) 136.47 (s, 1 C) 134.11 (s, 1 C) 133.95 (s, 1 C) 132.28 (s, 1 C) 129.19 (s, 1 C) 128.10 (s, 1 C) 126.94 (s, 1 C) 126.03 (s, 1 C) 123.97 (s, 1 C) 77.54 (s, 1 C) 67.65 (s, 1 C) 53.68 (s, 1 C) 43.23 (s, 1 C) 35.17 (s, 1 C) 28.93 (s, 1 C) 25.62 (s, 1 C)

A1

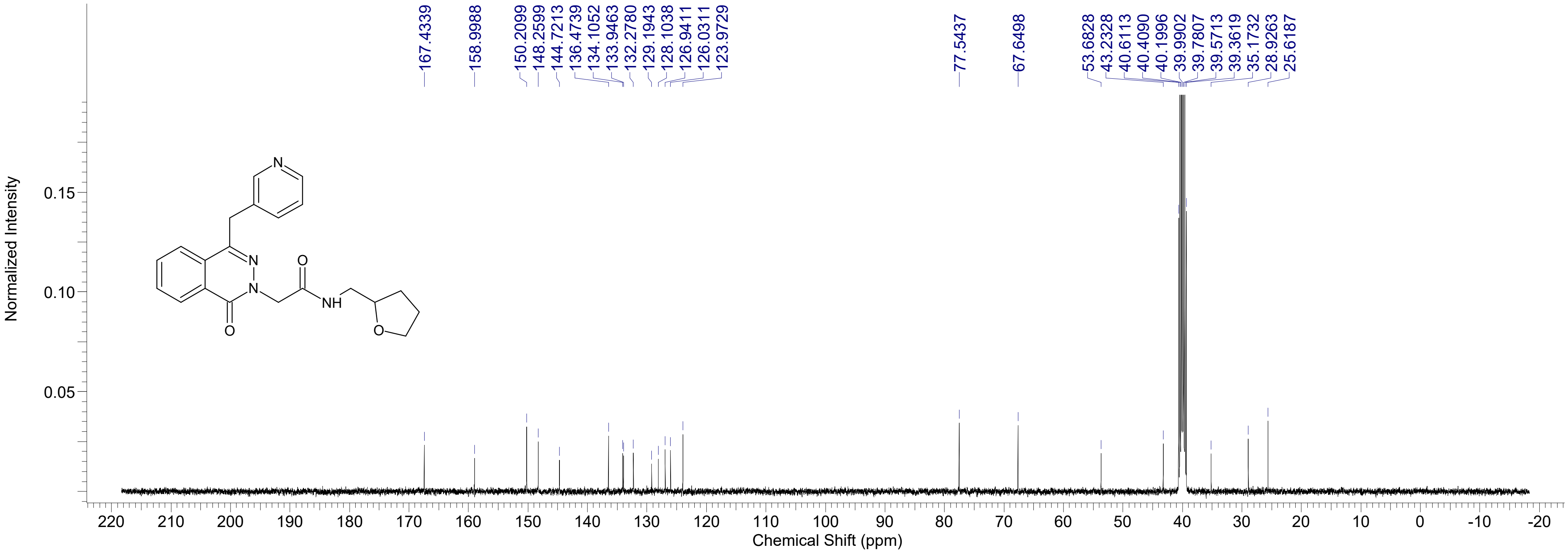

Supplement: Supplementary file 1 [file molecules-31-00522-s001.zip › ESM_F1_Characterization of Compounds in Scheme 1/A1_13C NMR.pdf]
