# Supplementary material for: De Novo Generation-Based Design of Potential Computational Hits Targeting the GluN1-GluN2A Receptor
Source: Molecules. 2026 Feb 2;31(3):522. doi: 10.3390/molecules31030522 (PMC12900030; doi:10.3390/molecules31030522)

LC-MS Report

Sample Information

Instrument	: LCMS-02
Sample Name	: A1
Sample ID	: P25111900029
Vial#	: 34
Injection Volume	: 0.8 µL
Method File	: LCMS-0.05%FA-5-95(+&-)-1.0-01.lcm
Date Acquired	: 17/Nov/2025 11:48:39 AM
Date Processed	: 17/Nov/2025 12:03:49 PM

Method
Instrument : Shimadzu LCMS-2020
Column : Shim-pack GIST C18-AQ , 2.1 mm*50 mm , 5.0 um
Oven Temperature : 40 Flow Rate : 1.0000 mL/min
Mobile Phase : A : H2O+0.05%FA
Mobile Phase : B : ACN

Time	Module	Command	Value
0.01	Pumps	B.Conc	5
2.00	Pumps	B.Conc	95
2.50	Pumps	B.Conc	95
2.51	Pumps	B.Conc	5
3.50	Controller	Stop	

Chromatogram

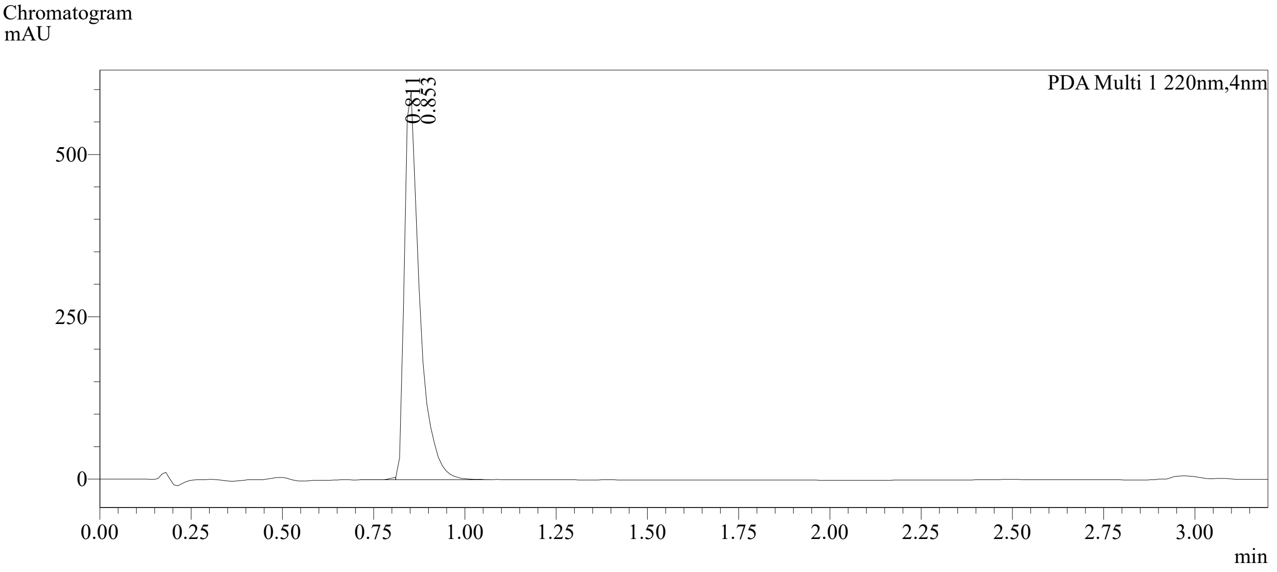

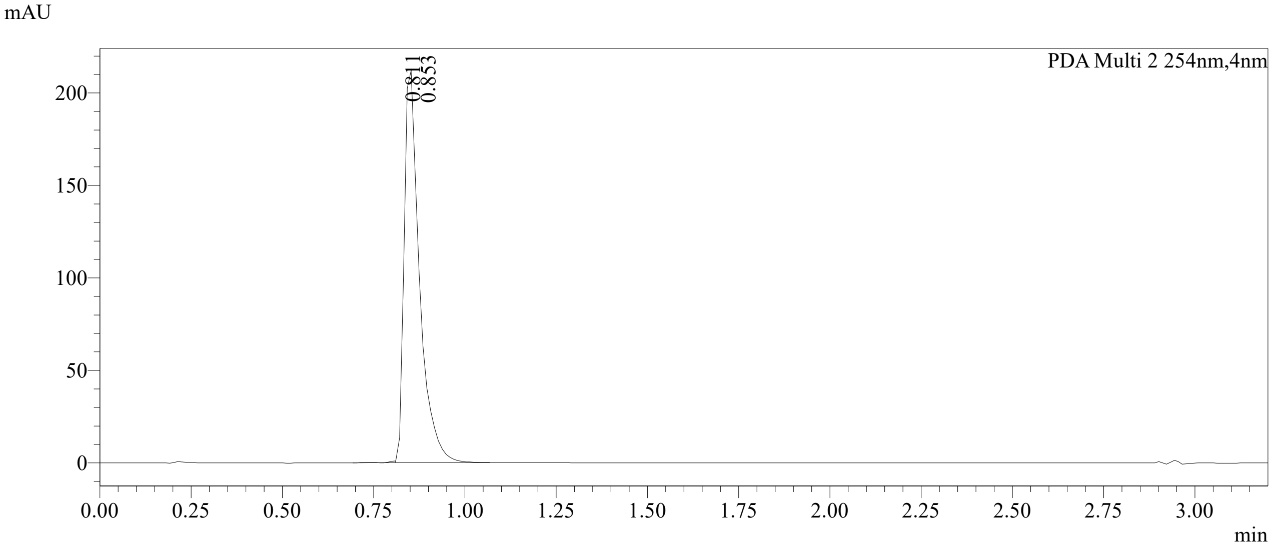

MS Chromatogram
Segment#1 (x10,000,000)

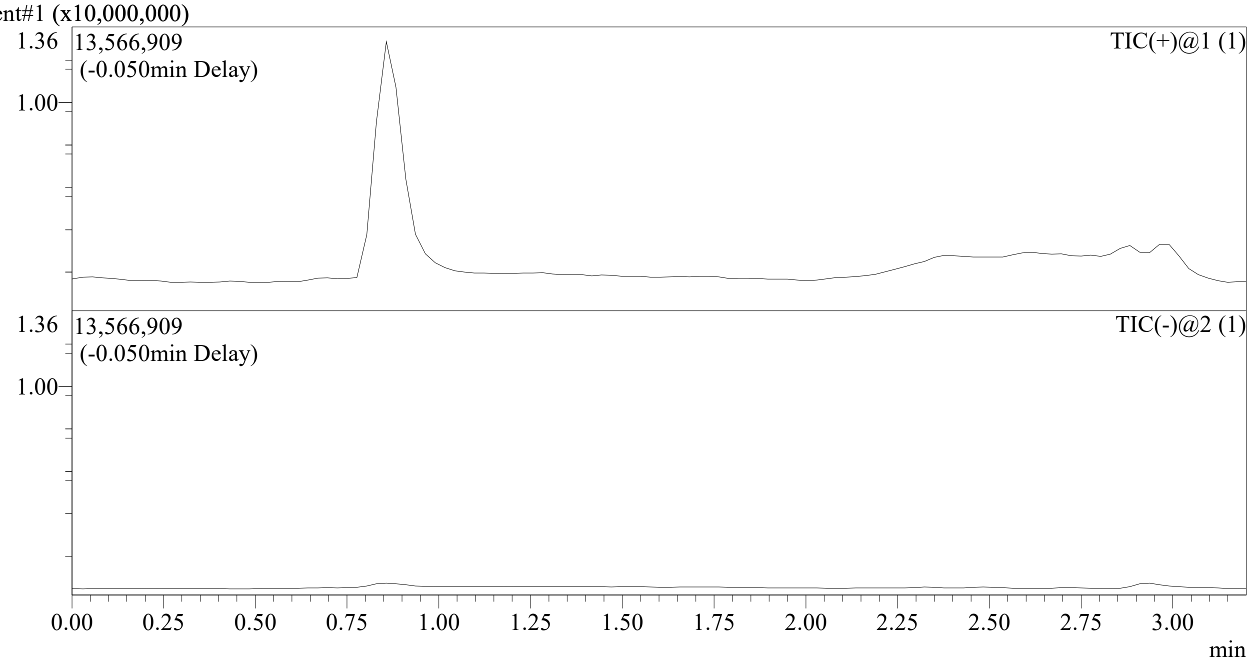

Peak Table
PDA Ch1 220nm

Peak#	Ret. Time	Height	Height%	Area	Area%
1	0.811	3307	0.551	4151	0.237
2	0.853	596962	99.449	1743997	99.763

PDA Ch2 254nm

Peak#	Ret. Time	Height	Height%	Area	Area%
1	0.811	1000	0.469	1283	0.206
2	0.853	212053	99.531	620938	99.794

MS Spectrum

MassPeaks:434
Spectrum Mode:Averaged 0.830-0.883(67-71) Base Peak:379.2(5238548)
BG Mode:Calc Segment 1 - Event 1

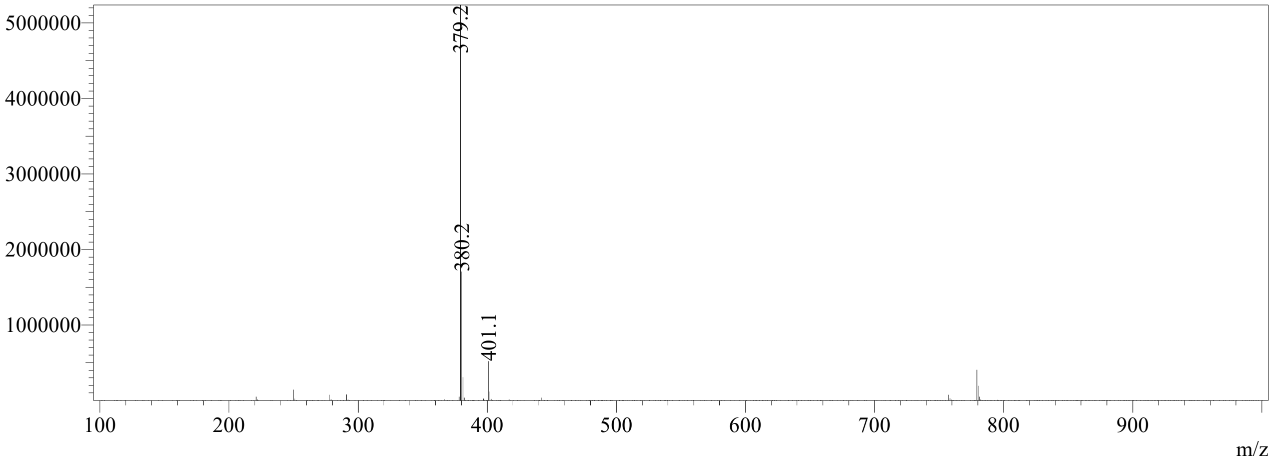

MassPeaks:504
Spectrum Mode:Averaged 0.843-0.897(68-72) Base Peak:249.0(63270)
BG Mode:Calc Segment 1 - Event 2

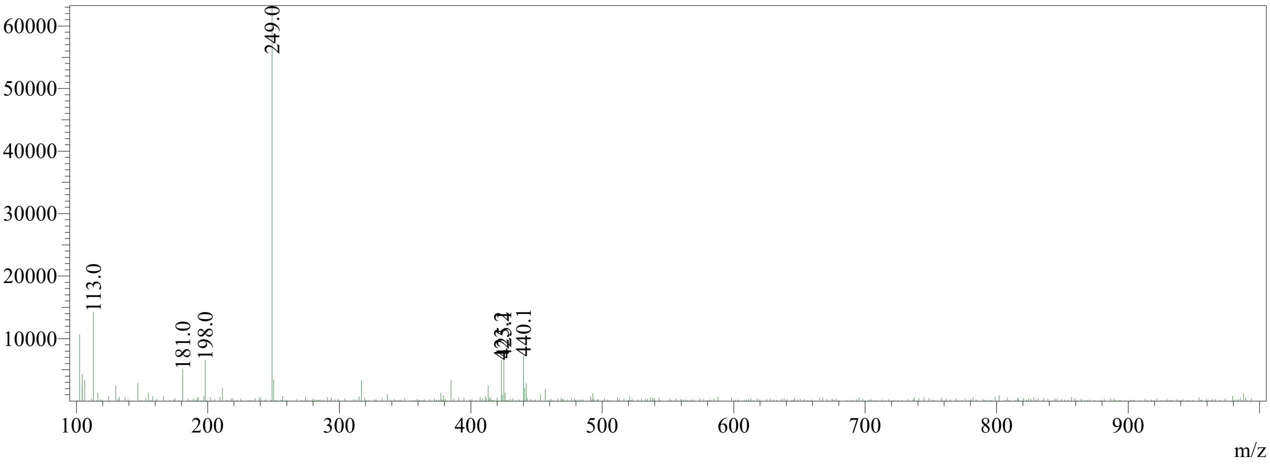

Supplement: Supplementary file 1 [file molecules-31-00522-s001.zip › ESM_F1_Characterization of Compounds in Scheme 1/A1_LC-MS.pdf]
