# Supplementary material for: De Novo Generation-Based Design of Potential Computational Hits Targeting the GluN1-GluN2A Receptor
Source: Molecules. 2026 Feb 2;31(3):522. doi: 10.3390/molecules31030522 (PMC12900030; doi:10.3390/molecules31030522)

LC-MS Report

Sample Information

Instrument	: LCMS-01
Sample Name	: Compound a
Sample ID	: LRY
Injection Volume	: 0.5 µL
Vial#	: 22
Method File	: LCMS-LC017-0.04%AB5-95(+)-01.lcm
Date Acquired	: 13/Jan/2026 1:40:40 PM
Modified Date	: 13/Jan/2026 1:44:12 PM

Method
Instrument : Shimadzu LCMS-2020
Column : Shim-pack GIST C18 , 2.1 mm*50 mm , 5.0 um
Oven Temperature : 40 Flow Rate : 1.0000 mL/min
Mobile Phase : A : H2O+0.04%TFA
Mobile Phase : B : ACN+0.02%TFA

Time	Module	Command	Value
0.01	Pumps	B.Conc	5
2.00	Pumps	B.Conc	95
2.50	Pumps	B.Conc	95
2.51	Pumps	B.Conc	5
3.50	Controller	Stop	

Chromatogram

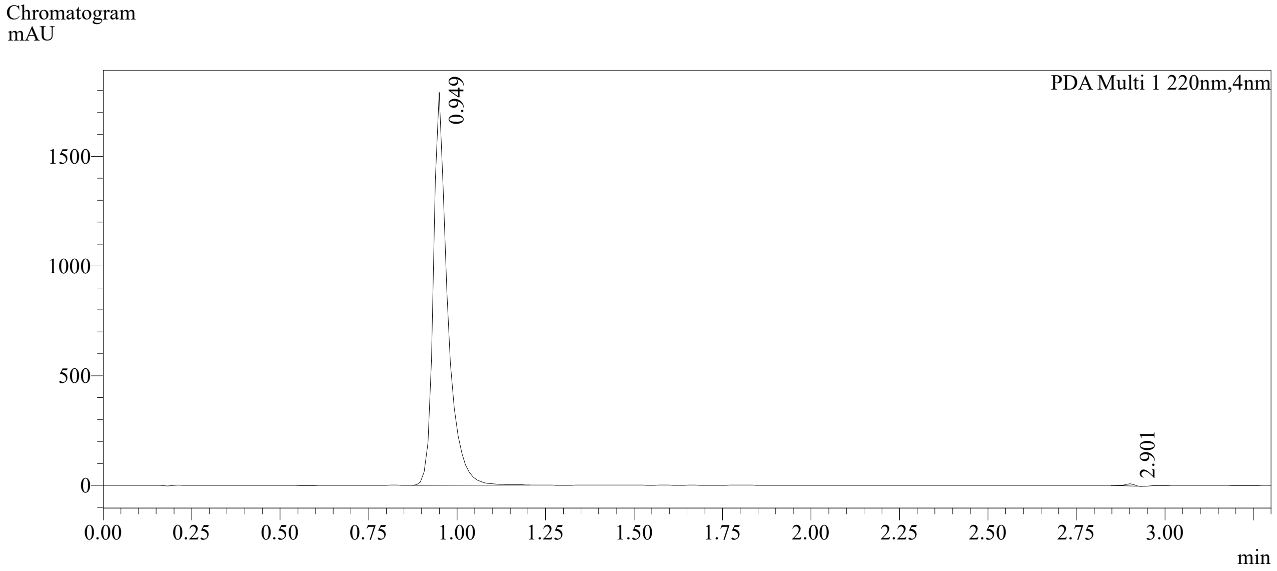

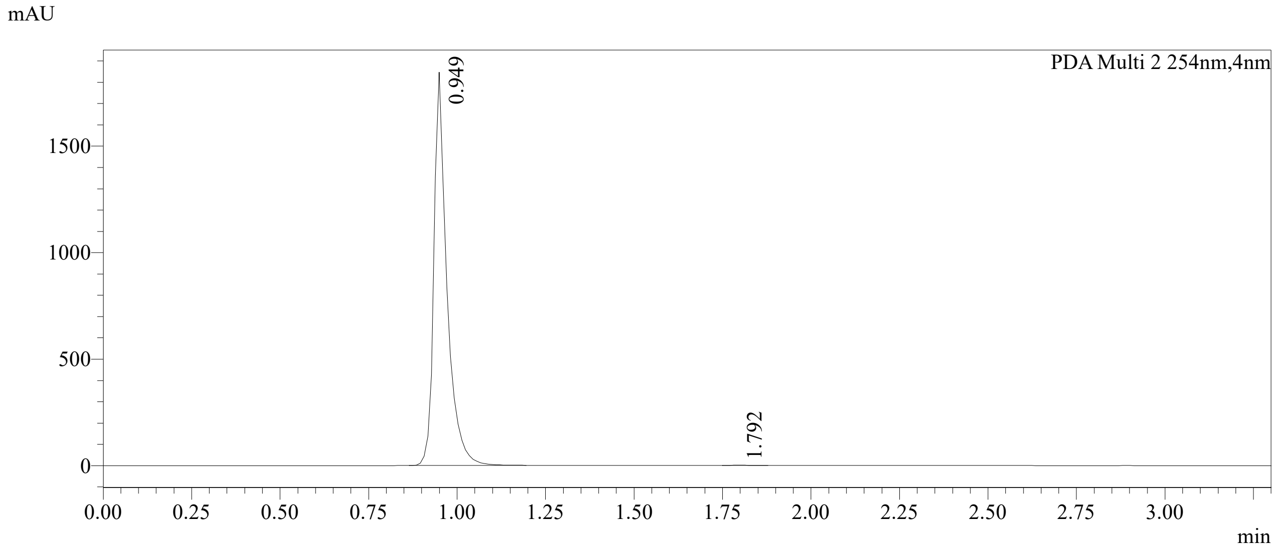

MS Chromatogram

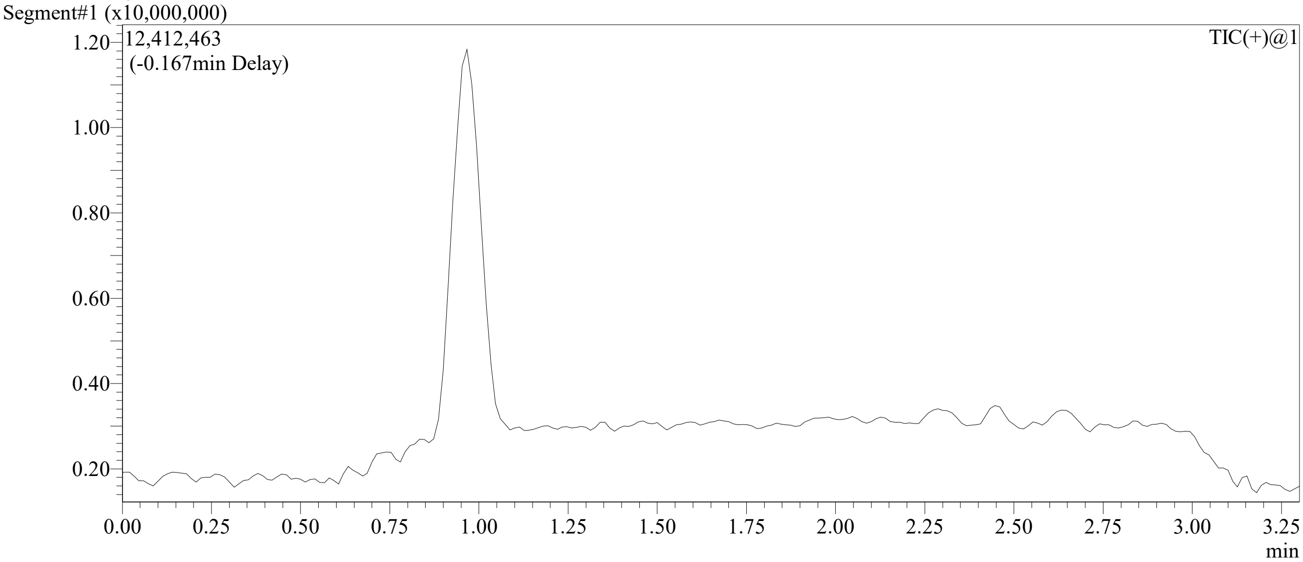

Peak Table

PDA Ch1 220nm

Peak#	Ret. Time	Height	Height%	Area	Area%
1	0.949	1790570	99.433	5001772	99.642
2	2.901	10212	0.567	17946	0.358

PDA Ch2 254nm

Peak#	Ret. Time	Height	Height%	Area	Area%
1	0.949	1847211	99.918	4702634	99.892
2	1.792	1520	0.082	5061	0.108

MS Spectrum

MassPeaks:811
Spectrum Mode:Averaged 0.940-0.967(84-86) Base Peak:224.1(3570290)
BG Mode:Calc Segment 1 - Event 1

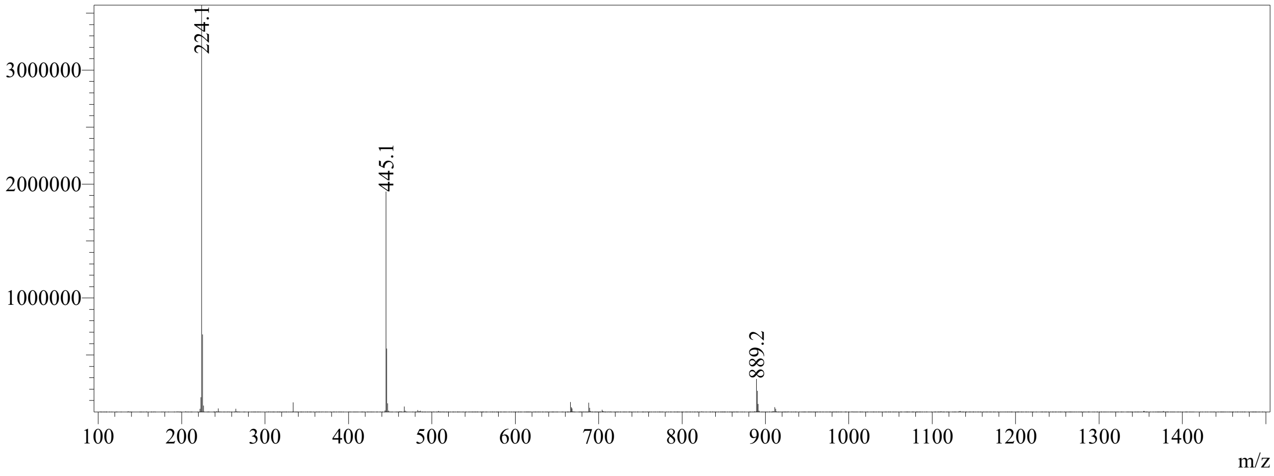

MassPeaks:685
Spectrum Mode:Averaged 2.887-2.913(230-232) Base Peak:130.2(22220)
BG Mode:Calc Segment 1 - Event 1

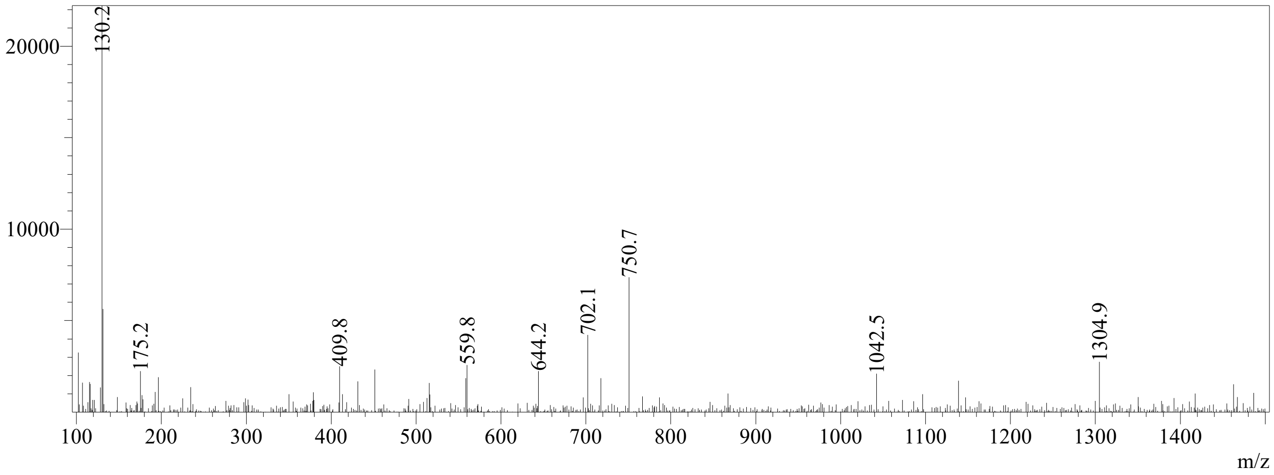

Supplement: Supplementary file 1 [file molecules-31-00522-s001.zip › ESM_F1_Characterization of Compounds in Scheme 1/Compound a_LC-MS.pdf]
