# Supplementary material for: De Novo Generation-Based Design of Potential Computational Hits Targeting the GluN1-GluN2A Receptor
Source: Molecules. 2026 Feb 2;31(3):522. doi: 10.3390/molecules31030522 (PMC12900030; doi:10.3390/molecules31030522)

LC-MS Report

Sample Information

Instrument	: LCMS-01
Sample Name	: Compound b
Sample ID	: LRY
Injection Volume	: 0.5 µL
Vial#	: 29
Method File	: LCMS-LC017-0.04%AB5-95(+)-01.lcm
Date Acquired	: 03/Nov/2025 1:52:46 PM
Modified Date	: 03/Nov/2025 1:56:17 PM

Method
Instrument : Shimadzu LCMS-2020
Column : Shim-pack GIST C18 , 2.1 mm*50 mm , 5.0 um
Oven Temperature : 40 Flow Rate : 1.0000 mL/min
Mobile Phase : A : H2O+0.04%TFA
Mobile Phase : B : ACN+0.02%TFA

Time	Module	Command	Value
0.01	Pumps	B.Conc	5
2.00	Pumps	B.Conc	95
2.50	Pumps	B.Conc	95
2.51	Pumps	B.Conc	5
3.50	Controller	Stop	

Chromatogram

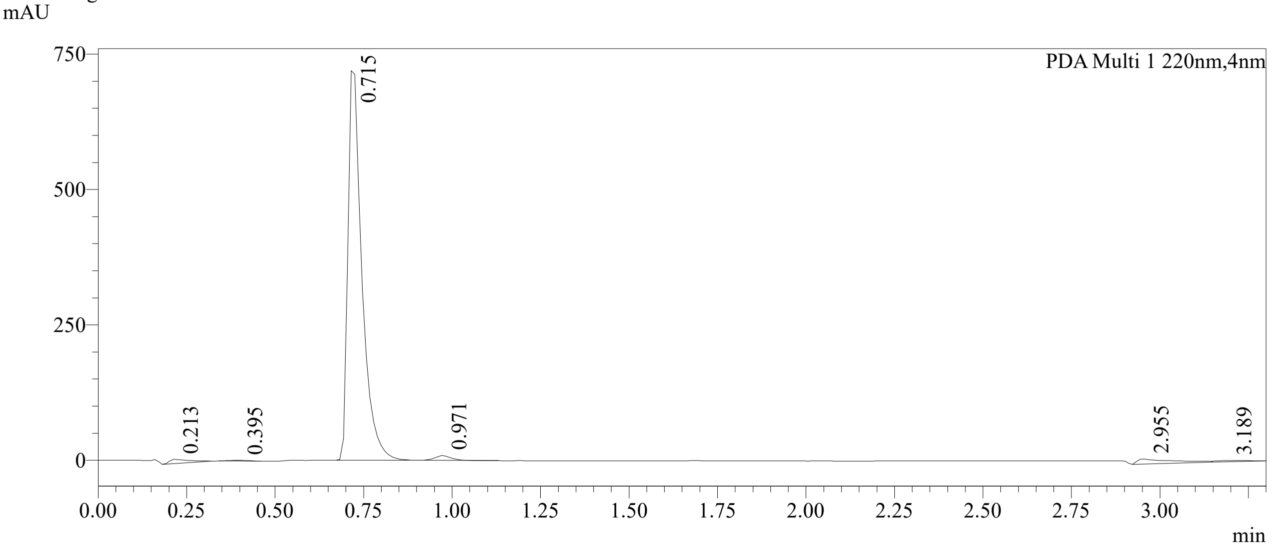

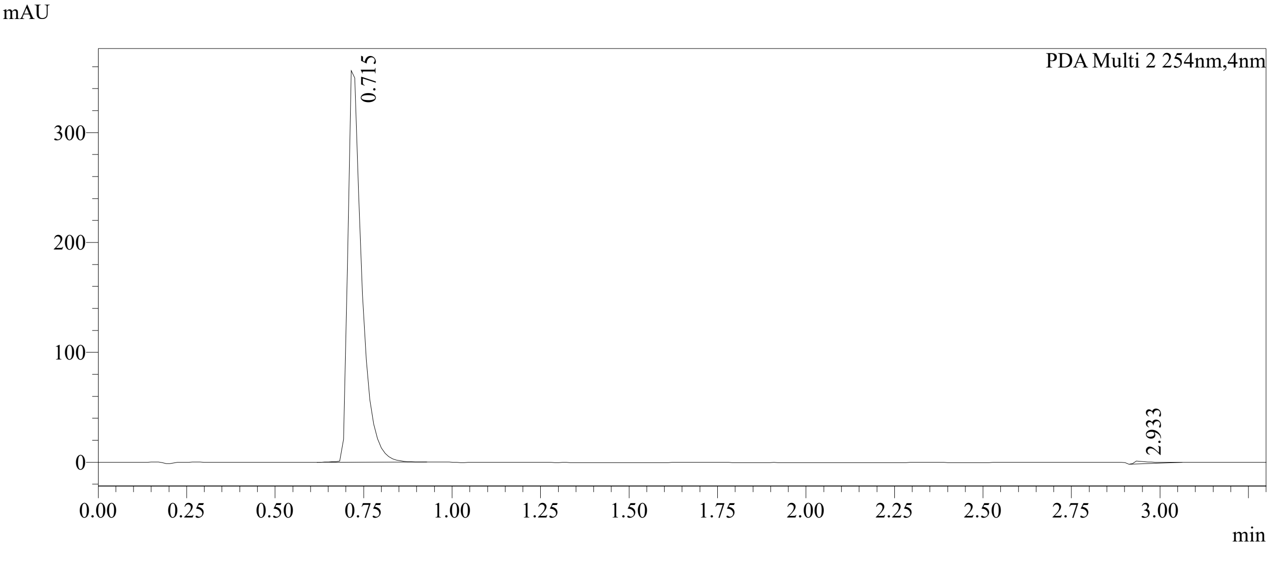

MS Chromatogram

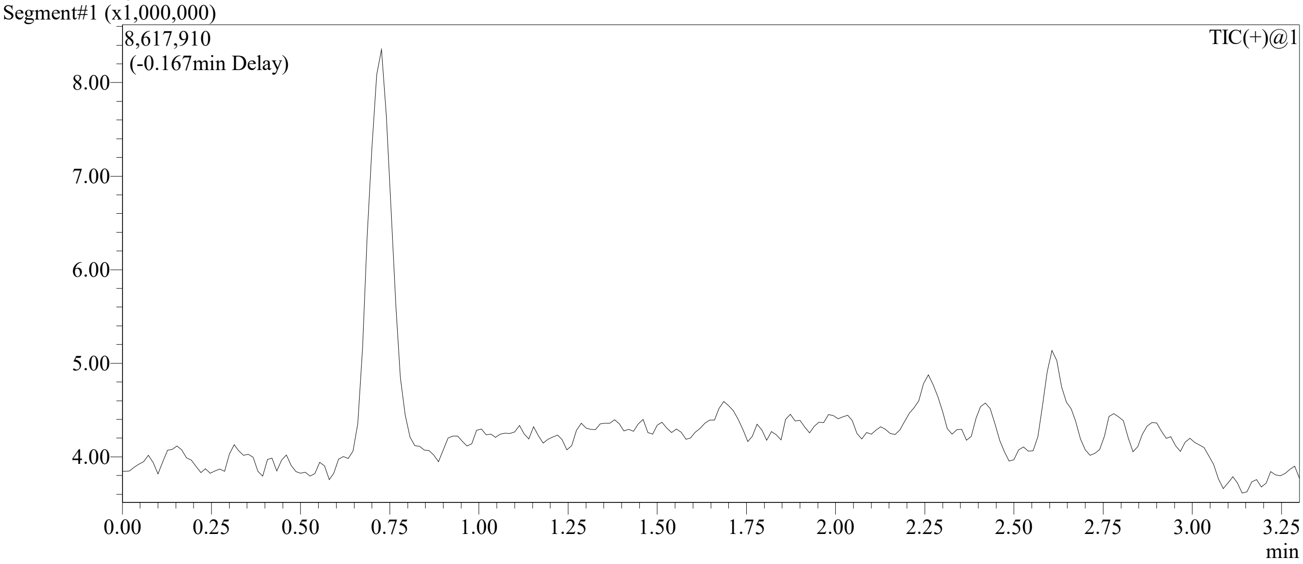

Peak Table

PDA Ch1 220nm

Peak#	Ret. Time	Height	Height%	Area	Area%
1	0.213	7534	1.005	28006	1.303
2	0.395	1436	0.192	5819	0.271
3	0.715	719469	96.006	2004319	93.281
4	0.971	9092	1.213	29661	1.380
5	2.955	9398	1.254	65748	3.060
6	3.189	2469	0.329	15125	0.704

PDA Ch2 254nm

Peak#	Ret. Time	Height	Height%	Area	Area%
1	0.715	356453	99.267	988416	99.115
2	2.933	2630	0.733	8824	0.885

MS Spectrum

MassPeaks:704
Spectrum Mode:Averaged 0.193-0.220(28-30) Base Peak:114.2(21051)
BG Mode:Calc Segment 1 - Event 1

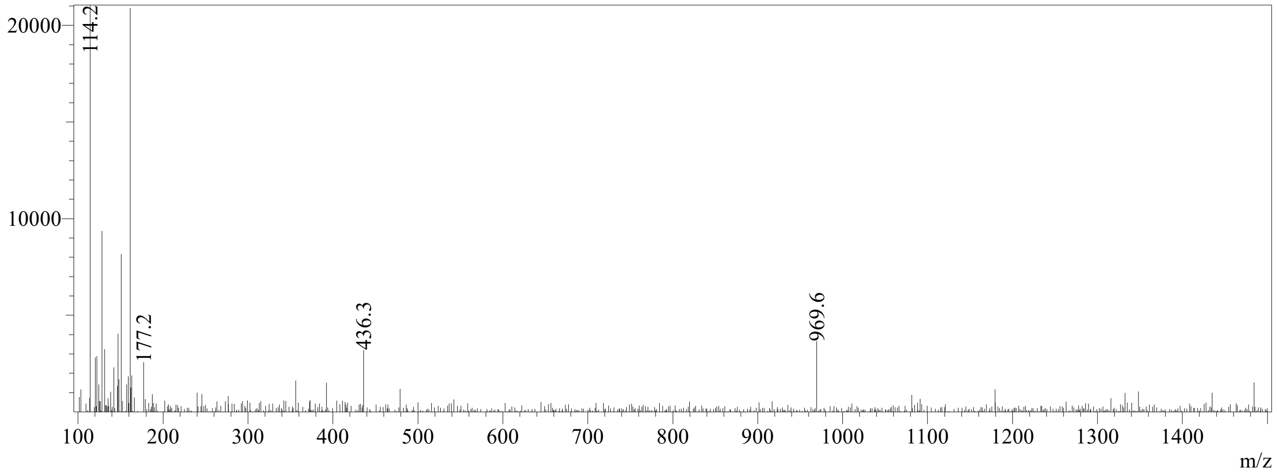

MassPeaks:699
Spectrum Mode:Averaged 0.380-0.407(42-44) Base Peak:161.2(20408)
BG Mode:Calc Segment 1 - Event 1

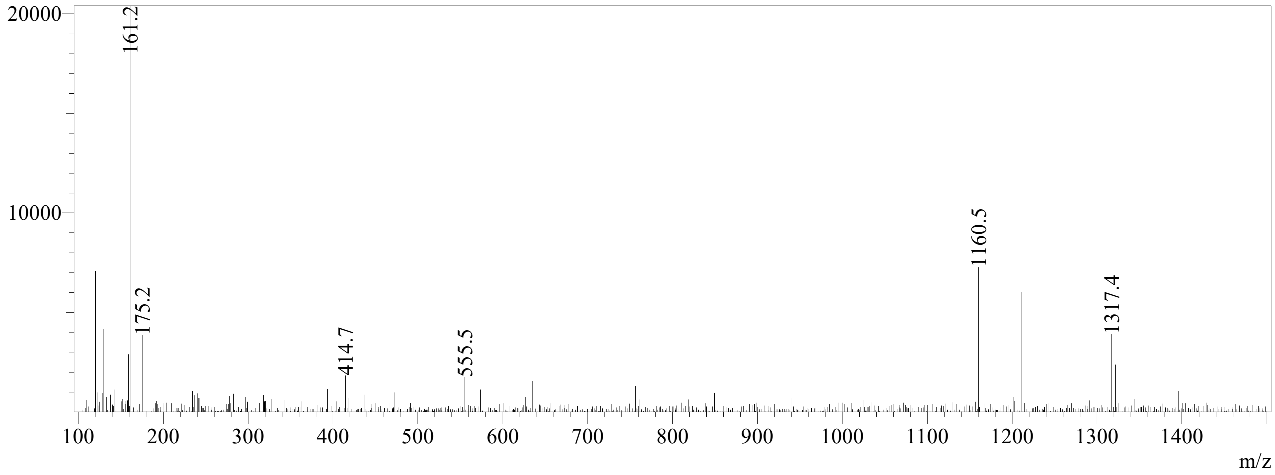

MassPeaks:892
Spectrum Mode:Averaged 0.700-0.727(66-68) Base Peak:238.1(1685840)
BG Mode:Calc Segment 1 - Event 1

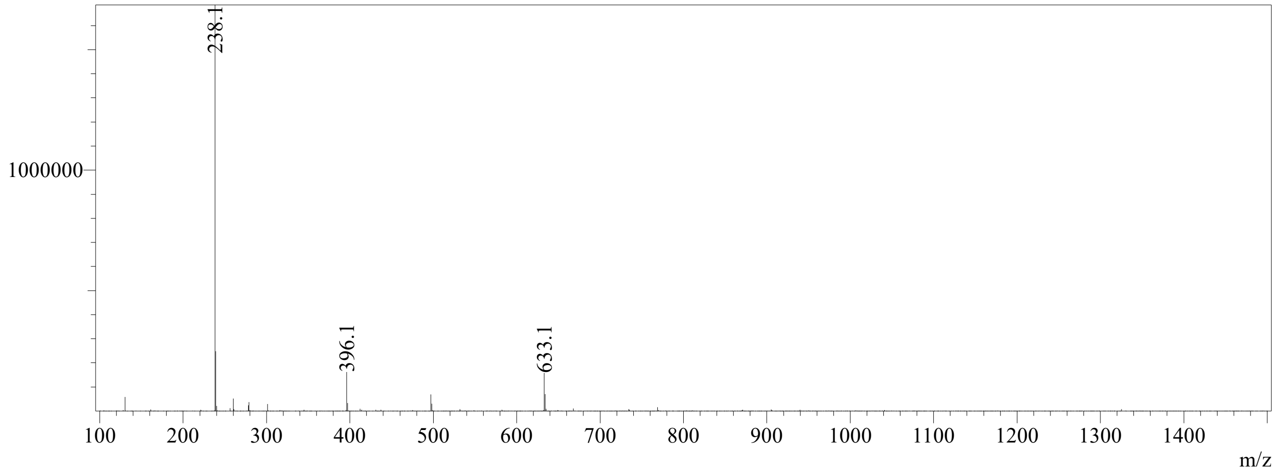

MassPeaks:711
Spectrum Mode:Averaged 0.953-0.980(85-87) Base Peak:130.2(97600)
BG Mode:Calc Segment 1 - Event 1

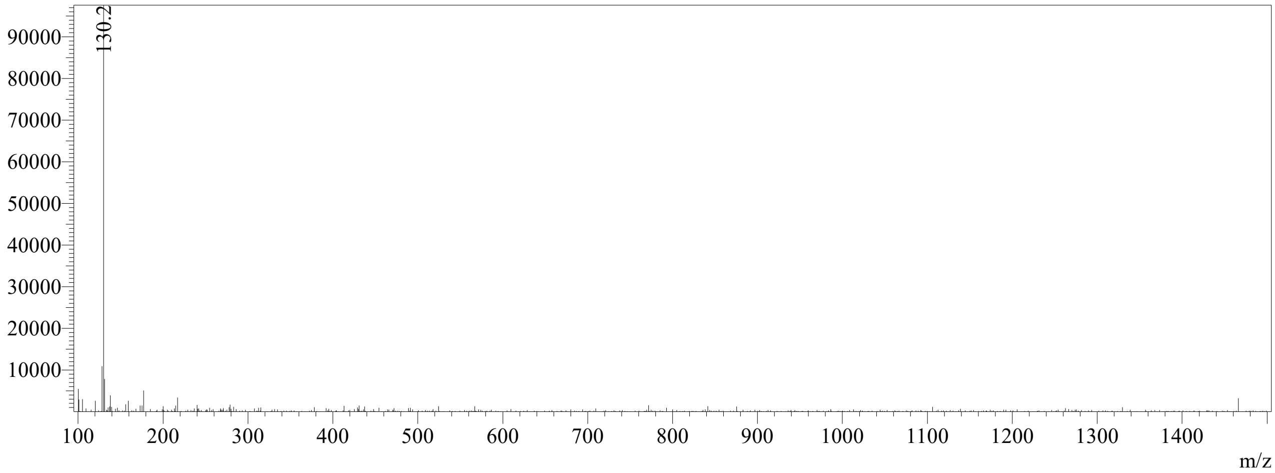

MassPeaks:690
Spectrum Mode:Averaged 2.940-2.967(234-236) Base Peak:130.2(41850)
BG Mode:Calc Segment 1 - Event 1

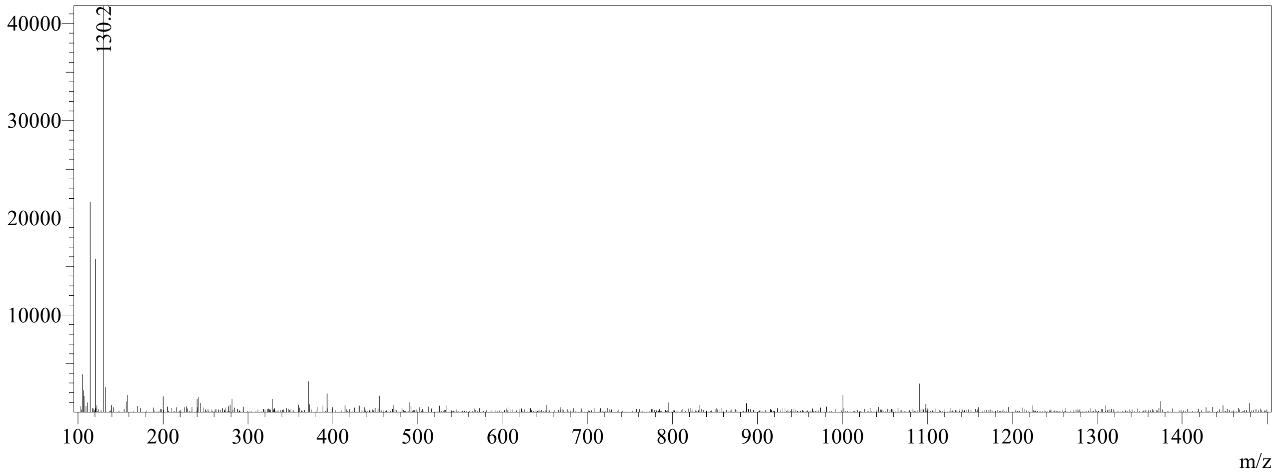

MassPeaks:714
Spectrum Mode:Averaged 3.180-3.207(252-254) Base Peak:130.2(35270)
BG Mode:Calc Segment 1 - Event 1

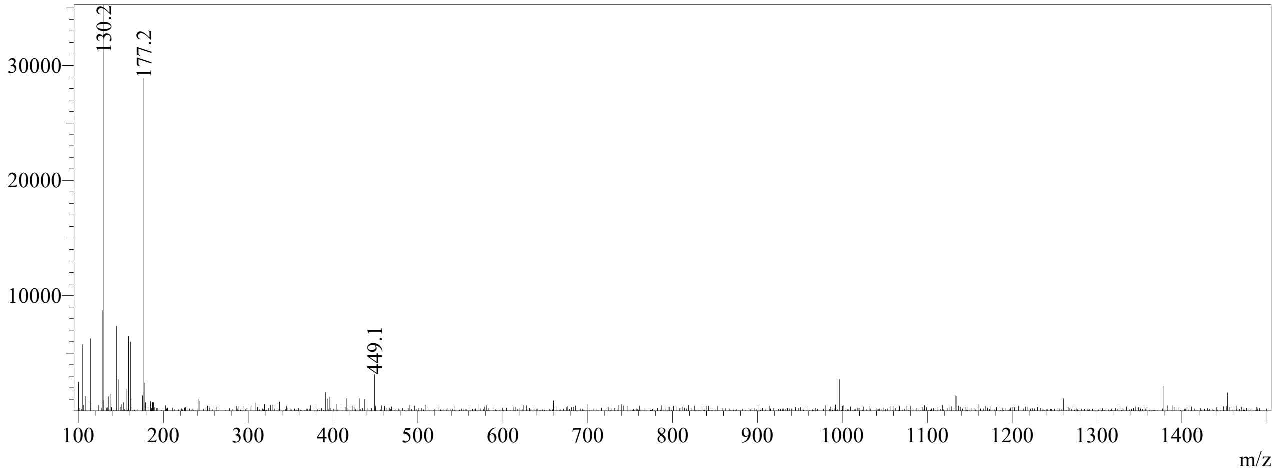

Supplement: Supplementary file 1 [file molecules-31-00522-s001.zip › ESM_F1_Characterization of Compounds in Scheme 1/Compound b_LC-MS.pdf]
