# Supplementary material for: De Novo Generation-Based Design of Potential Computational Hits Targeting the GluN1-GluN2A Receptor
Source: Molecules. 2026 Feb 2;31(3):522. doi: 10.3390/molecules31030522 (PMC12900030; doi:10.3390/molecules31030522)

LC-MS Report

Sample Information

Instrument	: LCMS-01
Sample Name	: Compound c
Sample ID	: LRY
Injection Volume	: 0.5 µL
Vial#	: 42
Method File	: LCMS-LC017-0.04%AB5-95(+)-01.lcm
Date Acquired	: 05/Nov/2025 5:16:02 PM
Modified Date	: 05/Nov/2025 5:19:34 PM

Method
Instrument : Shimadzu LCMS-2020
Column : Shim-pack GIST C18 , 2.1 mm*50 mm , 5.0 um
Oven Temperature : 40 Flow Rate : 1.0000 mL/min
Mobile Phase : A : H2O+0.04%TFA
Mobile Phase : B : ACN+0.02%TFA

Time	Module	Command	Value
0.01	Pumps	B.Conc	5
2.00	Pumps	B.Conc	95
2.50	Pumps	B.Conc	95
2.51	Pumps	B.Conc	5
3.50	Controller	Stop	

Chromatogram

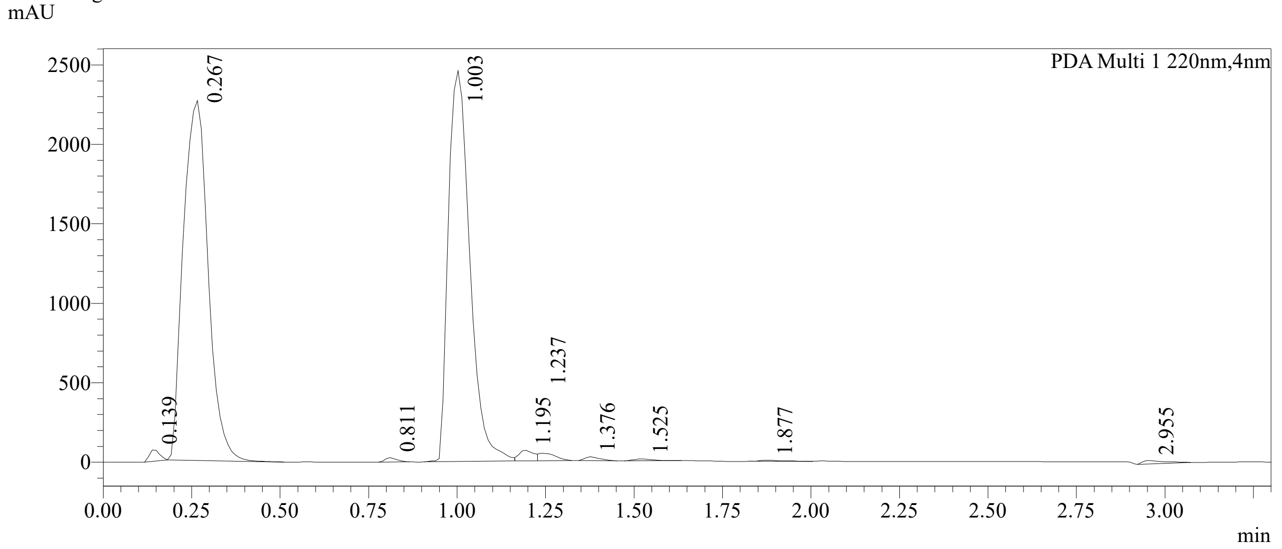

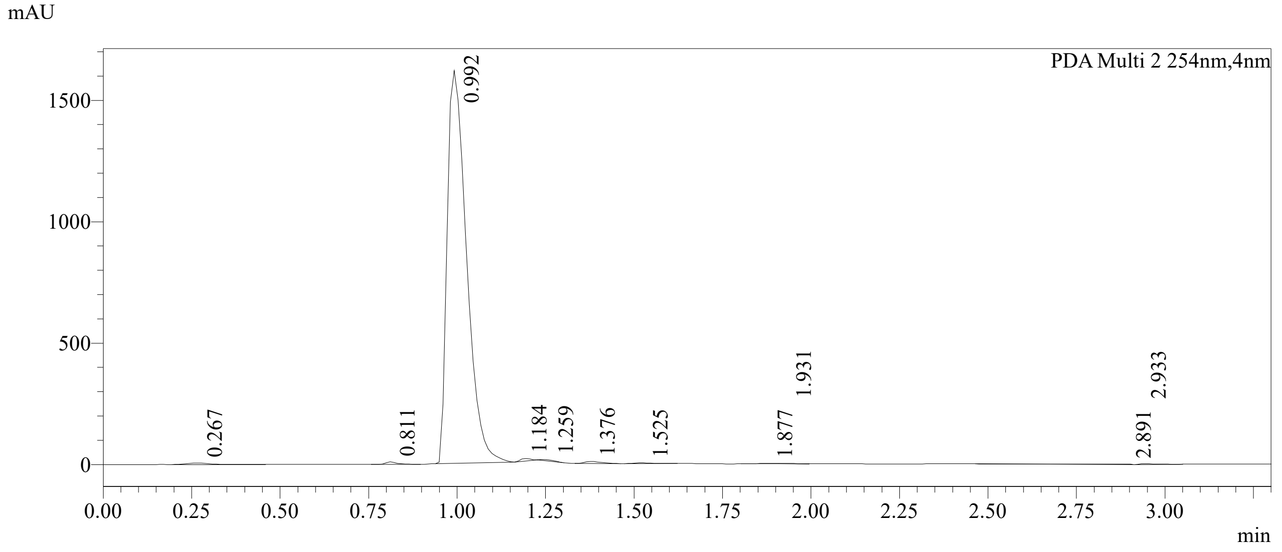

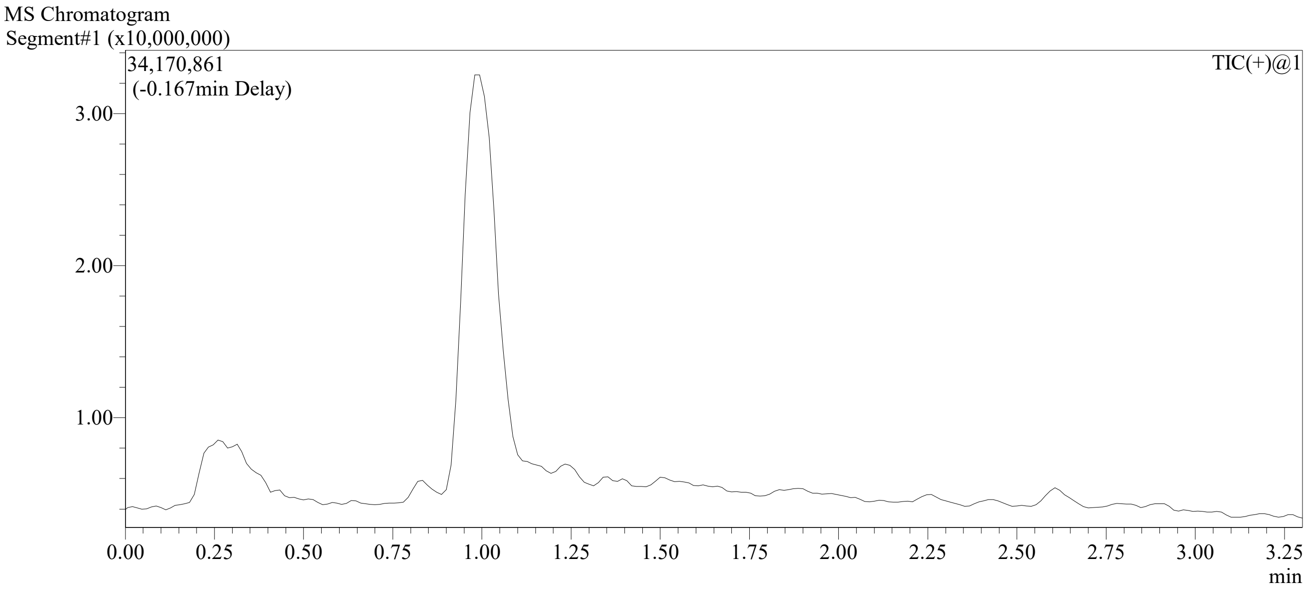

Peak Table
PDA Ch1 220nm

Peak#	Ret. Time	Height	Height%	Area	Area%
1	0.139	73171	1.462	146314	0.655
2	0.267	2266165	45.287	11084636	49.604
3	0.811	27404	0.548	57932	0.259
4	1.003	2460137	49.164	10465794	46.834
5	1.195	66248	1.324	194697	0.871
6	1.237	47043	0.940	158346	0.709
7	1.376	23209	0.464	67390	0.302
8	1.525	11464	0.229	43838	0.196
9	1.877	7422	0.148	36393	0.163
10	2.955	21725	0.434	91000	0.407

PDA Ch2 254nm

Peak#	Ret. Time	Height	Height%	Area	Area%
1	0.267	6980	0.417	37134	0.580
2	0.811	10195	0.610	24373	0.381
3	0.992	1617340	96.692	6200370	96.899
4	1.184	10342	0.618	22014	0.344
5	1.259	5291	0.316	14036	0.219
6	1.376	8686	0.519	28230	0.441
7	1.525	2909	0.174	10996	0.172
8	1.877	2504	0.150	7930	0.124
9	1.931	2306	0.138	7972	0.125
10	2.891	2415	0.144	32170	0.503
11	2.933	3707	0.222	13546	0.212

MS Spectrum

MassPeaks:742
Spectrum Mode:Averaged 0.127-0.153(23-25) Base Peak:130.2(115875)
BG Mode:Calc Segment 1 - Event 1

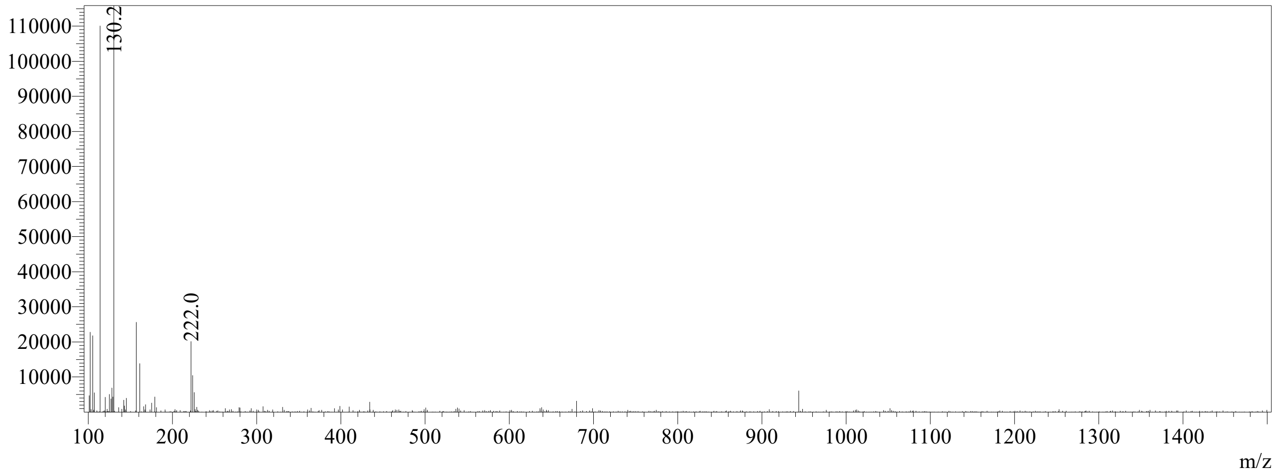

MassPeaks:664
Spectrum Mode:Averaged 0.247-0.273(32-34) Base Peak:147.2(5383387)
BG Mode:Calc Segment 1 - Event 1

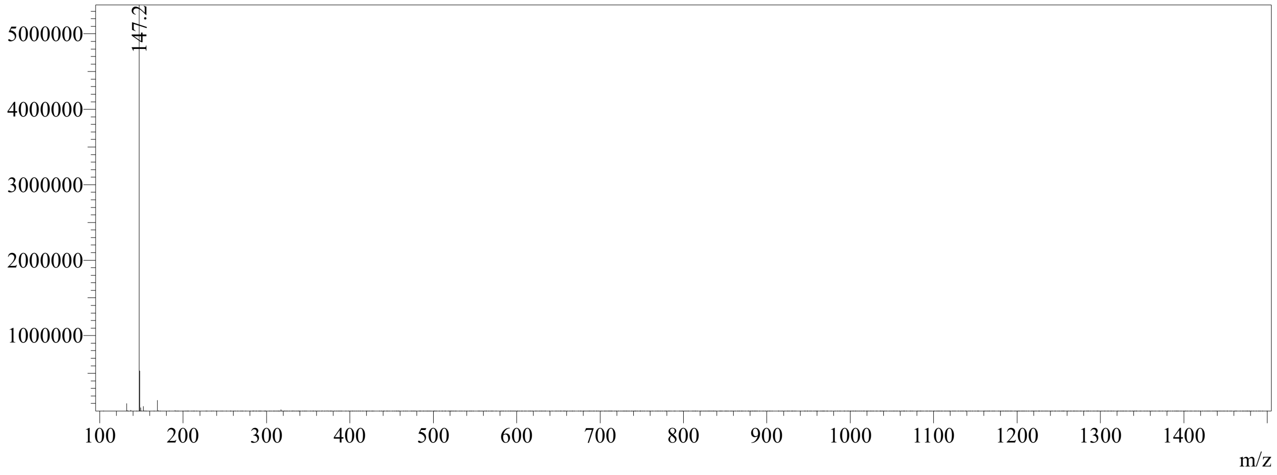

MassPeaks:722
Spectrum Mode:Averaged 0.793-0.820(73-75) Base Peak:218.2(427366)
BG Mode:Calc Segment 1 - Event 1

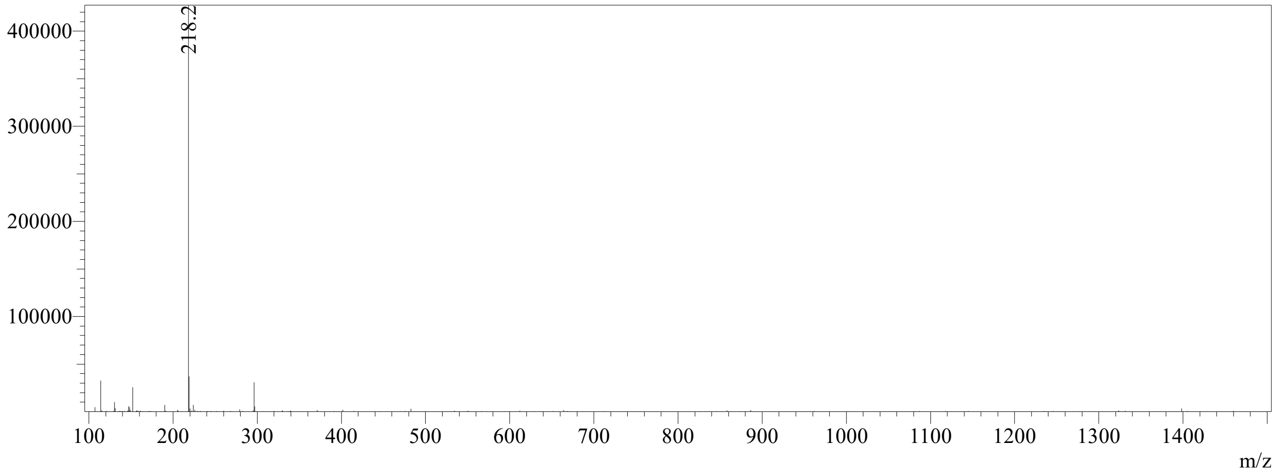

MassPeaks:848
Spectrum Mode:Averaged 0.993-1.020(88-90) Base Peak:324.1(9518358)
BG Mode:Calc Segment 1 - Event 1

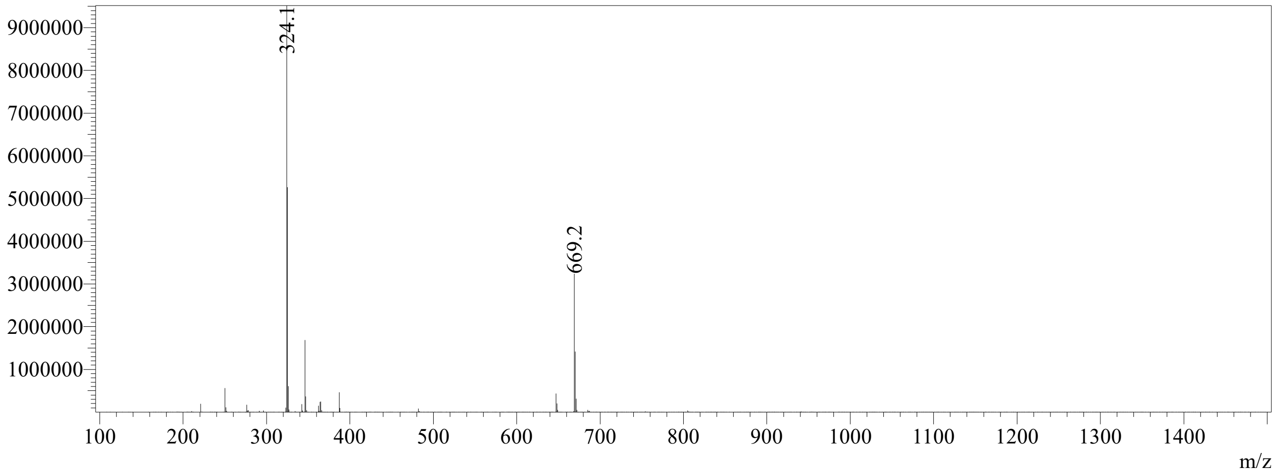

MassPeaks:622
Spectrum Mode:Averaged 1.180-1.207(102-104) Base Peak:128.2(7767)
BG Mode:Calc Segment 1 - Event 1

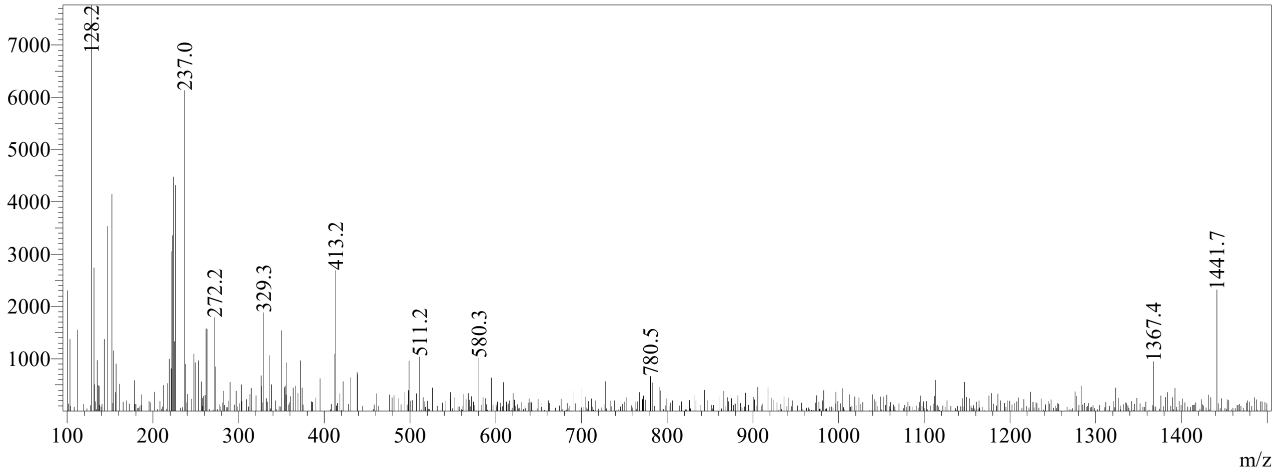

MassPeaks:700
Spectrum Mode:Averaged 1.220-1.247(105-107) Base Peak:482.2(90393)
BG Mode:Calc Segment 1 - Event 1

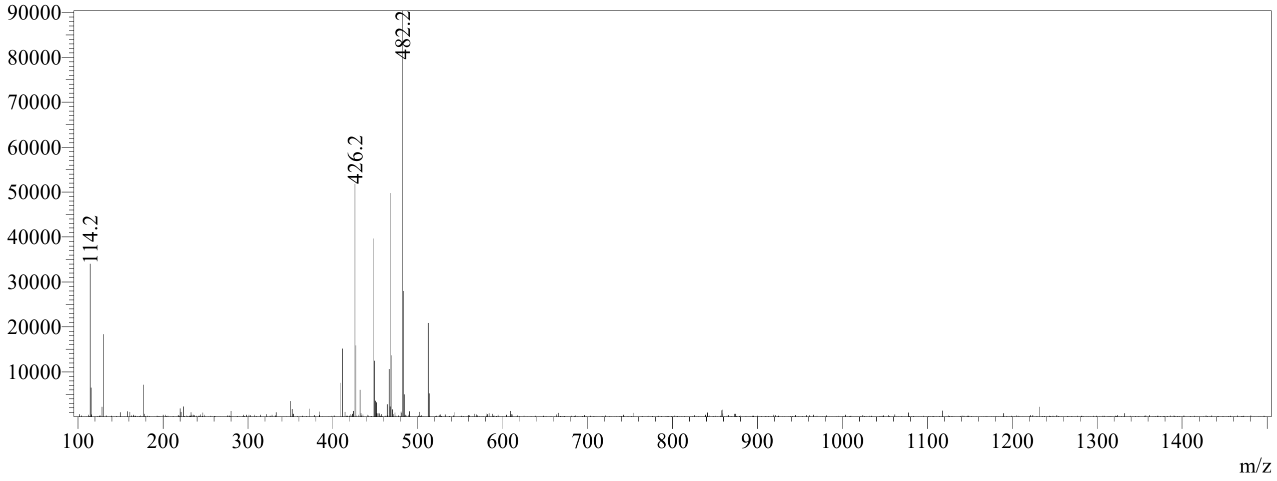

MassPeaks:656
Spectrum Mode:Averaged 1.367-1.393(116-118) Base Peak:554.2(67519)
BG Mode:Calc Segment 1 - Event 1

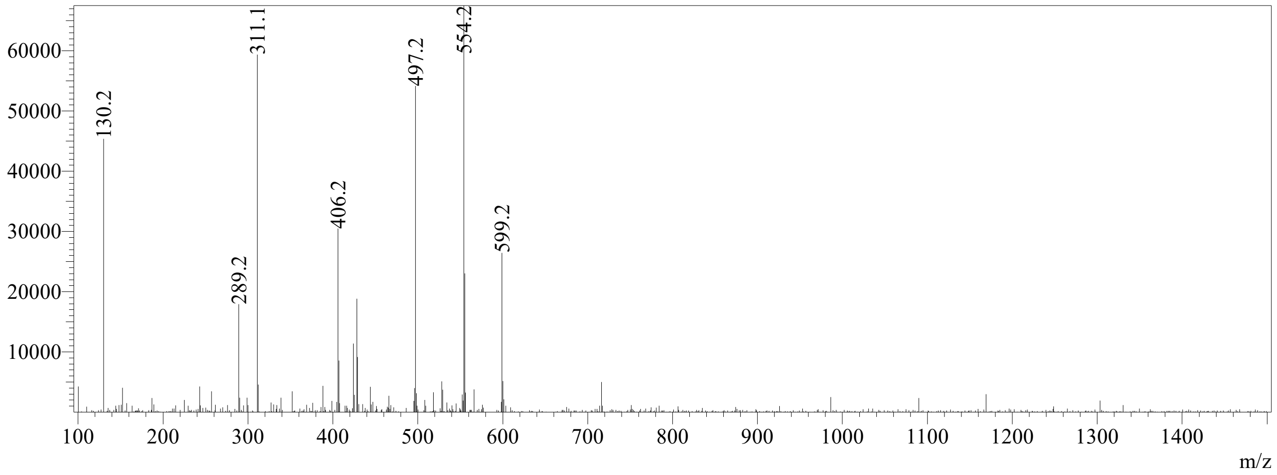

MassPeaks:674
Spectrum Mode:Averaged 1.513-1.540(127-129) Base Peak:640.2(178646)
BG Mode:Calc Segment 1 - Event 1

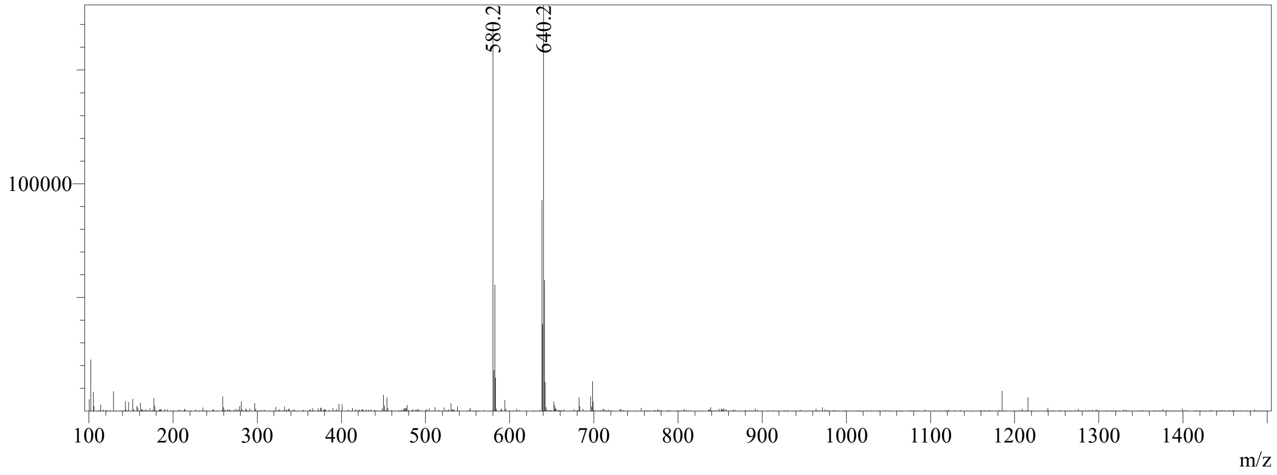

MassPeaks:701
Spectrum Mode:Averaged 1.860-1.887(153-155) Base Peak:115.2(54725)
BG Mode:Calc Segment 1 - Event 1

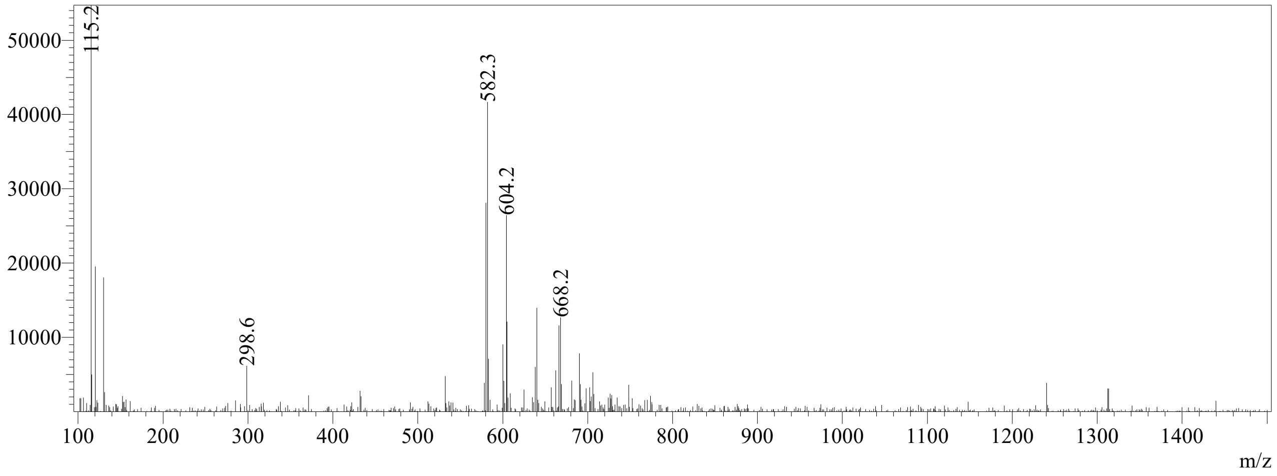

MassPeaks:759
Spectrum Mode:Averaged 2.873-2.900(229-231) Base Peak:100.3(23426)
BG Mode:Calc Segment 1 - Event 1

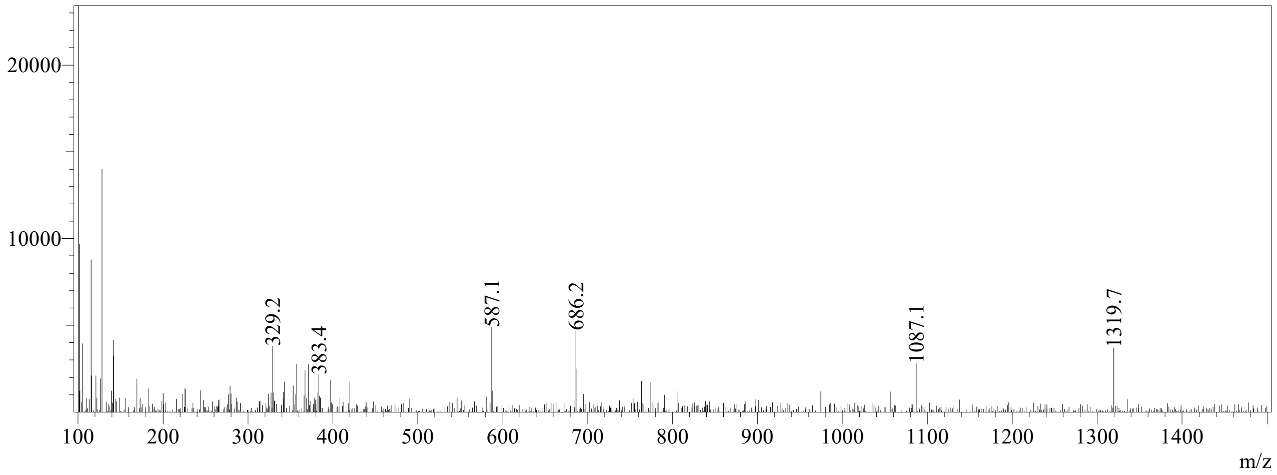

MassPeaks:644
Spectrum Mode:Averaged 2.940-2.967(234-236) Base Peak:1438.4(10774)
BG Mode:Calc Segment 1 - Event 1

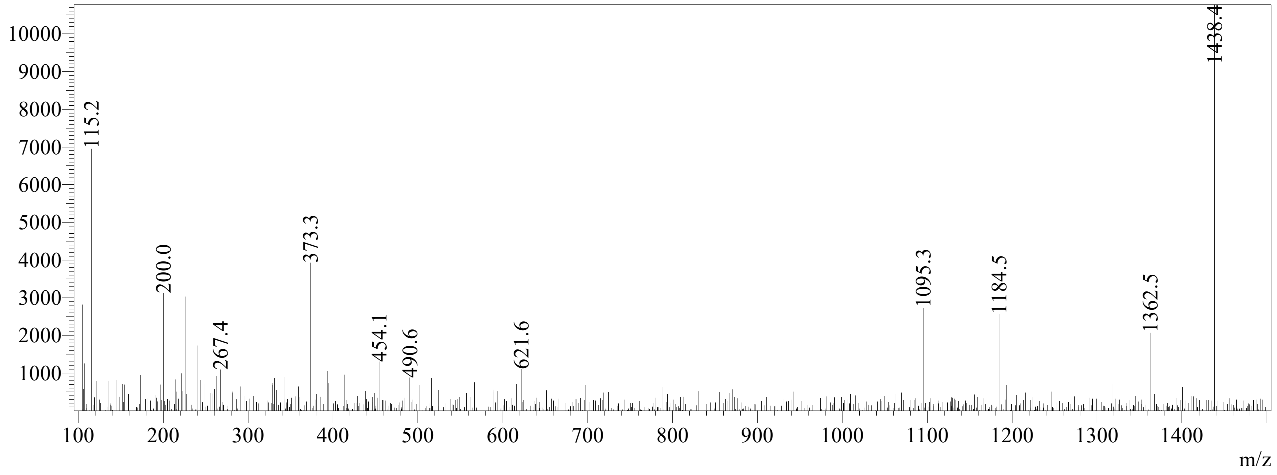

Supplement: Supplementary file 1 [file molecules-31-00522-s001.zip › ESM_F1_Characterization of Compounds in Scheme 1/Compound c_LC-MS.pdf]
