# Supplementary material for: De Novo Generation-Based Design of Potential Computational Hits Targeting the GluN1-GluN2A Receptor
Source: Molecules. 2026 Feb 2;31(3):522. doi: 10.3390/molecules31030522 (PMC12900030; doi:10.3390/molecules31030522)

LC-MS Report

Sample Name : A2
Vial# : 91
Injection Volume : 50
Data File : Z:\data\Data\LCMS006\2025\202511\251106\A2_LCMS006_1206_001.lcd
Method File : D\Method-2\normal-1.0.lcm
Date Acquired : 10/24/2025 12:08:18 PM

Chromatogram

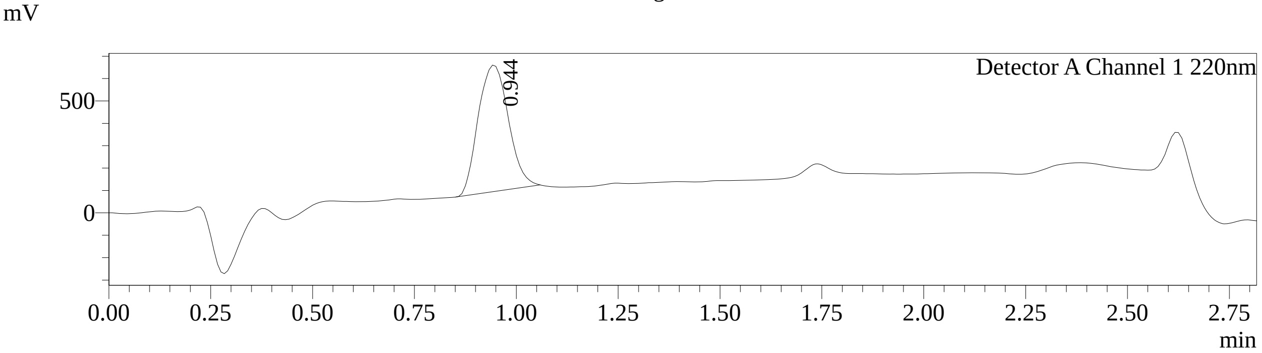

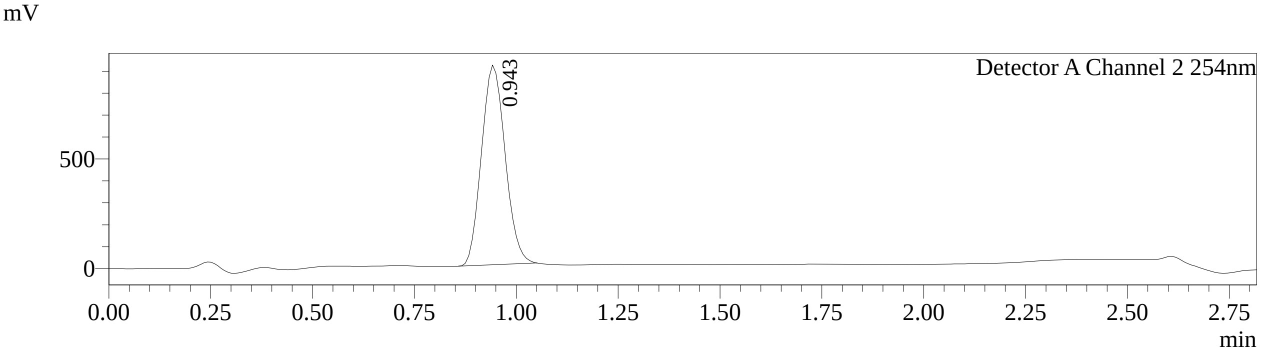

MS Spectrum Graph

Line#:1 R.Time:0.967(Scan#:59)
MassPeaks:909
Spectrum Mode:Single 0.967(59) BasePeak:329.30(908805)
BG Mode:None Segment 1 - Event 1
ESI Positive

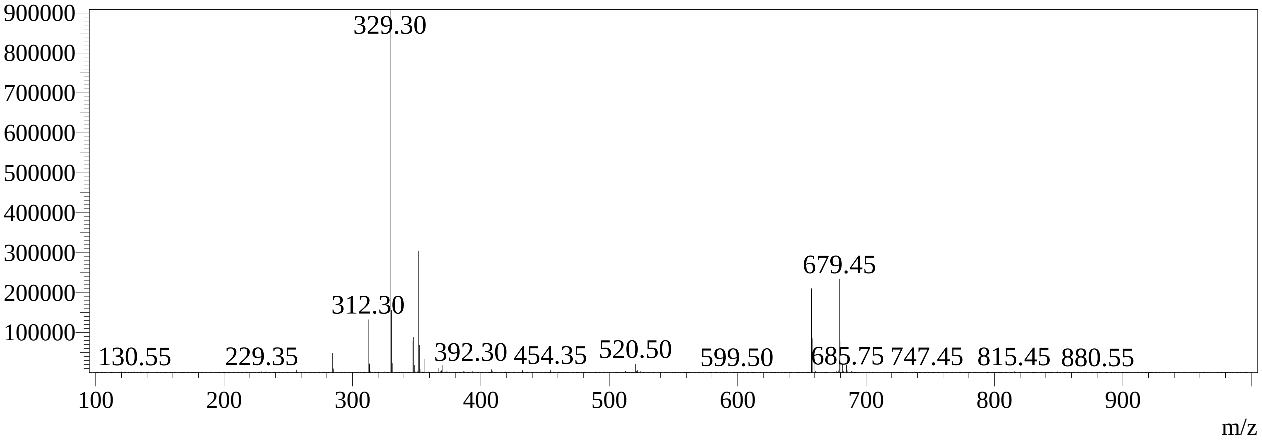

Line#:2   R.Time:0.983(Scan#:60)
MassPeaks:905
Spectrum Mode:Single 0.983(60) BasePeak:249.00(37273)
BG Mode:None Segment 1 - Event 2
ESI Negative

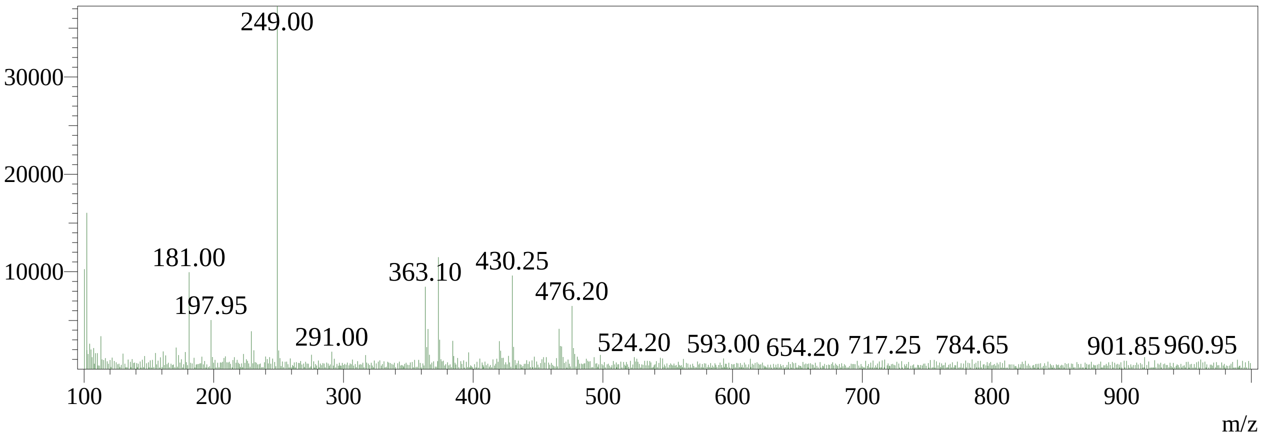

Supplement: Supplementary file 1 [file molecules-31-00522-s001.zip › ESM_F2_Characterization of Compounds in Scheme 2/A2_LC-MS.pdf]
