# Supplementary material for: De Novo Generation-Based Design of Potential Computational Hits Targeting the GluN1-GluN2A Receptor
Source: Molecules. 2026 Feb 2;31(3):522. doi: 10.3390/molecules31030522 (PMC12900030; doi:10.3390/molecules31030522)
Supplement: Supplementary file 1 [file molecules-31-00522-s001.zip › ESM_F2_Characterization of Compounds in Scheme 2/A2_OR.pdf]

Optical Rotation Report

Measurement Date :01/12/2026

Method Name : GTM-11SR-20

Sample ID : A2

Compound ID : A2

Solvent : MeOH

Set Temperature : 20.0°C

N	Avg.	Std.Dev.	%RSD	Min	Max
3	-19.47	0.12	-0.61	-19.60	-19.40

S.No	Result	Scale	OR °Arc	WLG.nm	Lg.mm	Conc.g/100mL	Temp
1	-19.40	SR	-0.097	589	50	1.0000	20.0°C
2	-19.40	SR	-0.097	589	50	1.0000	19.9°C
3	-19.60	SR	-0.098	589	50	1.0000	19.9°C

Operator Comment : Result is the specific optical rotation.
