# Supplementary material for: De Novo Generation-Based Design of Potential Computational Hits Targeting the GluN1-GluN2A Receptor
Source: Molecules. 2026 Feb 2;31(3):522. doi: 10.3390/molecules31030522 (PMC12900030; doi:10.3390/molecules31030522)
Supplement: Supplementary file 1 [file molecules-31-00522-s001.zip › ESM_F2_Characterization of Compounds in Scheme 2/A2_SFC.pdf]

Chiral SFC Report

Sample Information

Sample ID:	A2
Compound ID:	A2
Date Acquired:	1/12/2026 10:20:12 PM CST
Date Processed:	1/13/2026 10:24:11 AM CST
Injection Volume:	1.50 ul
Vial:	1:B,4
Acq Method :	OD_EtOH_MNH3_5_50_34_35_3min
Raw Data:	D:\Data\ID_m32_result_ 29808
Project Name:	2026\CASTJ_CA\SFC-P-20260104
Instrument:	CAS-02-ANA-SFC-P(Waters UPCC with QDA)
Label:	No Racemate

Test Results

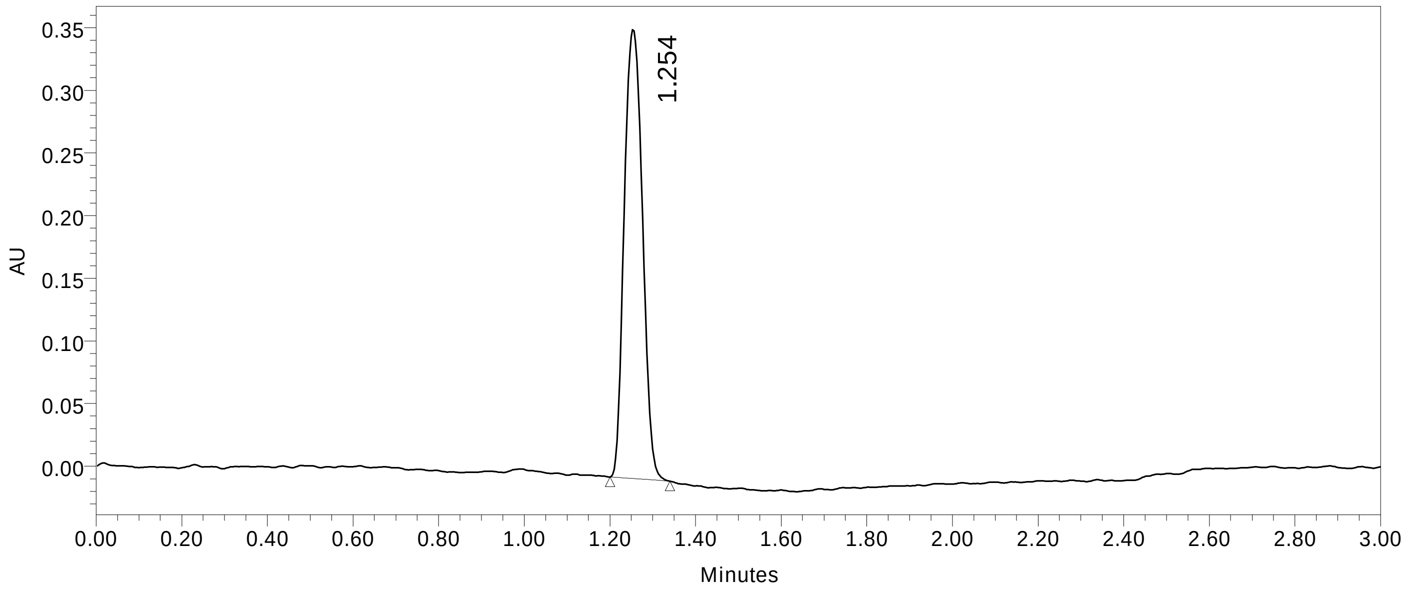

Channel: PDA Spectrum PDA 220.0 nm (PDA Spectrum (190-300)nm)

	RT	Width	Height (mAU)	Resolution	Symmetry	Area	% Area(*)
1	1.254	0.140	358.724	NA	1.1	1064.757	100.00

Method Information

Instrument Method: OD_EtOH_MNH3_5_50_34_35_3min

Stored: 1/4/2026 1:20:57 PM CST

Method Information

Method Comments	Column: Chiralcel OD-3, 50×4.6mm I.D., 3um<br>Mobile phase: A:CO2 B:EtOH[0.2%NH3(7MinMeOH)]<br>Gradient:<br>Time A% B%<br>0.0 95 5<br>0.2 95 5<br>1.2 50 50<br>2.2 50 50<br>2.6 95 5<br>3.0 95 5<br>Flow rate: 3.4mL/min<br>Column temp.: 35°C<br>ABPR: 1800psi
Method Modified User	CASTJ_CA
Method Locked	No
Method Id	4662
Old Id	
Method Version	2
Method Edit User	
Source S/W Info	Empower 3 Software Build 3471 SPs Installed: Service Release 3 DB ID: 2926695483
