# Supplementary material for: De Novo Generation-Based Design of Potential Computational Hits Targeting the GluN1-GluN2A Receptor
Source: Molecules. 2026 Feb 2;31(3):522. doi: 10.3390/molecules31030522 (PMC12900030; doi:10.3390/molecules31030522)
Supplement: Supplementary file 1 [file molecules-31-00522-s001.zip › ESM_F2_Characterization of Compounds in Scheme 2/Compound d_HPLC.pdf]

HPLC REPORT

Compound ID	: Compound d
Sample ID	: Compound d
Injection Date	:2026/1/13 16:43:46
Injection Vol	: 1ul
Location	:tray1 vail20
Acq Method	:D:\SYSTEM\method\10_80CD_6min.lcm
Org DataFile	:D:\DATA\2026\2601\260113\Compound d
Instrument	:02-HPLC-072

Chromatogram

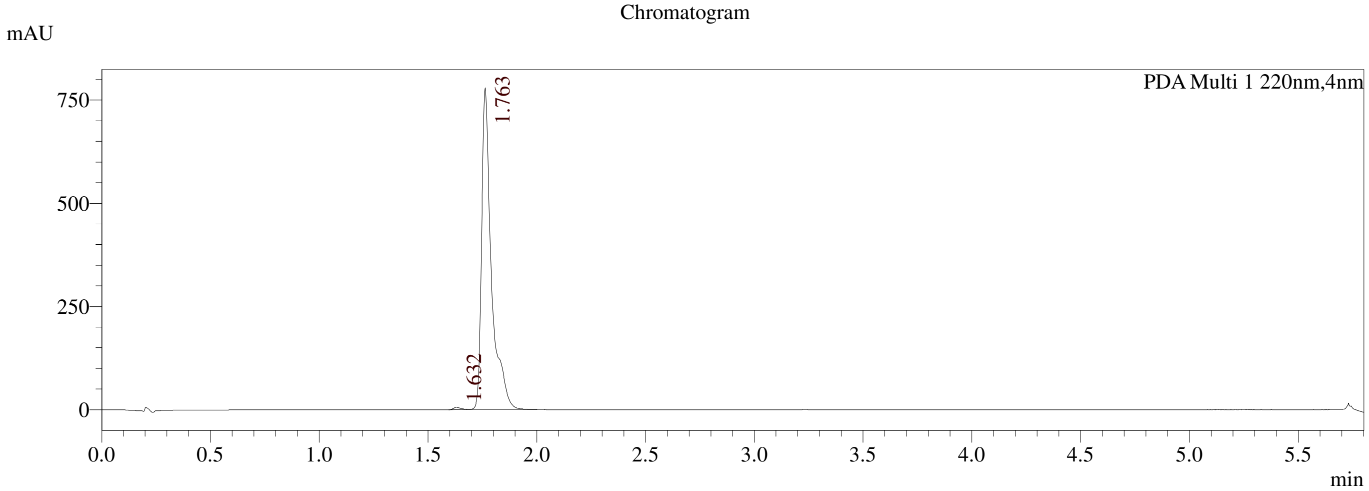

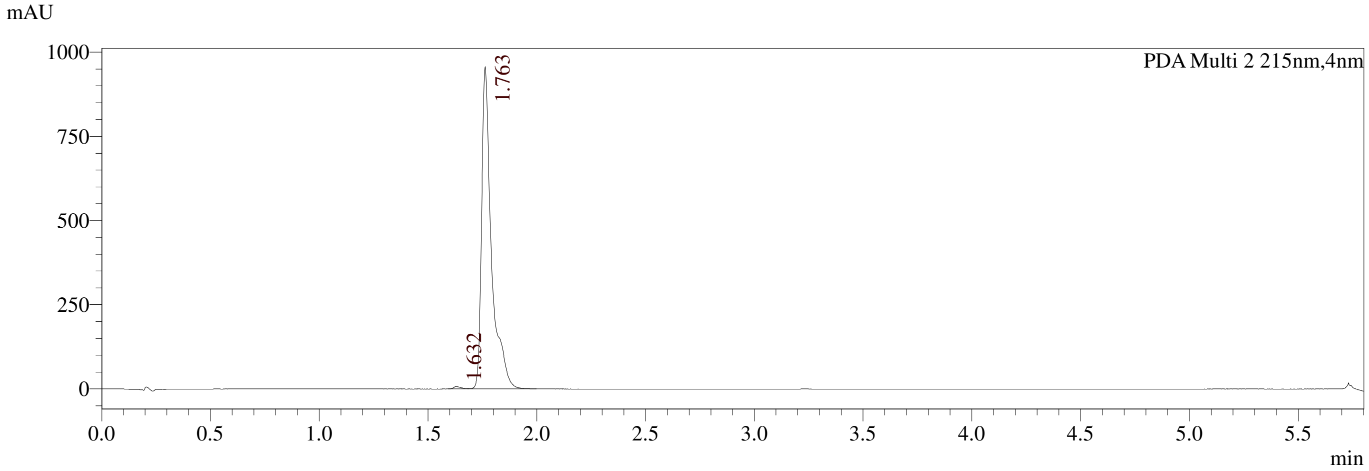

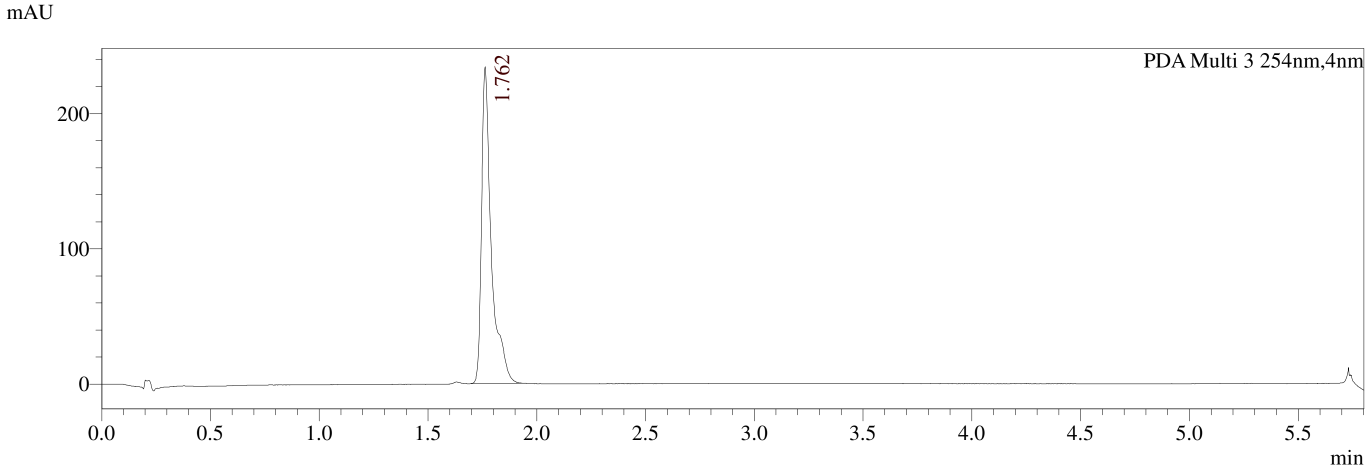

1 PDA Multi 1 / 220nm,4nm
2 PDA Multi 2 / 215nm,4nm
3 PDA Multi 3 / 254nm,4nm

==
Integration Result
==

PDA Ch1 220nm

Peak#	Ret. Time	Width	Height	Height%	Area	Area%
1	1.632	0.063	5565	0.710	13217	0.534
2	1.763	0.072	778320	99.290	2461948	99.466

PDA Ch2 215nm

Peak#	Ret. Time	Width	Height	Height%	Area	Area%
1	1.632	0.067	6989	0.726	16984	0.558
2	1.763	0.072	955753	99.274	3026202	99.442

PDA Ch3 254nm

Peak#	Ret. Time	Width	Height	Height%	Area	Area%
1	1.762	0.071	233716	100.000	733885	100.000
