# Supplementary material for: De Novo Generation-Based Design of Potential Computational Hits Targeting the GluN1-GluN2A Receptor
Source: Molecules. 2026 Feb 2;31(3):522. doi: 10.3390/molecules31030522 (PMC12900030; doi:10.3390/molecules31030522)
Supplement: Supplementary file 1 [file molecules-31-00522-s001.zip › ESM_F2_Characterization of Compounds in Scheme 2/Compound d_LC-MS.pdf]

LC-MS REPORT

Compound ID : Compound d
Sample ID : Compound d
Injection Date : 13. Jan. 2026
Location : P1-B-06
Inj. Vol. : 1.00 uL
Acq Method : D:\DATA\2601\260113 18\5_95CD_6min_220&254
Data Filename : D:\DATA\2601\260113 18\Compound d.D
Instrument : 02-LCMS-U

Chromatogram

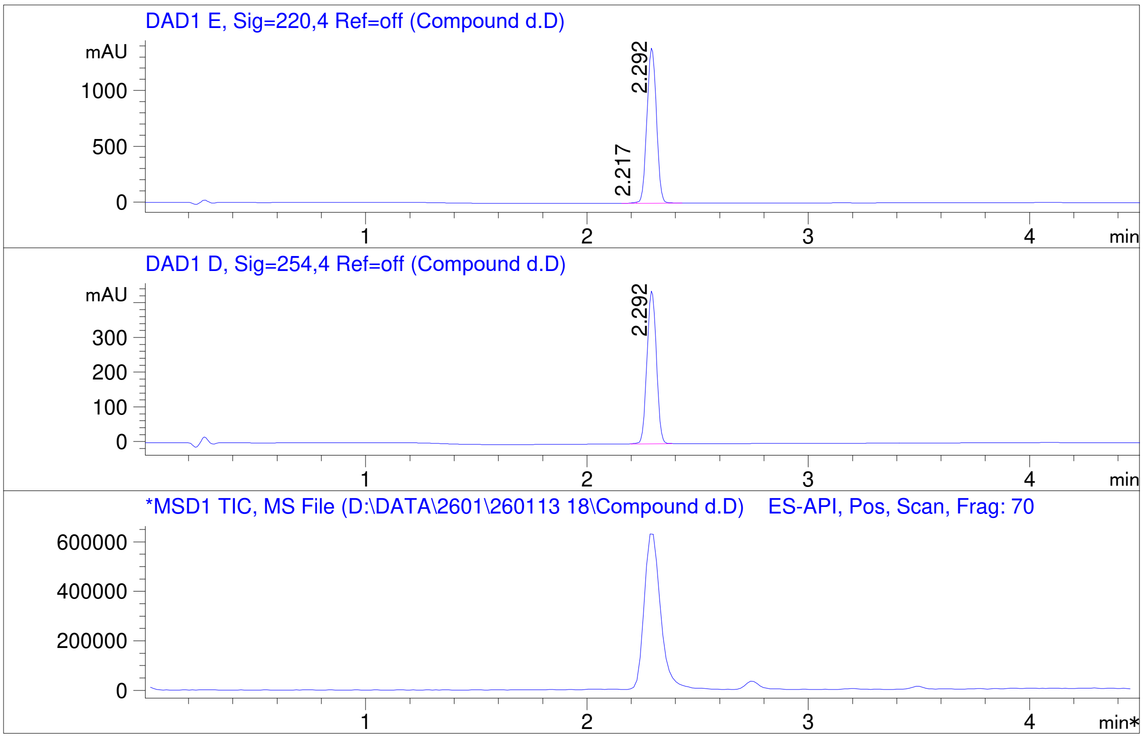

Integration Result

Signal 1 : DAD1 E, Sig=220,4 Ref=off

Peak #	RT [min]	Height	Height %	Width [min]	Area	Area %
1	2.217	7.986	0.569	0.021	9.866	0.238
2	2.292	1395.052	99.431	0.047	4132.598	99.762

Signal 2 : DAD1 D, Sig=254,4 Ref=off

Peak #	RT [min]	Height	Height %	Width [min]	Area	Area %
1	2.292	441.849	100.000	0.046	1304.189	100.000

Signal 3 : MSD1 TIC, MS File

Peak #	RT [min]	Height	Height %	Width [min]	Area	Area %

MS Spectrum

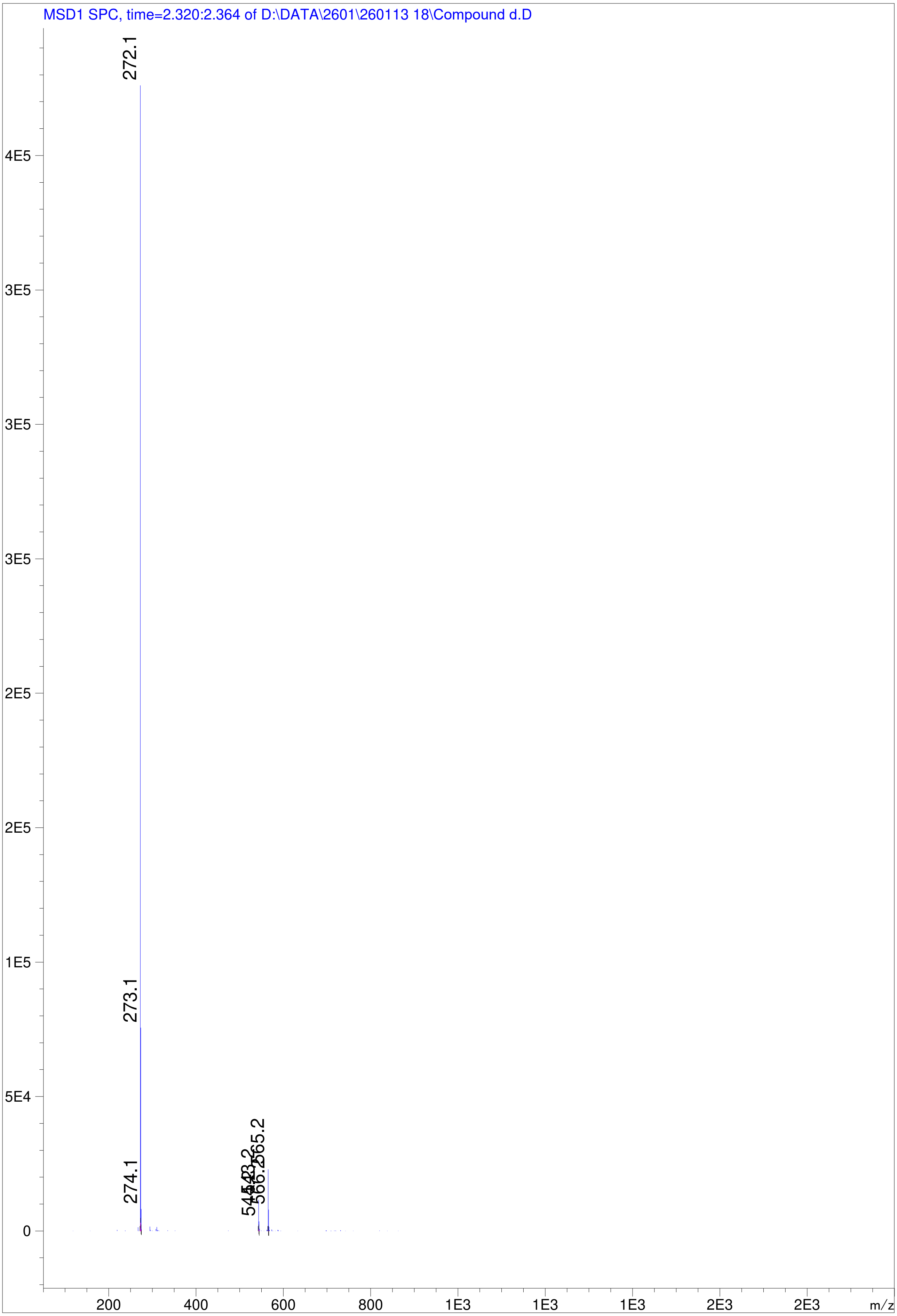

MSD1 SPC, time=2.320:2.364 of D:\DATA\2601\260113 18\Compound d.D
272.1
273.1
274.1
565.2
4E5
3E5
3E5
3E5
2E5
2E5
1E5
5E4
0
200
400
600
800
1E3
1E3
1E3
2E3
2E3
m/z

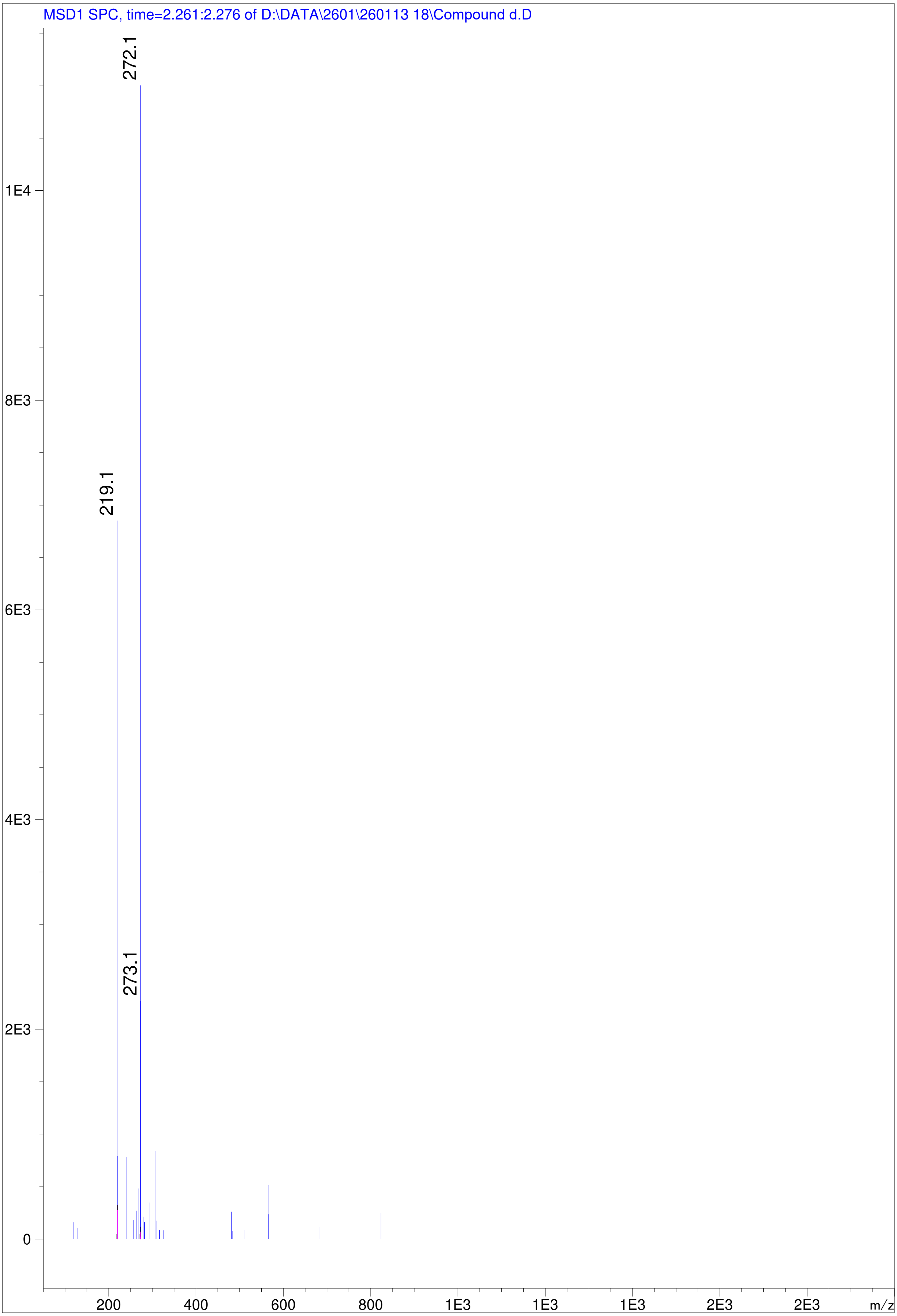
MSD1 SPC, time=2.261:2.276 of D:\DATA\2601\260113 18\Compound d.D
272.1
219.1
273.1
1E4
8E3
6E3
4E3
2E3
0
200
400
600
800
1E3
1E3
1E3
2E3
2E3
m/z
