# Supplementary material for: De Novo Generation-Based Design of Potential Computational Hits Targeting the GluN1-GluN2A Receptor
Source: Molecules. 2026 Feb 2;31(3):522. doi: 10.3390/molecules31030522 (PMC12900030; doi:10.3390/molecules31030522)
Supplement: Supplementary file 1 [file molecules-31-00522-s001.zip › ESM_F2_Characterization of Compounds in Scheme 2/Compound d_OR.pdf]

Optical Rotation Report

Measurement Date : 01/15/2026

Method Name : GTM-11-SR-20

Sample ID : Compound d

Compound ID : Compound d

Solvent : MeOH

Set Temperature : 20.0°C

N	Avg.	Std.Dev.	%RSD	Min	Max
3	-33.83	0.00	0.00	-33.83	-33.83

S.No	Result	Scale	OR °Arc	WLG.nm	Lg.mm	Conc.g/100mL	Temp
1	-33.83	SR	-0.181	589	50	1.0700	20.1°C
2	-33.83	SR	-0.181	589	50	1.0700	20.1°C
3	-33.83	SR	-0.181	589	50	1.0700	20.1°C

Operator Comment : Result is the specific optical rotation.
