# Supplementary material for: De Novo Generation-Based Design of Potential Computational Hits Targeting the GluN1-GluN2A Receptor
Source: Molecules. 2026 Feb 2;31(3):522. doi: 10.3390/molecules31030522 (PMC12900030; doi:10.3390/molecules31030522)
Supplement: Supplementary file 1 [file molecules-31-00522-s001.zip › ESM_F3_Characterization of Compounds in Scheme 3/A3_HPLC.pdf]

Data File Z:\HPLC-10(78)\2025\202510\251031\A3.D

Sample Name: A3

===

```
Acq. Operator   :                               Seq. Line :   1
Acq. Instrument : HPLC078                        Location : Vial 25
Injection Date  : 10/31/2025 1:59:07 PM               Inj :   1
                                               Inj Volume : 5.0 µl
Acq. Method     : C:\CHEMSTATION\2\METHODS\5-95.M
```

DAD1 A, Sig=254,16 Ref=off (Z:\HPLC-10(78)\2025\202510\251031\A3.D)

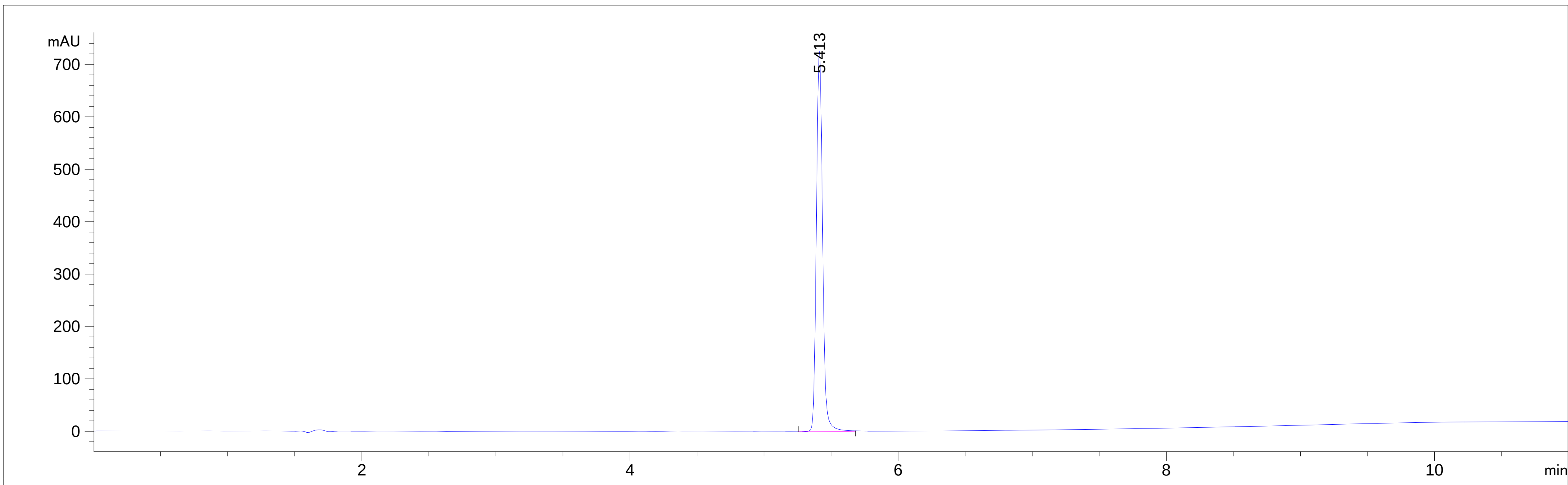

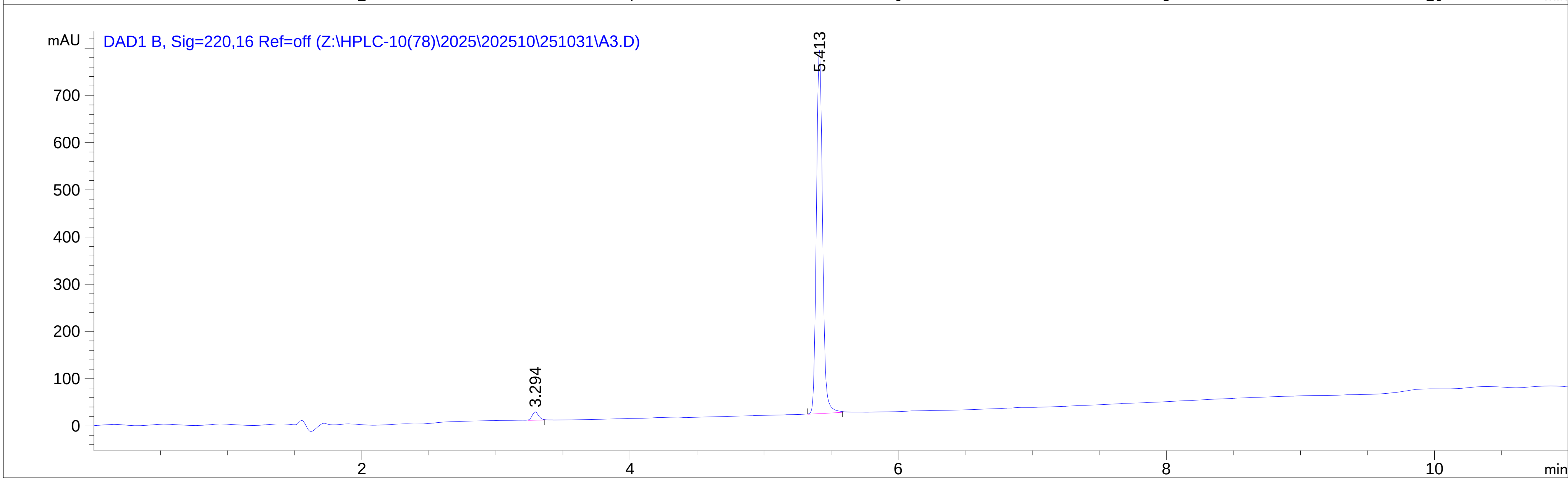

===

Area Percent Report

===

Sorted By : Signal

Multiplier: : 1.0000

Dilution: : 1.0000

Use Multiplier & Dilution Factor with ISTDs

Signal 1: DAD1 A, Sig=254,16 Ref=off

Peak #	RetTime [min]	Type	Width [min]	Area [mAU*s]	Height [mAU]	Area %
1	5.413	BB	0.0497	2354.24316	728.97852	100.0000

Totals : 2354.24316 728.97852

Signal 2: DAD1 B, Sig=220,16 Ref=off

Peak #	RetTime [min]	Type	Width [min]	Area [mAU*s]	Height [mAU]	Area %
1	3.294	MM R	0.0567	59.52544	17.50861	2.3474
2	5.413	MM R	0.0531	2476.25195	776.53137	97.6526

Totals : 2535.77739 794.03998

===

*** End of Report ***
