# Supplementary material for: De Novo Generation-Based Design of Potential Computational Hits Targeting the GluN1-GluN2A Receptor
Source: Molecules. 2026 Feb 2;31(3):522. doi: 10.3390/molecules31030522 (PMC12900030; doi:10.3390/molecules31030522)

LC-MS Report

Sample Name : A3
Vial# : 28
Injection Volume : 10
Data File : Z:\data\Data\LCMS007\2025\202510\251031\A3_Tutorial_Batch_1336_1.
lcd Method Fil : D\7#lcms\method-2\normal-1.0.lcm
Date Acquired : 10/31/2025 2:02:20 PM

Chromatogram

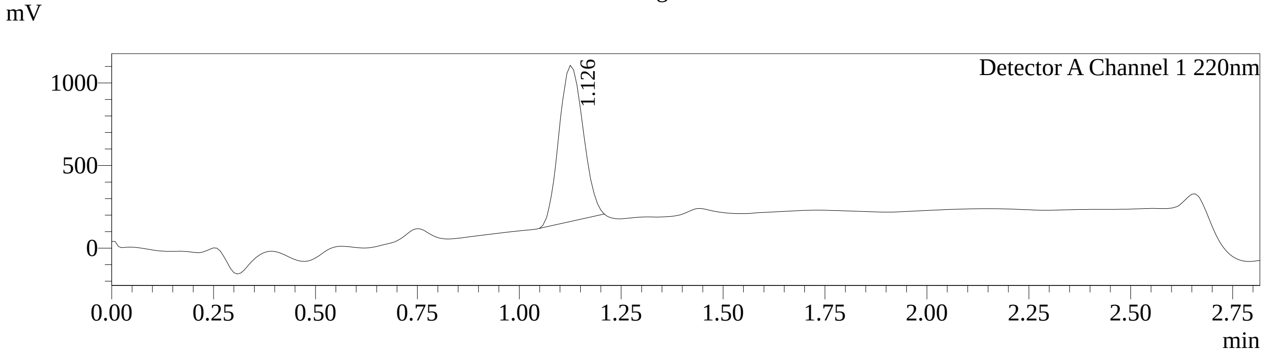

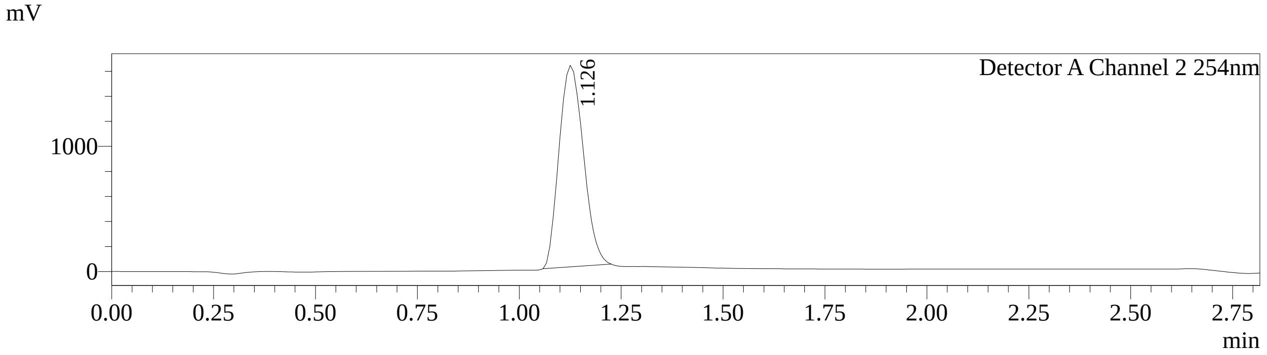

MS Spectrum Graph

Line#:1 R.Time:1.133(Scan#:69)
MassPeaks:910
Spectrum Mode:Single 1.133(69) BasePeak:642.85(5681051)
BG Mode:None Segment 1 - Event 1
ESI Positive

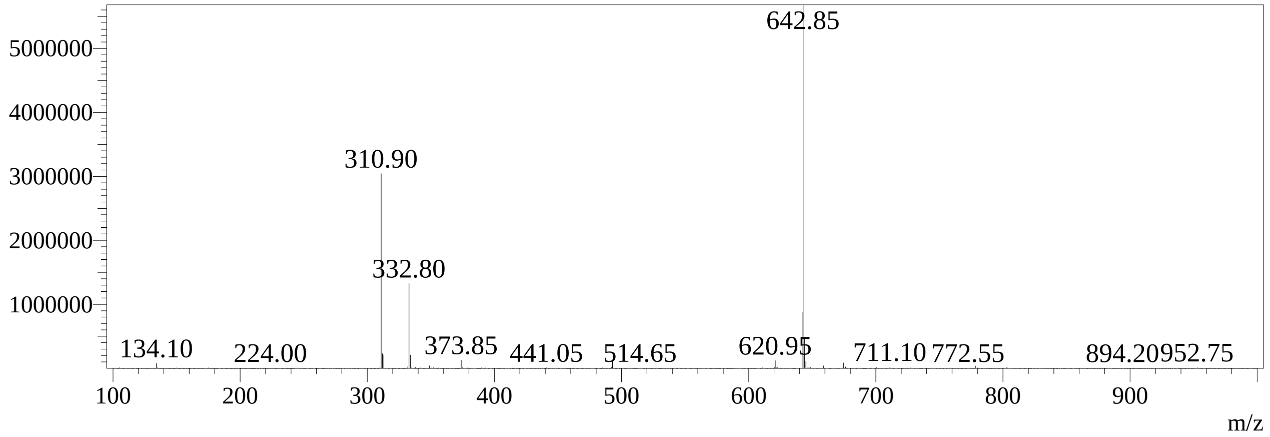

Line#:2   R.Time:1.150(Scan#:70)
MassPeaks:930
Spectrum Mode:Single 1.150(70) BasePeak:248.75(48350)
BG Mode:None Segment 1 - Event 2
ESI Negative

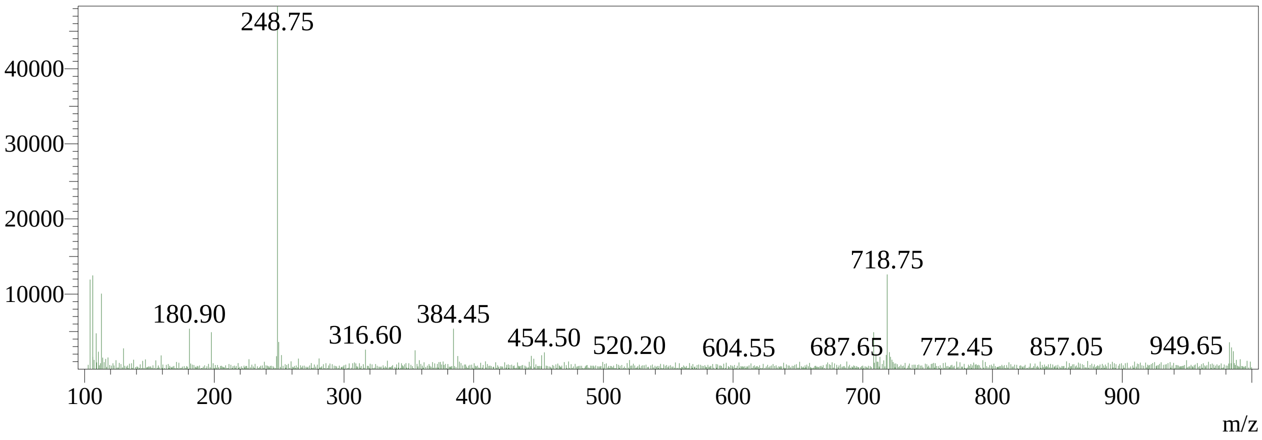

Supplement: Supplementary file 1 [file molecules-31-00522-s001.zip › ESM_F3_Characterization of Compounds in Scheme 3/A3_LC-MS.pdf]
