# Supplementary material for: De Novo Generation-Based Design of Potential Computational Hits Targeting the GluN1-GluN2A Receptor
Source: Molecules. 2026 Feb 2;31(3):522. doi: 10.3390/molecules31030522 (PMC12900030; doi:10.3390/molecules31030522)
Supplement: Supplementary file 1 [file molecules-31-00522-s001.zip › ESM_F3_Characterization of Compounds in Scheme 3/A3_OR.pdf]

Optical Rotation Report

Measurement Date : 01/22/2026

Method Name : GTM-11-SR-20

Sample ID : A3

Compound ID : A3

Solvent : $CHCl_3$

Set Temperature : 20.0°C

N	Avg.	Std.Dev.	%RSD	Min	Max
3	45.93	1.10	2.39	44.80	47.00

S.No	Result	Scale	OR °Arc	WLG.nm	Lg.mm	Conc.g/100mL	Temp
1	44.80	SR	0.224	589	50	1.0000	20.0°C
2	46.00	SR	0.230	589	50	1.0000	20.0°C
3	47.00	SR	0.235	589	50	1.0000	20.0°C

Operator Comment : Result is the specific optical rotation.
