# Supplementary material for: De Novo Generation-Based Design of Potential Computational Hits Targeting the GluN1-GluN2A Receptor
Source: Molecules. 2026 Feb 2;31(3):522. doi: 10.3390/molecules31030522 (PMC12900030; doi:10.3390/molecules31030522)
Supplement: Supplementary file 1 [file molecules-31-00522-s001.zip › ESM_F3_Characterization of Compounds in Scheme 3/A3_SFC.pdf]

Chiral SFC Report

Sample Information

Sample ID:	ET105094-41P1J3_IH_EtOH
Compound ID:	A3
Date Acquired:	1/22/2026 3:52:16 PM CST
Date Processed:	1/22/2026 3:46:04 PM CST
Injection Volume:	5.50 ul
Vial:	2:E,3
Acq Method :	IH_EtOH_MNH3_10_50_34_35_4min
Raw Data:	D:\Data\ID_m32_result_ 37438
Project Name:	2026\CASTJ_CA\SFC-Q-20260104
Instrument:	CAS-02-ANA-SFC-Q(Waters UPCC with PDA)
Label:	AssayEE

Test Results

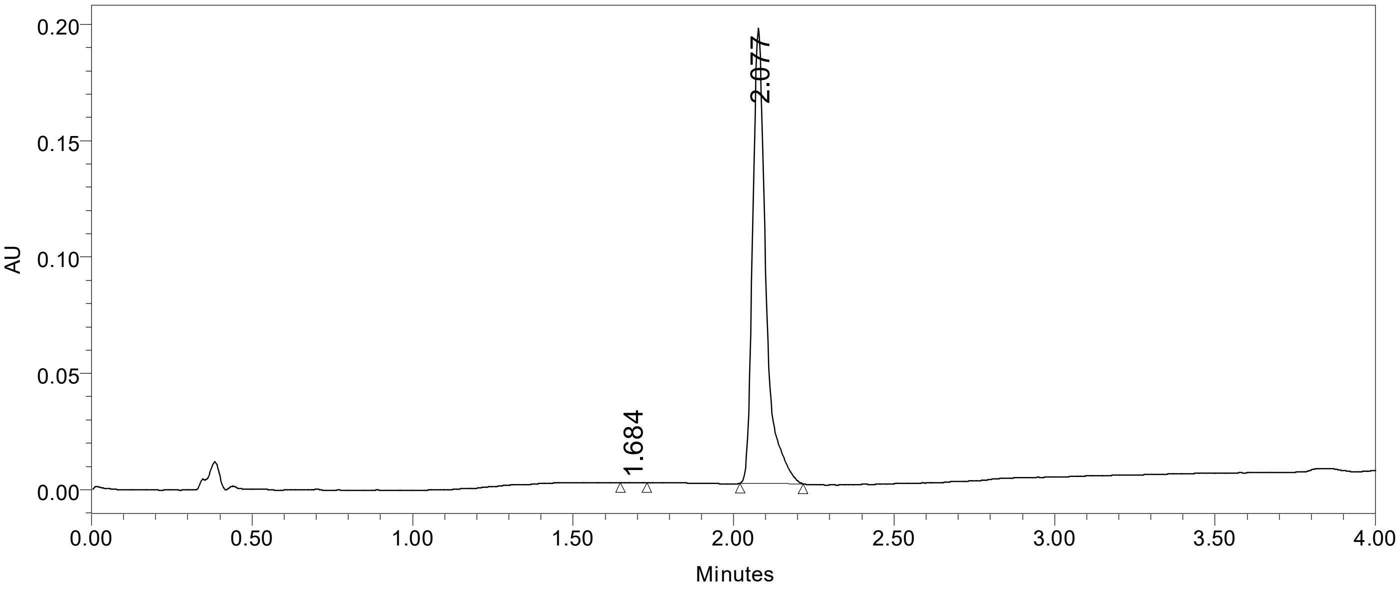

Channel: PDA Spectrum PDA 220.0 nm (PDA Spectrum (190-300)nm)

	RT	Width	Height (mAU)	Resolution	Symmetry	Area	% Area
1	1.684	0.083	0.093	NA	0.9	0.202	0.04
2	2.077	0.197	195.703	6.5	1.6	564.254	99.96

Instrument Method: IH_EtOH_MNH3_10_50_34_35_4min

Stored: 1/4/2026 9:59:28 AM CST

Method Information

Method Comments	Column:Chiralpak IH-3,100×4.6mm I.D.,3um<br>Mobile phase:A: CO2 B:EtOH[0.2%NH3(7M in MeOH), v/v]<br>Gradient:<br>Time A% B%<br>0.0 90 10<br>0.2 90 10<br>2.4 50 50<br>3.4 50 50<br>4.0 90 10<br>Flow rate:3.4mL/min<br>Column temp.:35°C<br>ABPR:2000psi
Method Modified User	CASTJ_CA
Method Locked	No
Method Id	2670
Old Id	
Method Version	2
Method Edit User	
Source S/W Info	Empower 3 Software Build 3471 SPs Installed: Service Release 3 DB ID: 2926695483
