# Supplementary material for: De Novo Generation-Based Design of Potential Computational Hits Targeting the GluN1-GluN2A Receptor
Source: Molecules. 2026 Feb 2;31(3):522. doi: 10.3390/molecules31030522 (PMC12900030; doi:10.3390/molecules31030522)

LC-MS REPORT

Compound ID	: Compound e
Sample ID	: Compound e
Injection Date	:2026/1/22 13:12:05
Injection Vol	: 1ul
Location	:tray1 vail55
Acq Method	:D:\SYSTEM\METHOD\DELIVER_5_95AB_6min_220&254.lcm
Org DataFile	:D:\DATA\2026\2601\260122\Compound e.lcd

Chromatogram

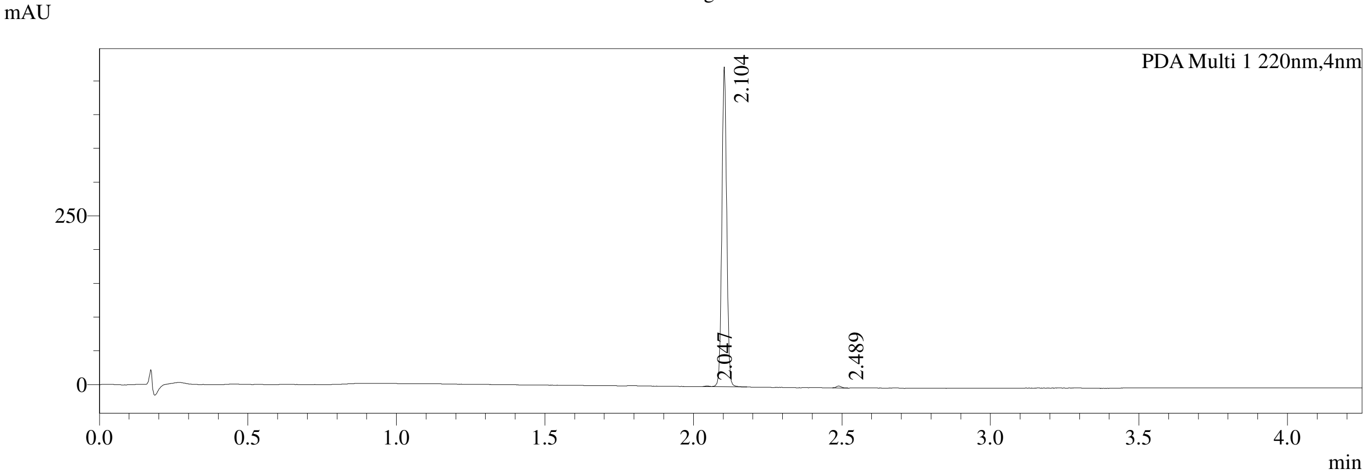

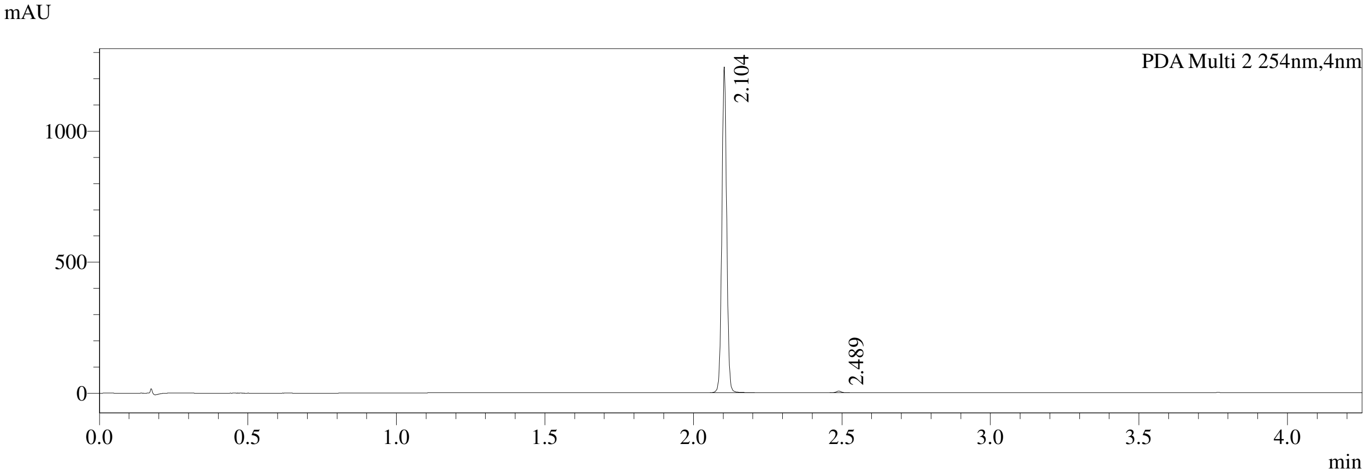

1 PDA Multi 1 / 220nm,4nm
2 PDA Multi 2 / 254nm,4nm

MS Chromatogram

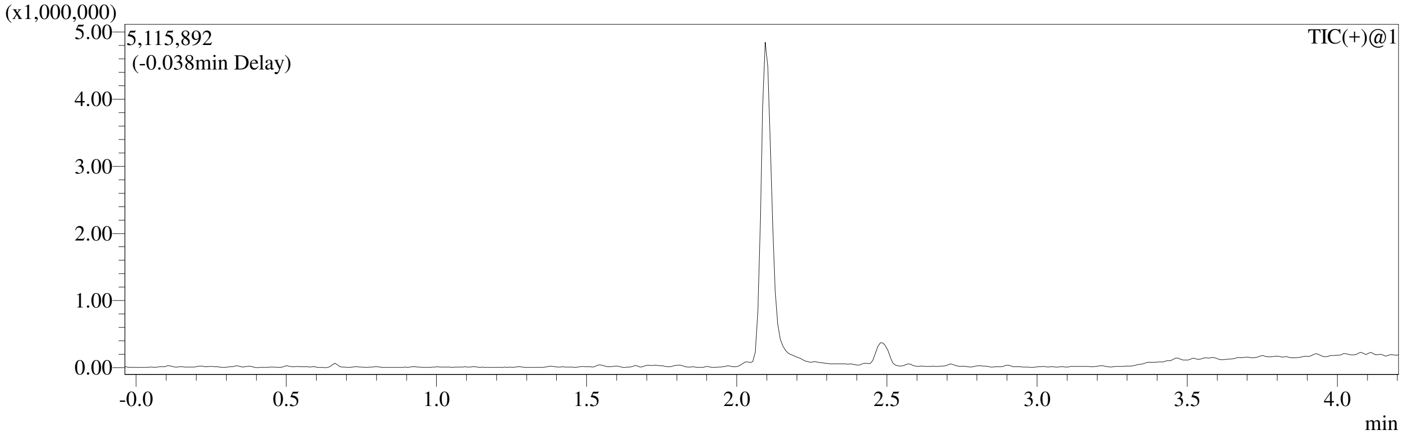

==
Integration Result
==

ELSD

PDA Ch1 220nm

Peak#	Ret. Time	Width	Height	Height%	Area	Area%
1	2.047	0.032	886	0.186	967	0.177
2	2.104	0.032	473831	99.259	541710	99.256
3	2.489	0.033	2650	0.555	3091	0.566

PDA Ch2 254nm

Peak#	Ret. Time	Width	Height	Height%	Area	Area%
1	2.104	0.031	1243017	99.527	1405492	99.507
2	2.489	0.033	5904	0.473	6958	0.493

MS Spectrum RetTime:

2.095-2.112 Positive(ESI+) Datafile: D:\DATA\2026\2601\260122\Compound e.lcd Intensity

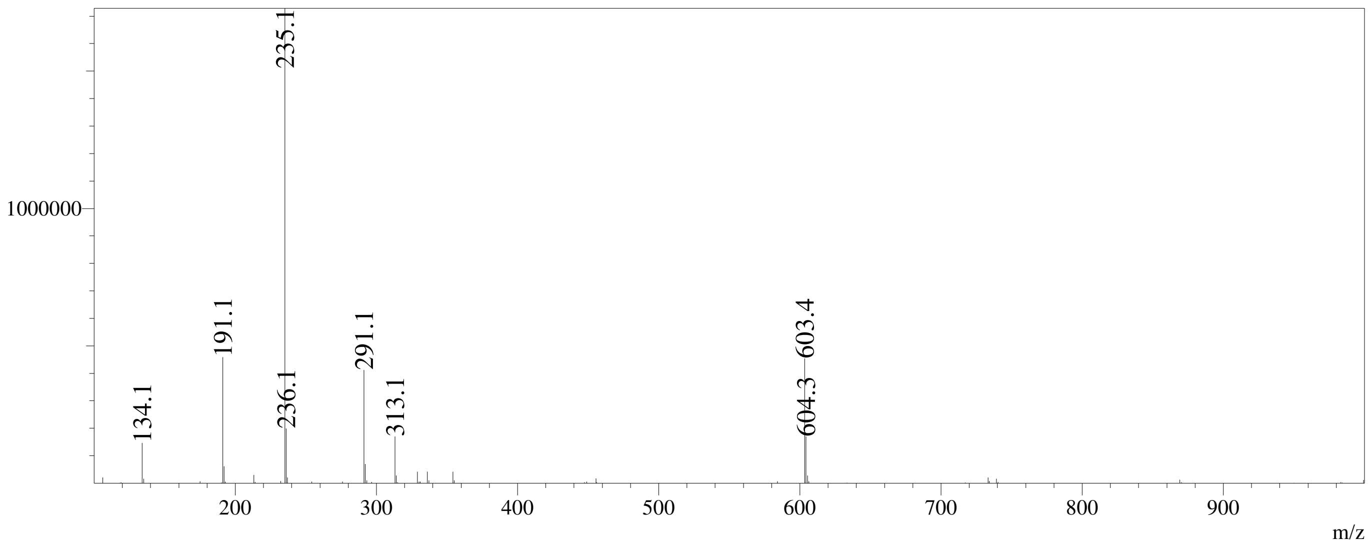

Supplement: Supplementary file 1 [file molecules-31-00522-s001.zip › ESM_F3_Characterization of Compounds in Scheme 3/Compound e_LC-MS.pdf]
