# Supplementary material for: De Novo Generation-Based Design of Potential Computational Hits Targeting the GluN1-GluN2A Receptor
Source: Molecules. 2026 Feb 2;31(3):522. doi: 10.3390/molecules31030522 (PMC12900030; doi:10.3390/molecules31030522)
Supplement: Supplementary file 1 [file molecules-31-00522-s001.zip › ESM_F3_Characterization of Compounds in Scheme 3/Compound f_HPLC.pdf]

HPLC REPORT

Compound ID	:Compound f
Sample ID	: Compound f
Injection Date	:2026/1/23 11:36:48
Injection Vol	: 1ul
Location	:tray1 vail104
Acq Method	:D:\SYSTEM\METHOD\10_80AB_6min.lcm
Org DataFile	:D:\DATA\2026\2601\260123\Compound f.lcd
Instrument	:02-HPLC-0067

Chromatogram

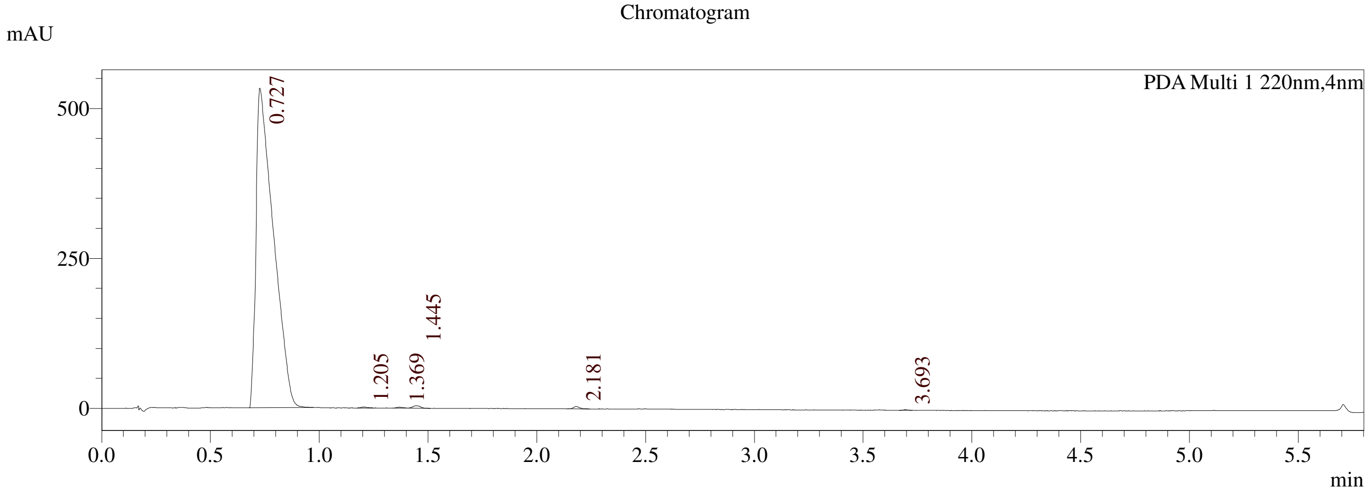

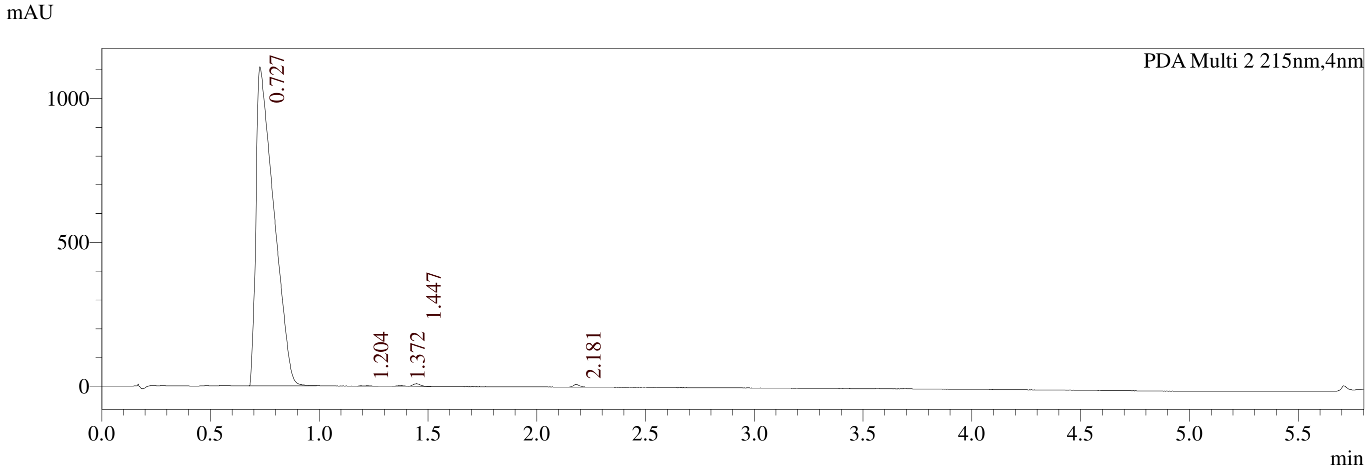

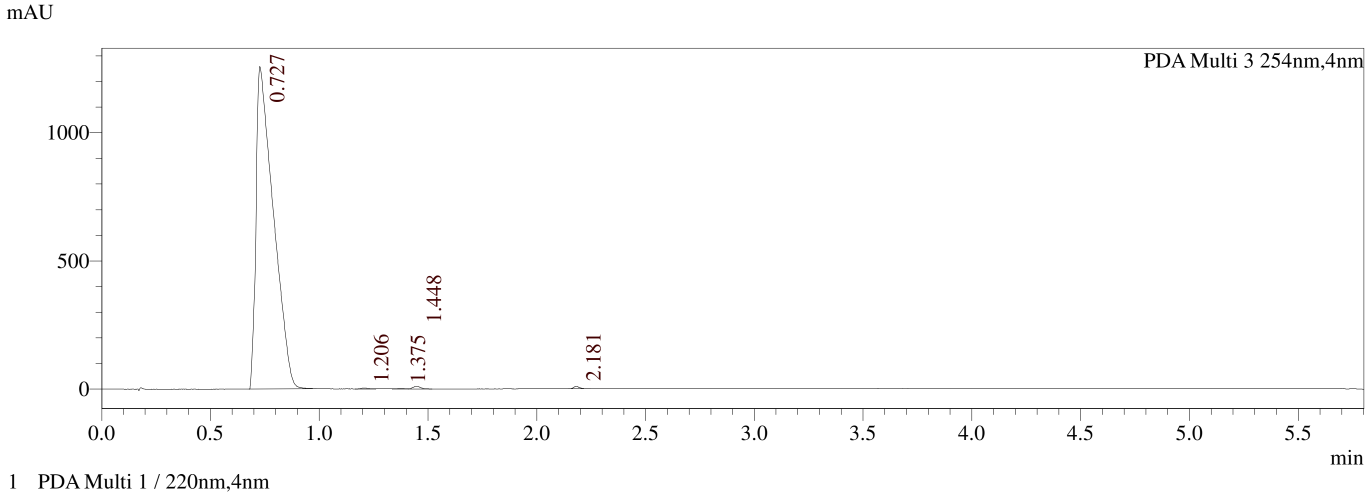

1 PDA Multi 1 / 220nm,4nm
2 PDA Multi 2 / 215nm,4nm
3 PDA Multi 3 / 254nm,4nm

==
Integration Result
==

PDA Ch1 220nm

Peak#	Ret. Time	Width	Height	Height%	Area	Area%
1	0.727	0.162	532901	97.717	2969284	99.111
2	1.205	0.060	1821	0.334	3829	0.128
3	1.369	0.058	1481	0.272	3237	0.108
4	1.445	0.062	4212	0.772	10243	0.342
5	2.181	0.052	4000	0.733	7744	0.258
6	3.693	0.042	939	0.172	1582	0.053

PDA Ch2 215nm

Peak#	Ret. Time	Width	Height	Height%	Area	Area%
1	0.727	0.166	1108710	97.929	6330237	99.211
2	1.204	0.060	3507	0.310	7870	0.123
3	1.372	0.058	2008	0.177	4554	0.071
4	1.447	0.061	8859	0.783	20688	0.324
5	2.181	0.050	9067	0.801	17221	0.270

PDA Ch3 254nm

Peak#	Ret. Time	Width	Height	Height%	Area	Area%
1	0.727	0.160	1256729	98.075	6973681	99.211
2	1.206	0.062	3392	0.265	8046	0.114
3	1.375	0.069	1791	0.140	4811	0.068
4	1.448	0.064	9886	0.772	24091	0.343
5	2.181	0.050	9597	0.749	18540	0.264
