# Supplementary material for: De Novo Generation-Based Design of Potential Computational Hits Targeting the GluN1-GluN2A Receptor
Source: Molecules. 2026 Feb 2;31(3):522. doi: 10.3390/molecules31030522 (PMC12900030; doi:10.3390/molecules31030522)

LC-MS REPORT

Compound ID : Compound f
Sample ID : Compound f
Injection Date :2026/1/22 20:14:55
Injection Vol : 1ul
Location :tray1 vail26
Acq Method :D:\SYSTEM\METHOD\DELIVER_5_95AB_6min_220&254.lcm
Org DataFile :D:\DATA\2026\2601\260122\Compound f.lcd

Chromatogram

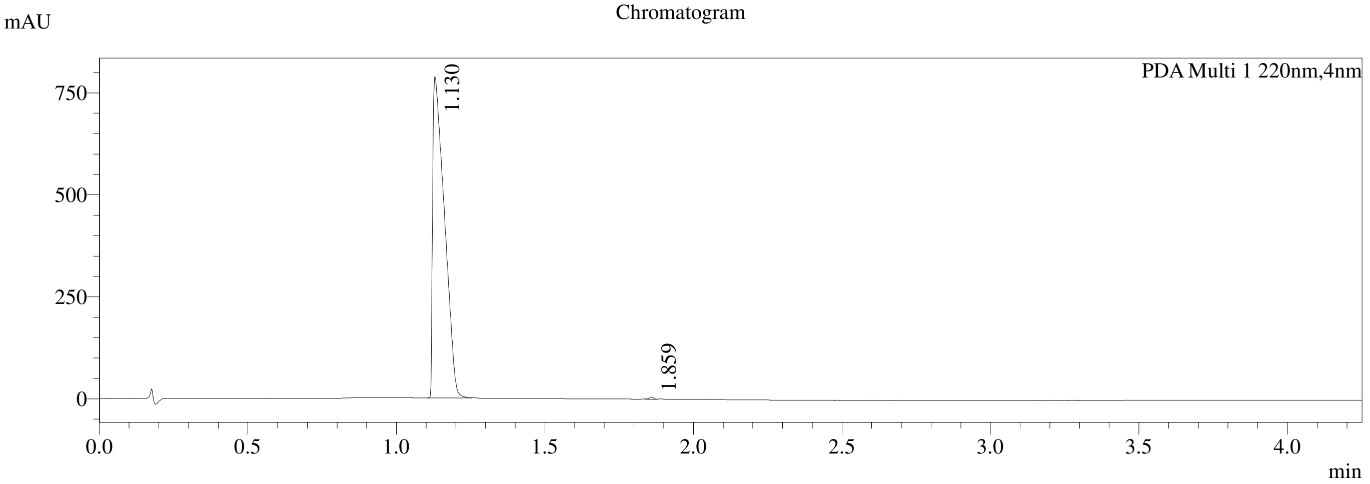

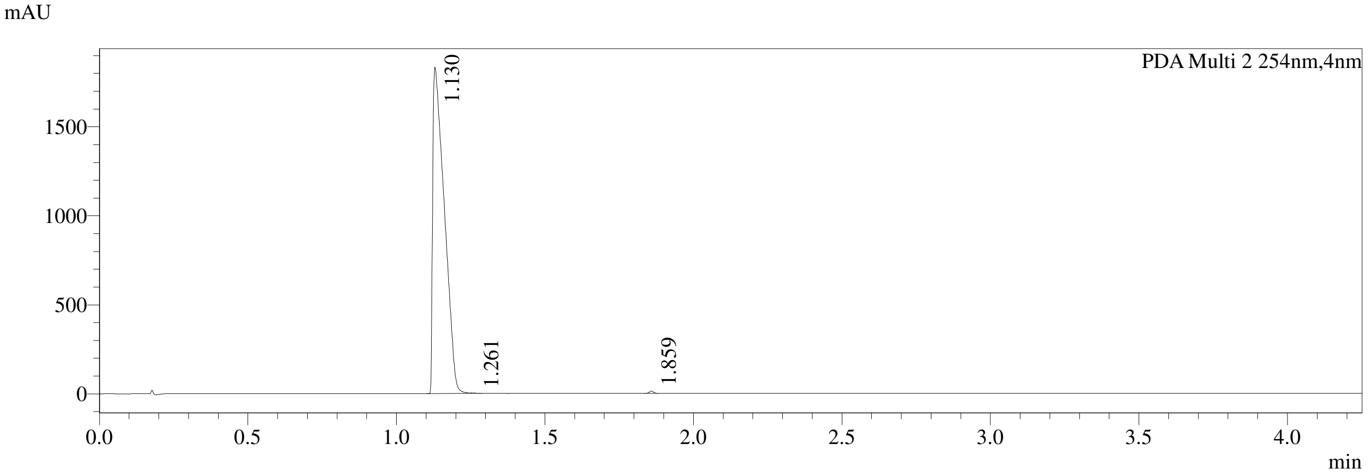

1 PDA Multi 1 / 220nm,4nm
2 PDA Multi 2 / 254nm,4nm

MS Chromatogram

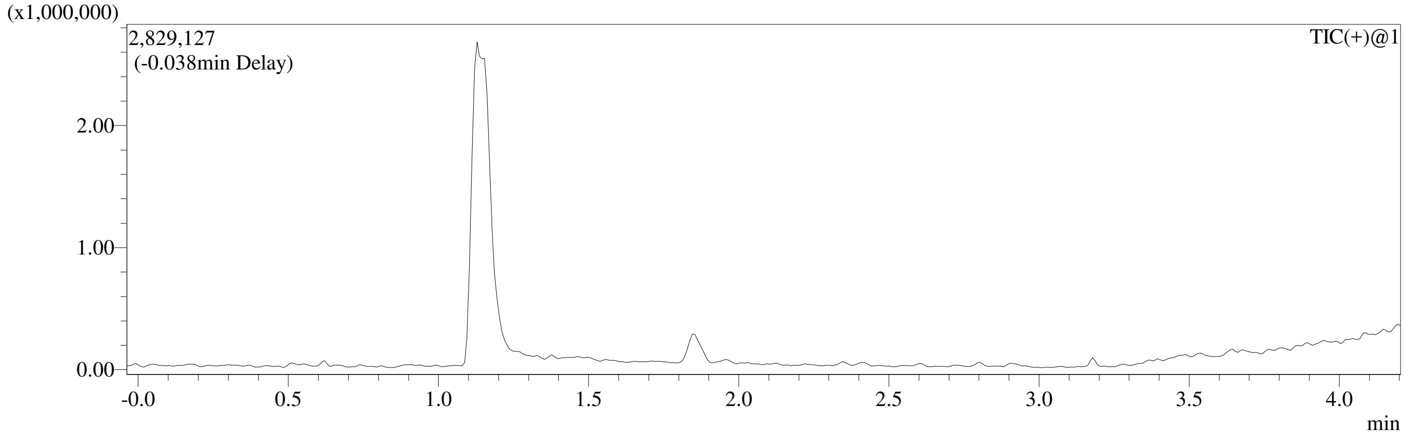

==
Integration Result
==

ELSD

PDA Ch1 220nm

Peak#	Ret. Time	Width	Height	Height%	Area	Area%
1	1.130	0.083	787908	99.368	2169686	99.778
2	1.859	0.028	5012	0.632	4832	0.222

PDA Ch2 254nm

Peak#	Ret. Time	Width	Height	Height%	Area	Area%
1	1.130	0.082	1834256	99.197	5001339	99.708
2	1.261	0.045	2526	0.137	2540	0.051
3	1.859	0.028	12323	0.666	12083	0.241

MS Spectrum RetTime:

1.120-1.137 Positive(ESI+) Datafile: D:\DATA\2026\2601\260122\Compound f.lcd Intensity

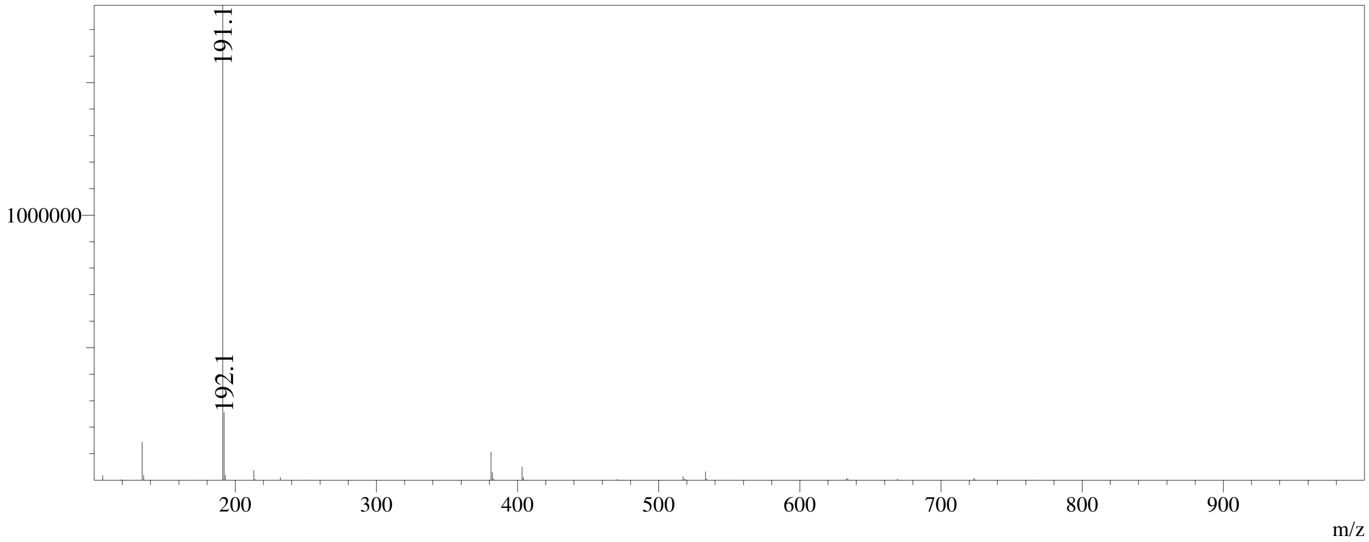

Supplement: Supplementary file 1 [file molecules-31-00522-s001.zip › ESM_F3_Characterization of Compounds in Scheme 3/Compound f_LC-MS.pdf]
