# Supplementary material for: De Novo Generation-Based Design of Potential Computational Hits Targeting the GluN1-GluN2A Receptor
Source: Molecules. 2026 Feb 2;31(3):522. doi: 10.3390/molecules31030522 (PMC12900030; doi:10.3390/molecules31030522)
Supplement: Supplementary file 1 [file molecules-31-00522-s001.zip › ESM_F3_Characterization of Compounds in Scheme 3/Compound f_OR.pdf]

# Optical Rotation Report

Measurement Date : 01/23/2026

Method Name : GTM-11-SR-20

Sample ID : Compound f

Compound ID : Compound f

Solvent : $CHCl_3$

Set Temperature : 20.0°C

| N | Avg. | Std.Dev. | %RSD | Min | Max |
|---|---|---|---|---|---|
| 3 | 66.00 | 0.00 | 0.00 | 66.00 | 66.00 |

| S.No | Result | Scale | OR °Arc | WLG.nm | Lg.mm | Conc.g/100mL | Temp |
|---|---|---|---|---|---|---|---|
| 1 | 66.00 | SR | 0.066 | 589 | 50 | 0.2000 | 20.0°C |
| 2 | 66.00 | SR | 0.066 | 589 | 50 | 0.2000 | 20.0°C |
| 3 | 66.00 | SR | 0.066 | 589 | 50 | 0.2000 | 20.0°C |
