# Supplementary material for: De Novo Generation-Based Design of Potential Computational Hits Targeting the GluN1-GluN2A Receptor
Source: Molecules. 2026 Feb 2;31(3):522. doi: 10.3390/molecules31030522 (PMC12900030; doi:10.3390/molecules31030522)
Supplement: Supplementary file 1 [file molecules-31-00522-s001.zip › ESM_F3_Characterization of Compounds in Scheme 3/Compound f_SFC.pdf]

Chiral SFC Report

Sample Information

Sample ID:	Compound f
Compound ID:	Compound f
Date Acquired:	1/23/2026 11:40:20 PM CST
Date Processed:	1/26/2026 9:44:40 AM CST
Injection Volume:	3.00 ul
Vial:	2:F,7
Acq Method :	IG_EtOH_MNH3_10_50_34_35_4min
Raw Data:	D:\Data\ID_m32_result_ 61288
Project Name:	2026\CASTJ_CA\SFC-M-20260104
Instrument:	CAS-02-ANA-SFC-M(Waters UPCC with SQD2)
Label:	No Racemate

Test Results

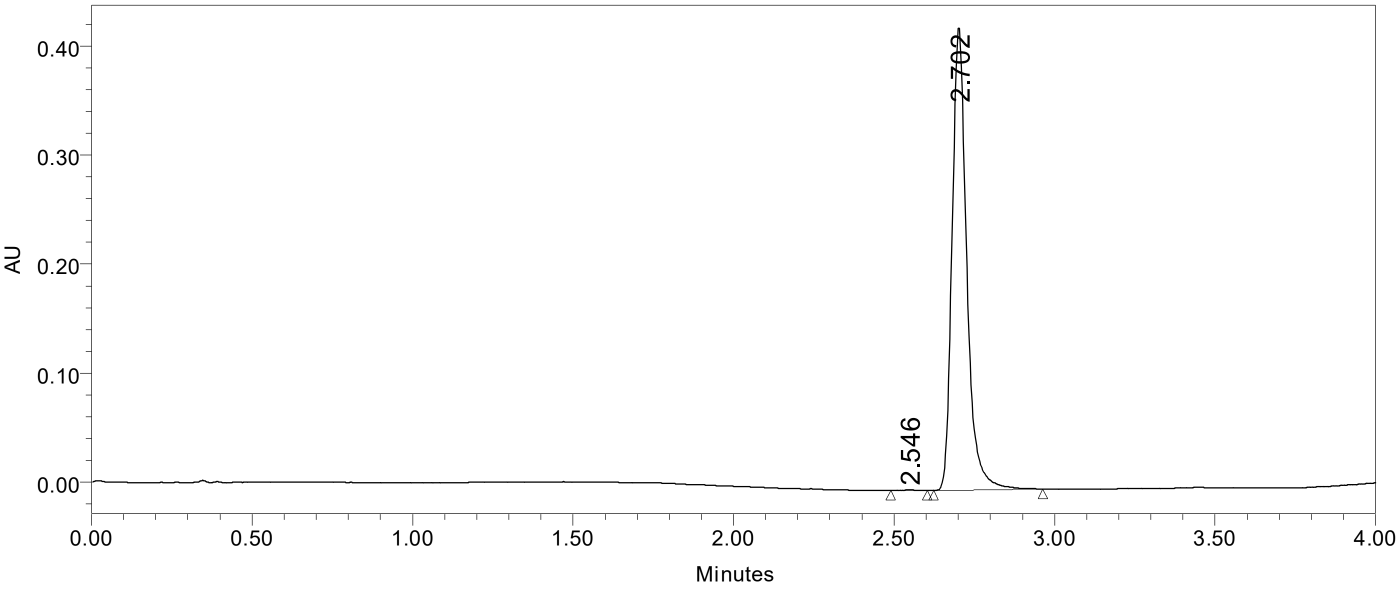

Channel: PDA Spectrum PDA 220.0 nm (PDA Spectrum (190-300)nm)

	RT	Width	Height (mAU)	Resolution	Symmetry	Area	% Area(*)
1	2.546	0.113	0.765	NA	1.2	2.177	0.16
2	2.702	0.340	425.252	2.0	1.3	1357.961	99.84

MS Spectrum

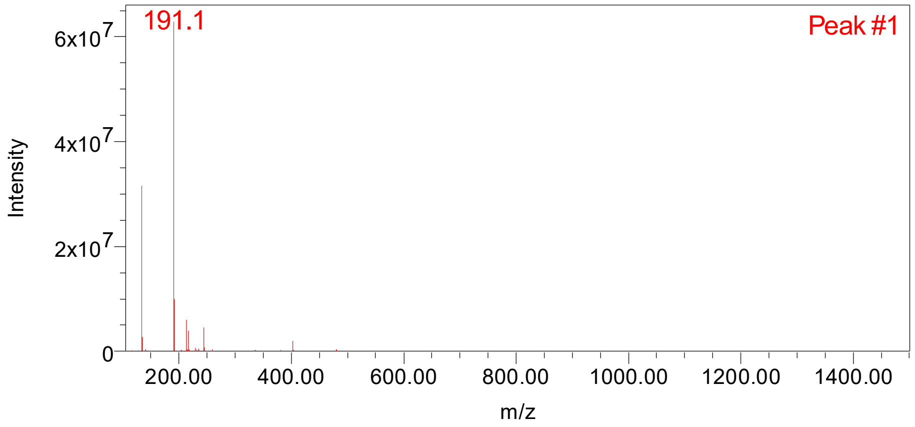

SampleName: T207841_Int.4_IG_EtOH Retention Time 2.546

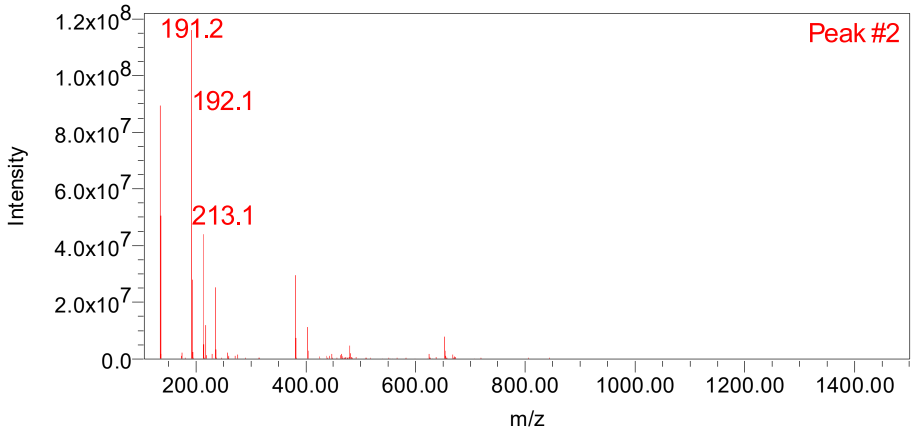

SampleName: Compound f_IG_EtOH Retention Time 2.702

Instrument Method: IG_EtOH_MNH3_10_50_34_35_4min

Stored: 12/31/2025 4:04:55 PM CST

Method Information

Method Comments	Column:Chiralpak IG-3,100×4.6mm I.D.,3um<br>Mobile phase:A: CO2 B:EtOH[0.2%NH3(7M in MeOH), v/v]<br>Gradient:<br>Time A% B%<br>0.0 90 10<br>0.2 90 10<br>2.4 50 50<br>3.4 50 50<br>4.0 90 10<br>Flow rate:3.4mL/min<br>Column temp.:35°C<br>ABPR:2000psi
Method Modified User	CASTJ_CA
Method Locked	No
Method Id	2114
Old Id	
Method Version	1
Method Edit User	
Source S/W Info	Empower 3 Software Build 3471 SPs Installed: Service Release 3 DB ID: 2926695483
