# Supplementary figures and images for: De Novo Generation-Based Design of Potential Computational Hits Targeting the GluN1-GluN2A Receptor
Source: Molecules. 2026 Feb 2;31(3):522. doi: 10.3390/molecules31030522 (PMC12900030; doi:10.3390/molecules31030522)

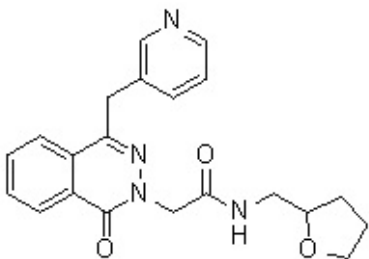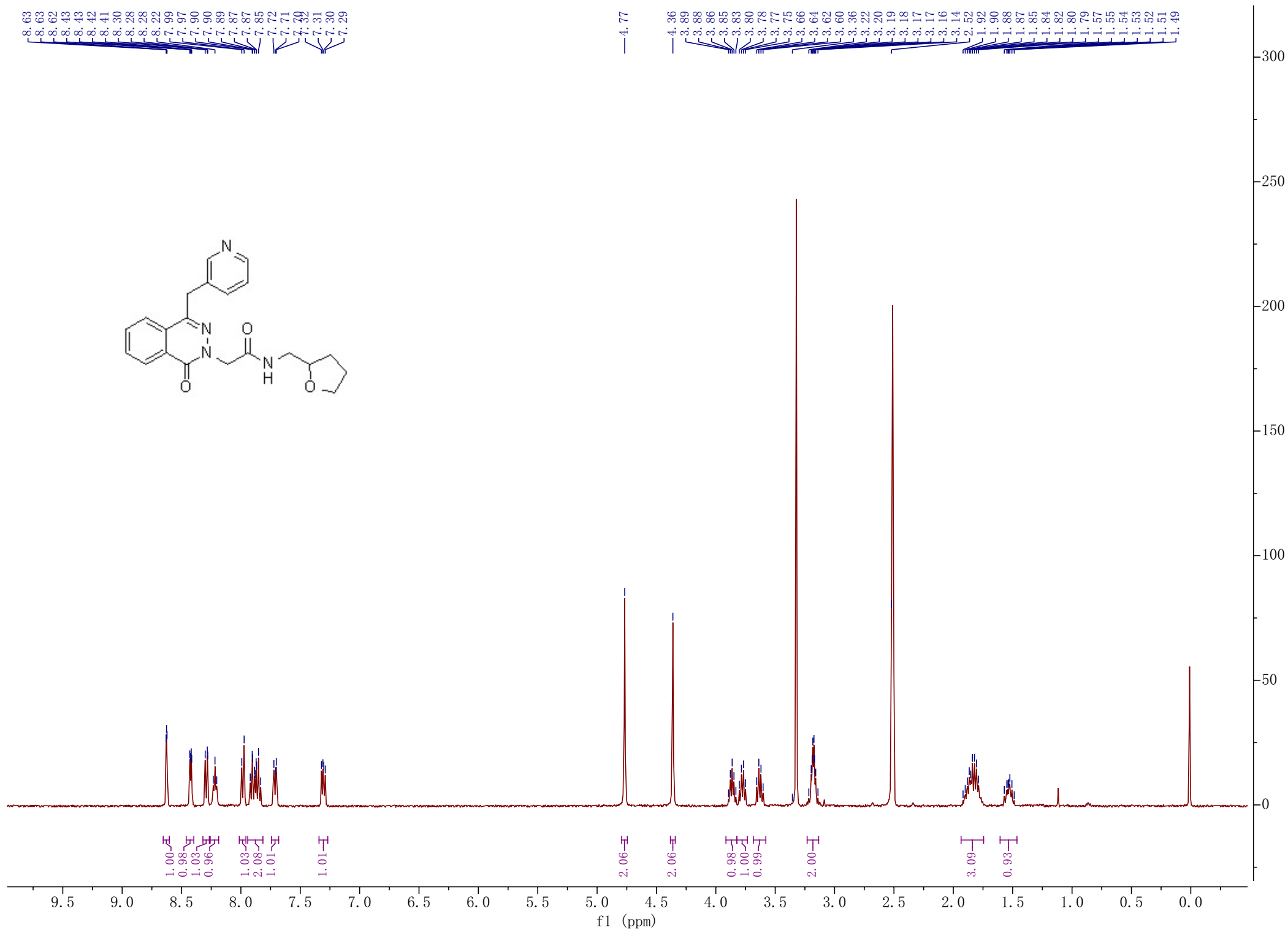

Supplement: Supplementary file 1 [file molecules-31-00522-s001.zip › ESM_F1_Characterization of Compounds in Scheme 1/A1_1H NMR.pdf]

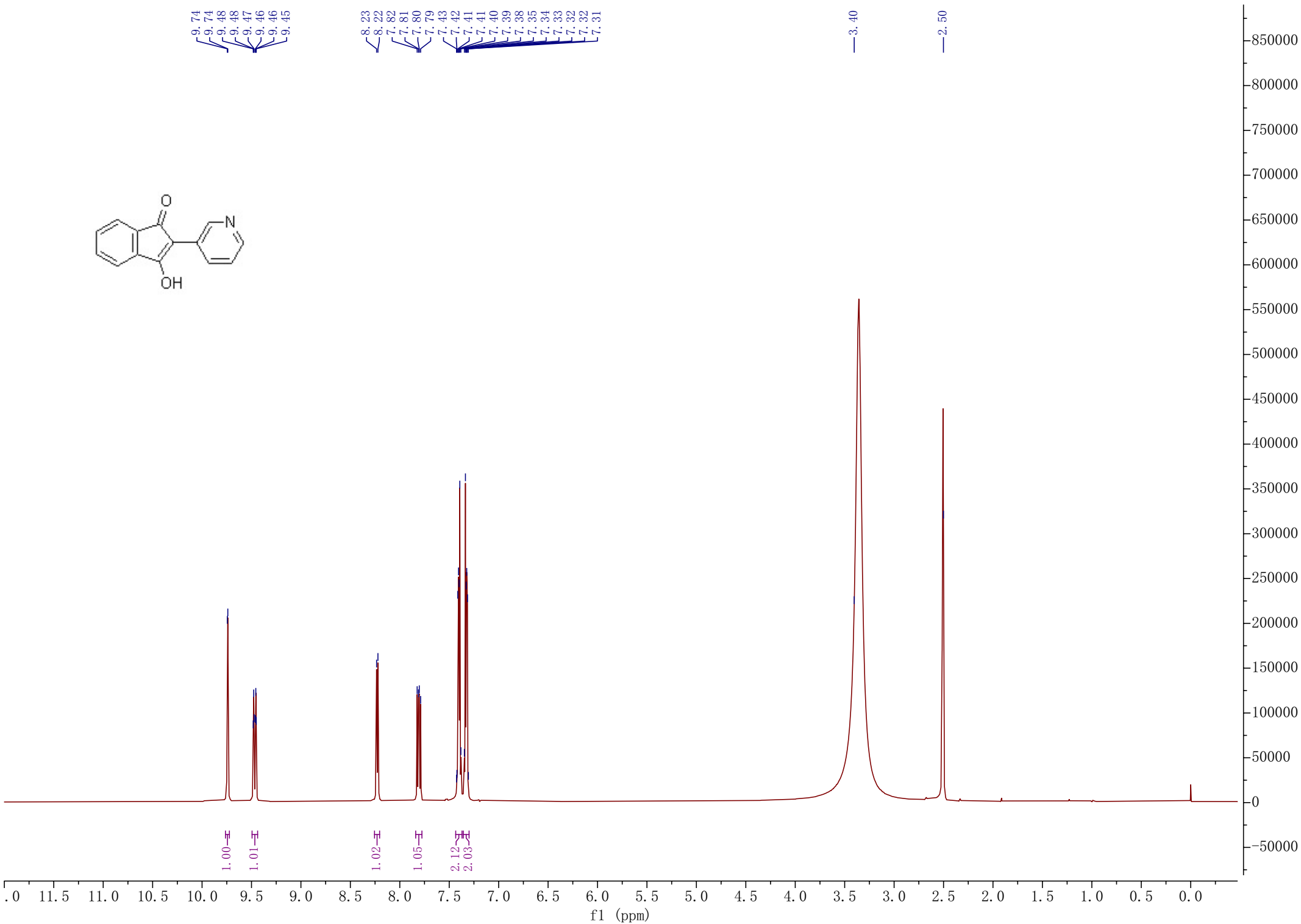

Supplement: Supplementary file 1 [file molecules-31-00522-s001.zip › ESM_F1_Characterization of Compounds in Scheme 1/Compound a_1H NMR.pdf]

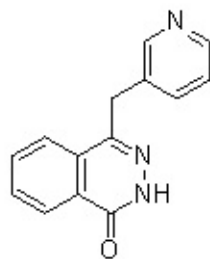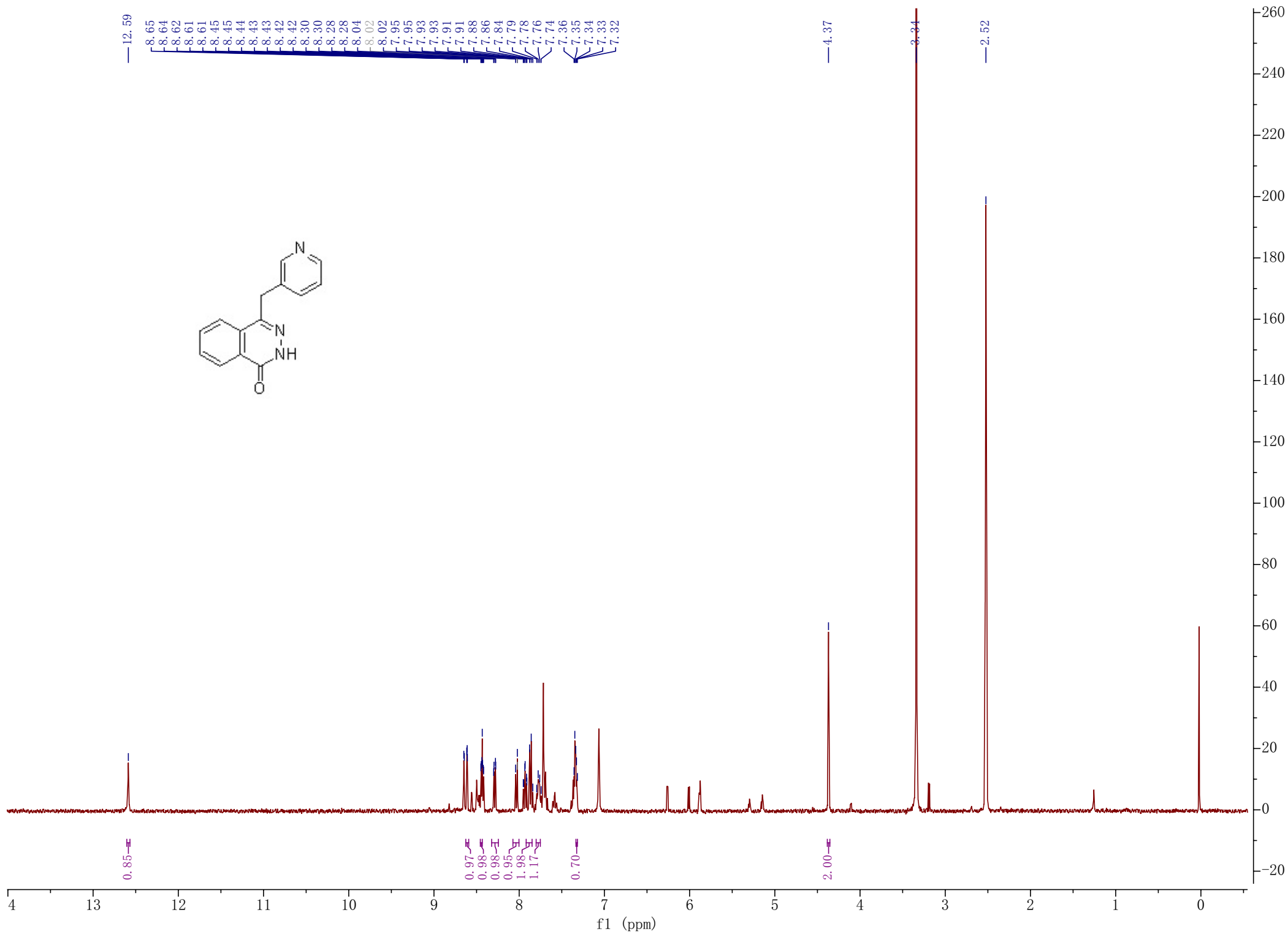

Supplement: Supplementary file 1 [file molecules-31-00522-s001.zip › ESM_F1_Characterization of Compounds in Scheme 1/Compound b_1H NMR(crude).pdf]

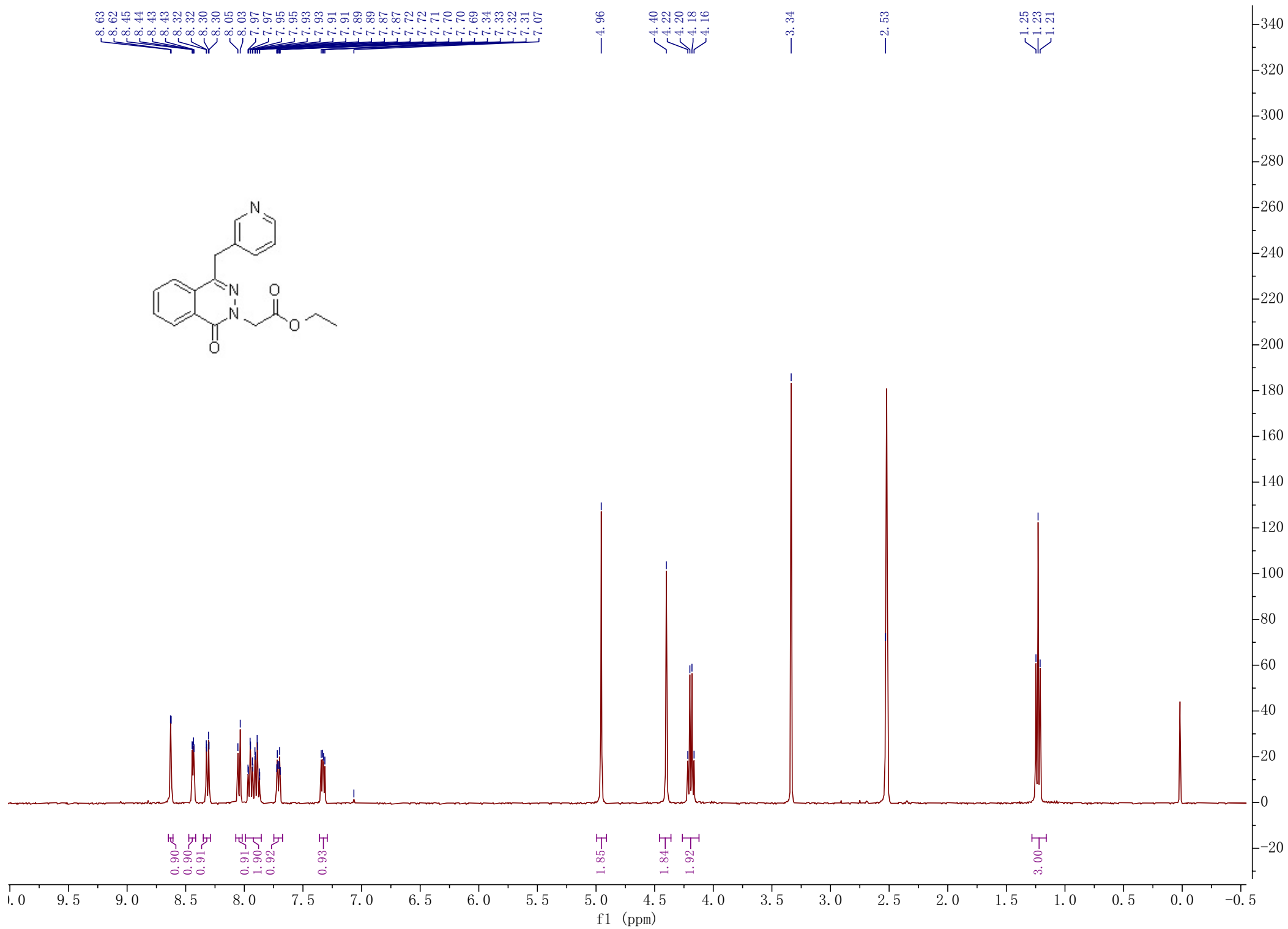

Supplement: Supplementary file 1 [file molecules-31-00522-s001.zip › ESM_F1_Characterization of Compounds in Scheme 1/Compound c_1H NMR.pdf]

Batch No.:

Compound ID: A2 DMSO

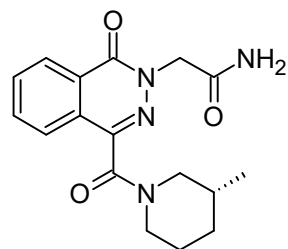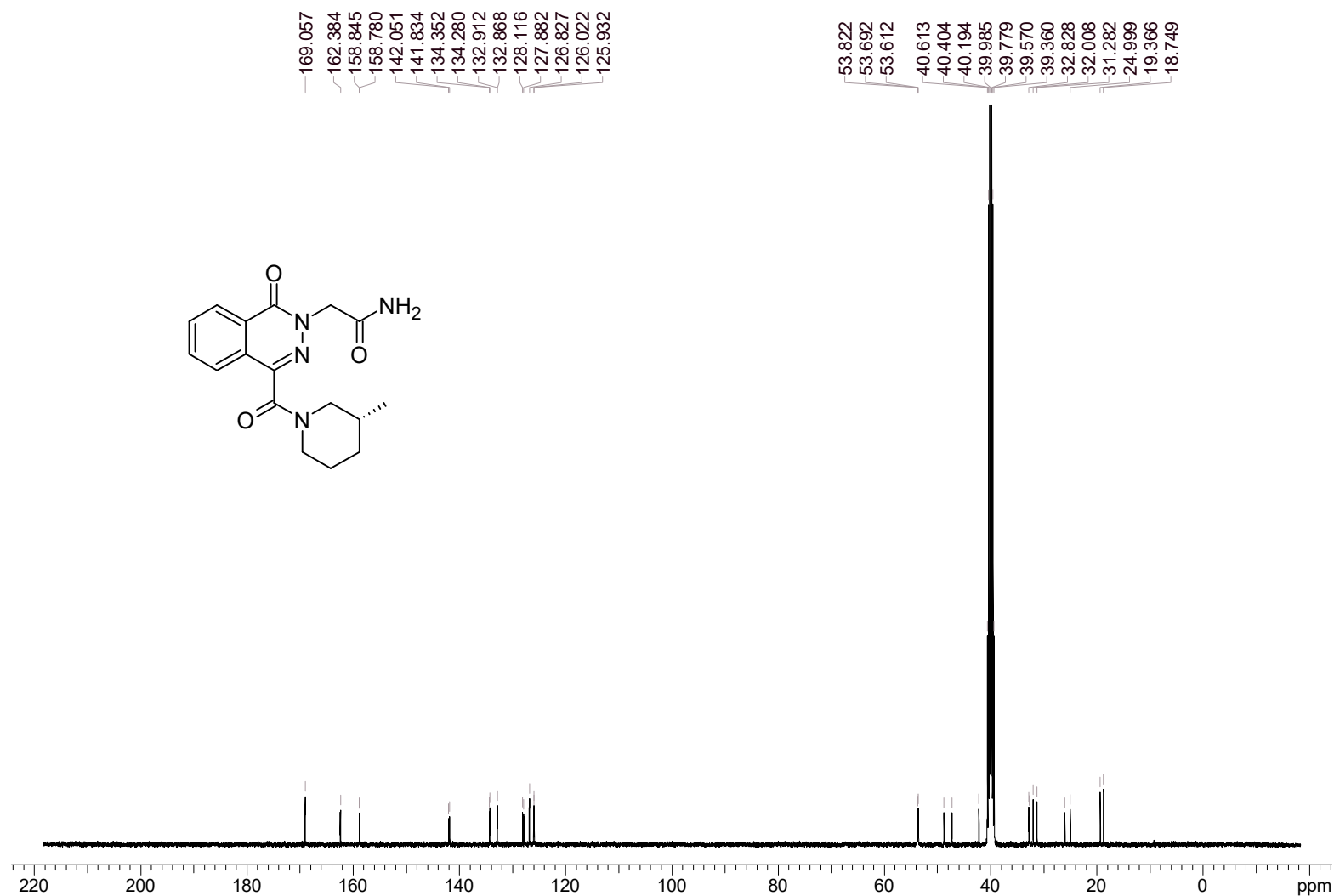

Supplement: Supplementary file 1 [file molecules-31-00522-s001.zip › ESM_F2_Characterization of Compounds in Scheme 2/A2_13C NMR.pdf]

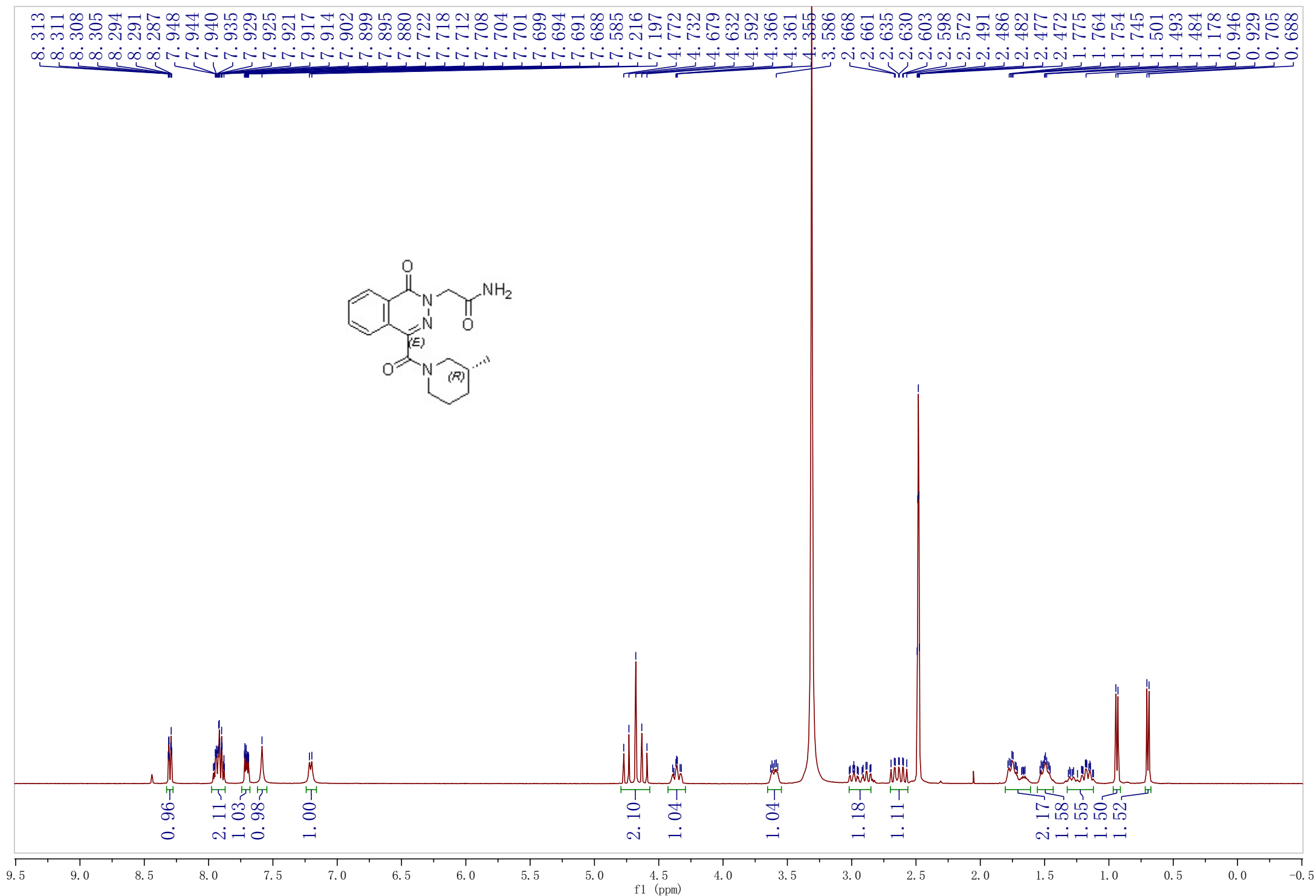

Supplement: Supplementary file 1 [file molecules-31-00522-s001.zip › ESM_F2_Characterization of Compounds in Scheme 2/A2_1H NMR.pdf]

Batch No.:

Compound ID: Compound d DMSO

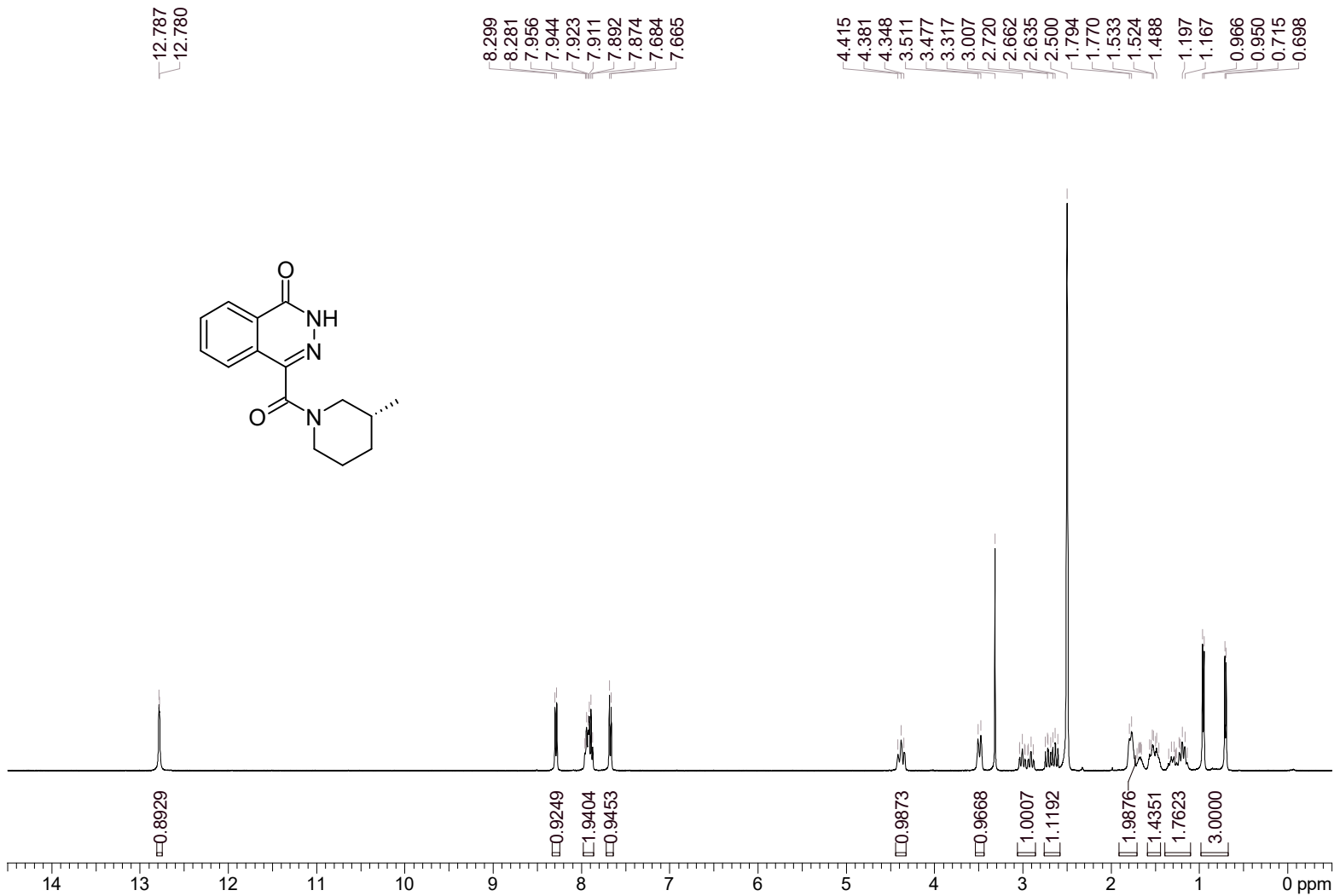

Supplement: Supplementary file 1 [file molecules-31-00522-s001.zip › ESM_F2_Characterization of Compounds in Scheme 2/Compound d_1H NMR.pdf]

Batch No.: Compound ID: A3 DMSO

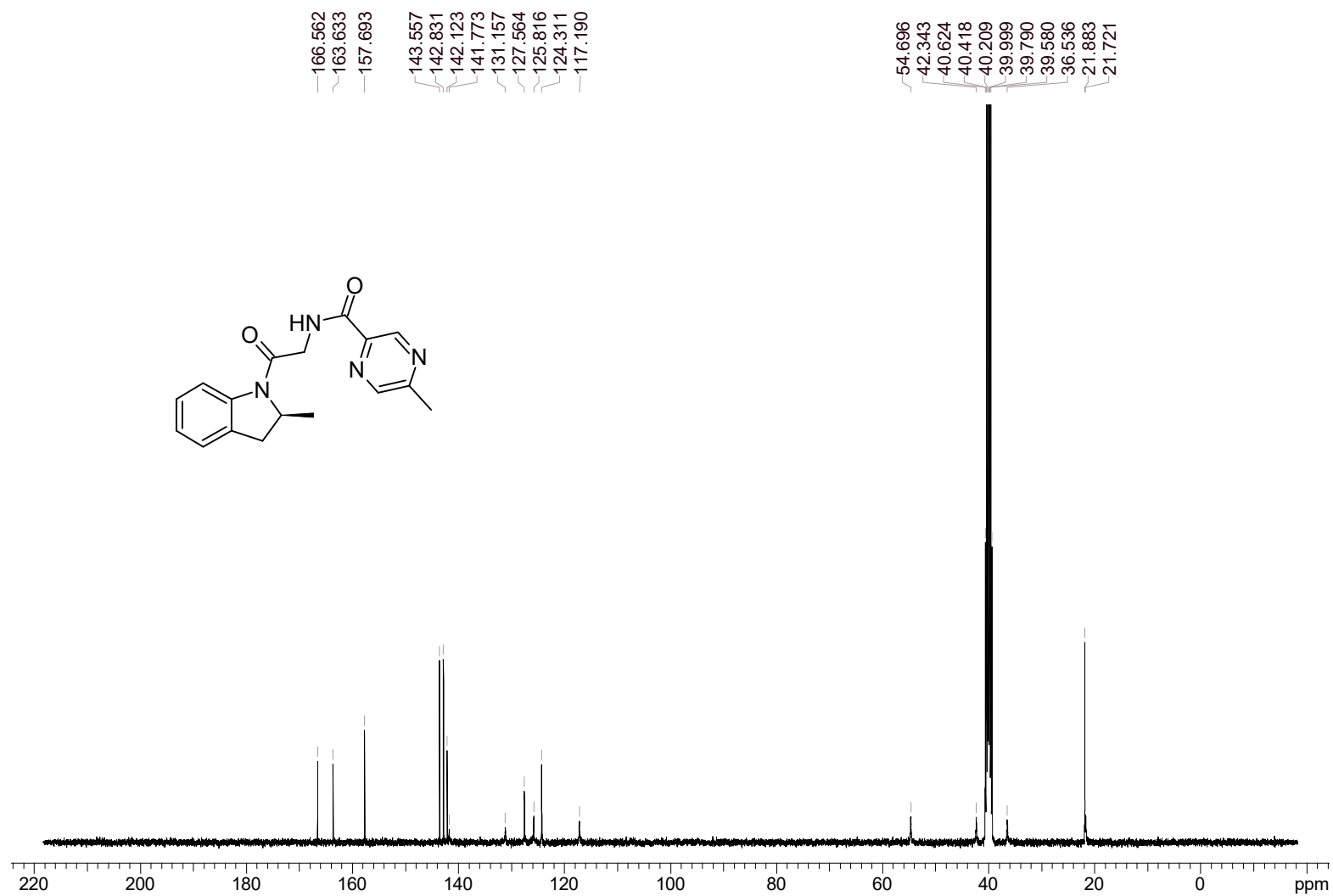

Supplement: Supplementary file 1 [file molecules-31-00522-s001.zip › ESM_F3_Characterization of Compounds in Scheme 3/A3_13C NMR.pdf]

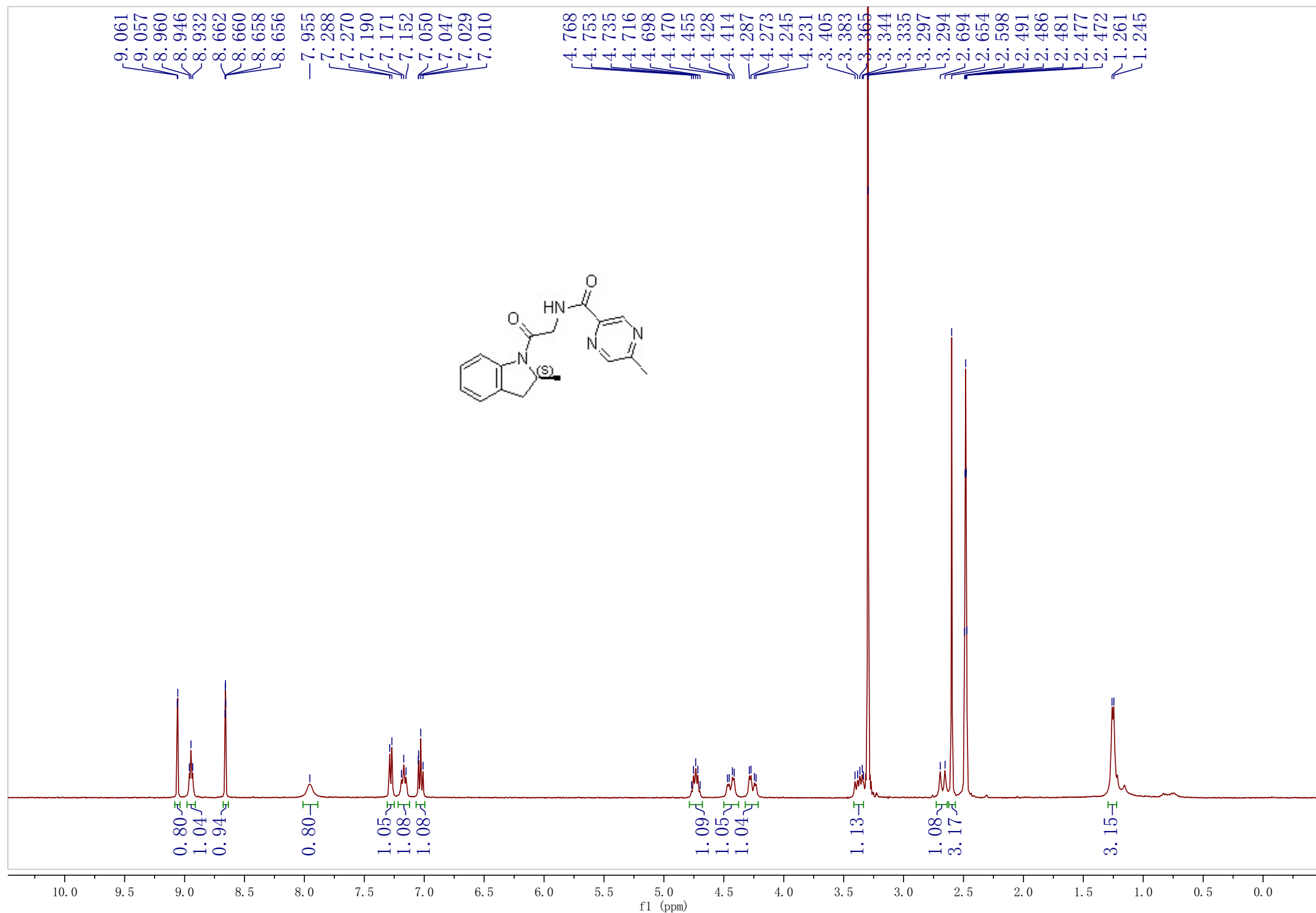

Supplement: Supplementary file 1 [file molecules-31-00522-s001.zip › ESM_F3_Characterization of Compounds in Scheme 3/A3_1H NMR.pdf]

Batch No.:

Compound ID: Compound e

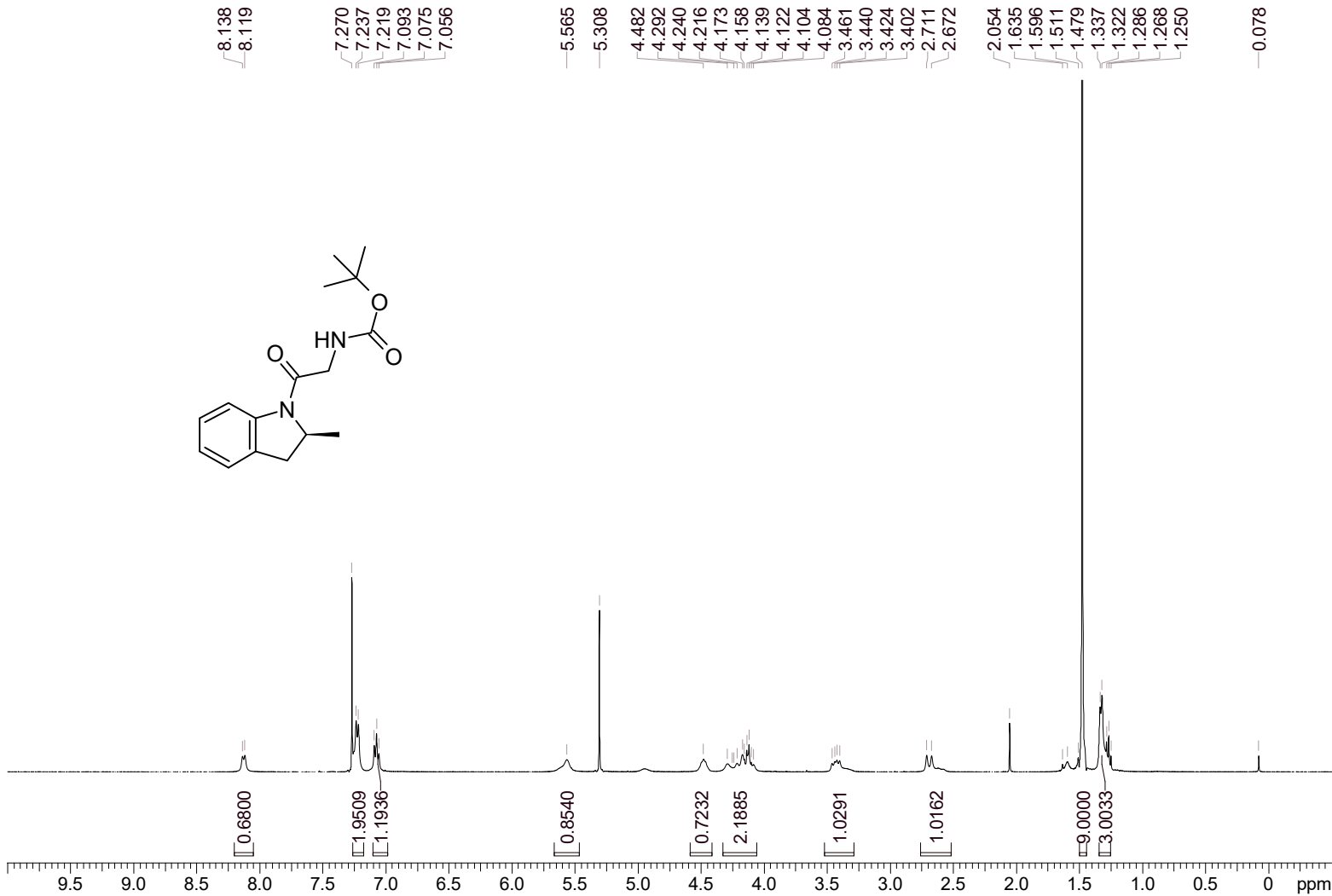

Supplement: Supplementary file 1 [file molecules-31-00522-s001.zip › ESM_F3_Characterization of Compounds in Scheme 3/Compound e_1H NMR.pdf]

Batch No.:

Compound ID: Compound f DMSO

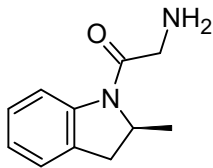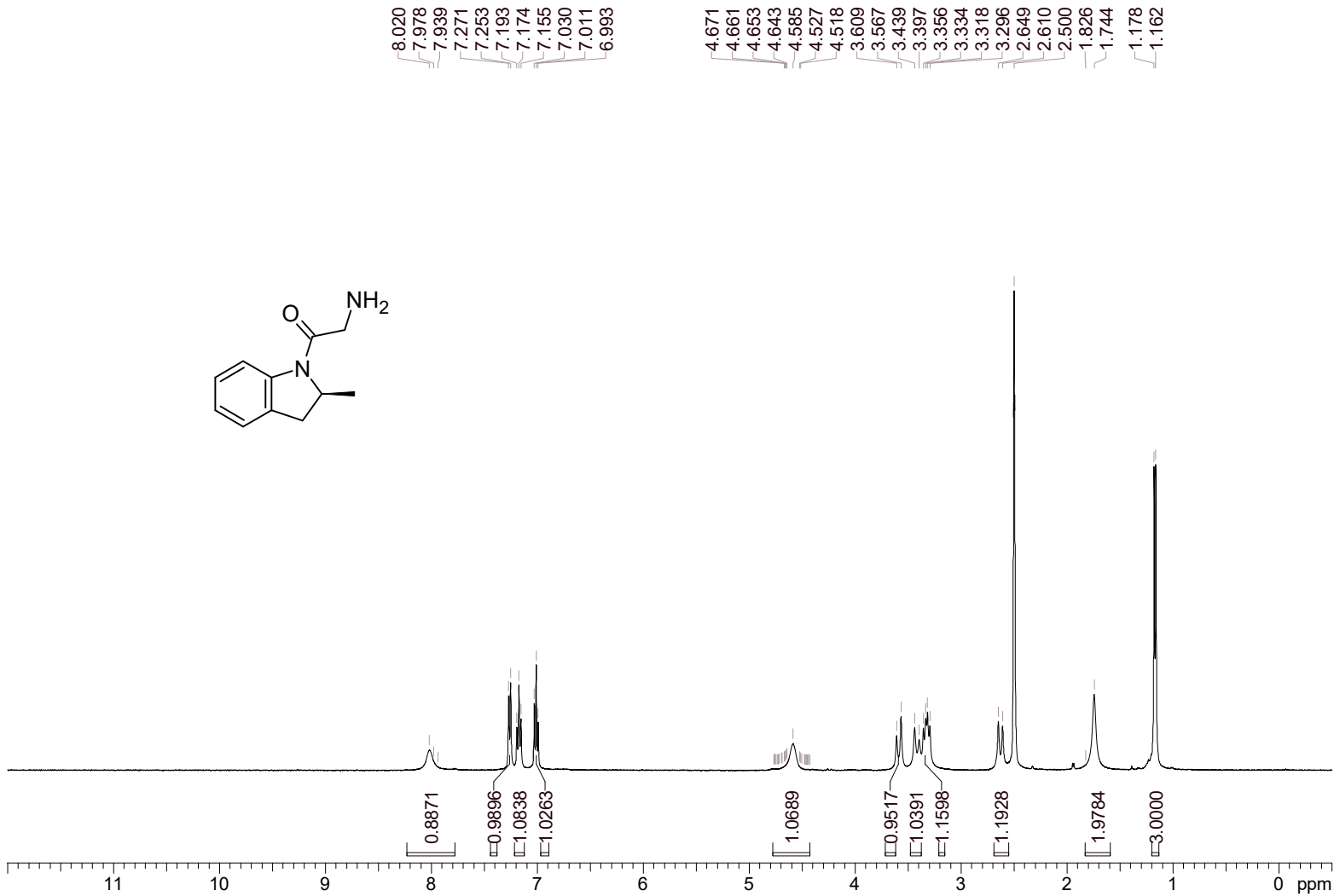

Supplement: Supplementary file 1 [file molecules-31-00522-s001.zip › ESM_F3_Characterization of Compounds in Scheme 3/Compound f_1H NMR.pdf]
