# Supplementary material for: De Novo Generation-Based Design of Potential Computational Hits Targeting the GluN1-GluN2A Receptor
Source: Molecules. 2026 Feb 2;31(3):522. doi: 10.3390/molecules31030522 (PMC12900030; doi:10.3390/molecules31030522)
Supplement: Supplementary file 1 [file A2_HPLC.pdf › ESM_F2_Characterization of Compounds in Scheme 2/A2_HPLC.pdf]

Data File Z:\DATA\DATA\HPLC-6(73)\2025\202511\251106\A2.D
Sample Name: A2

===
Acq. Operator   :   Seq. Line :   1
Acq. Instrument : HPLC_73#   Location : Vial 91
Injection Date : 11/7/2025 7:35:03 AM   Inj :   1
                                                    Inj Volume : 5.0 µl
Different Inj Volume from Sequence !   Actual Inj Volume : 10.0 µl
Acq. Method   : C:\CHEM32\1\METHODS\NH4HCO3.M

Analysis Method : C:\CHEM32\1\METHODS\DEF_LC.M
Sample Info   :   `````

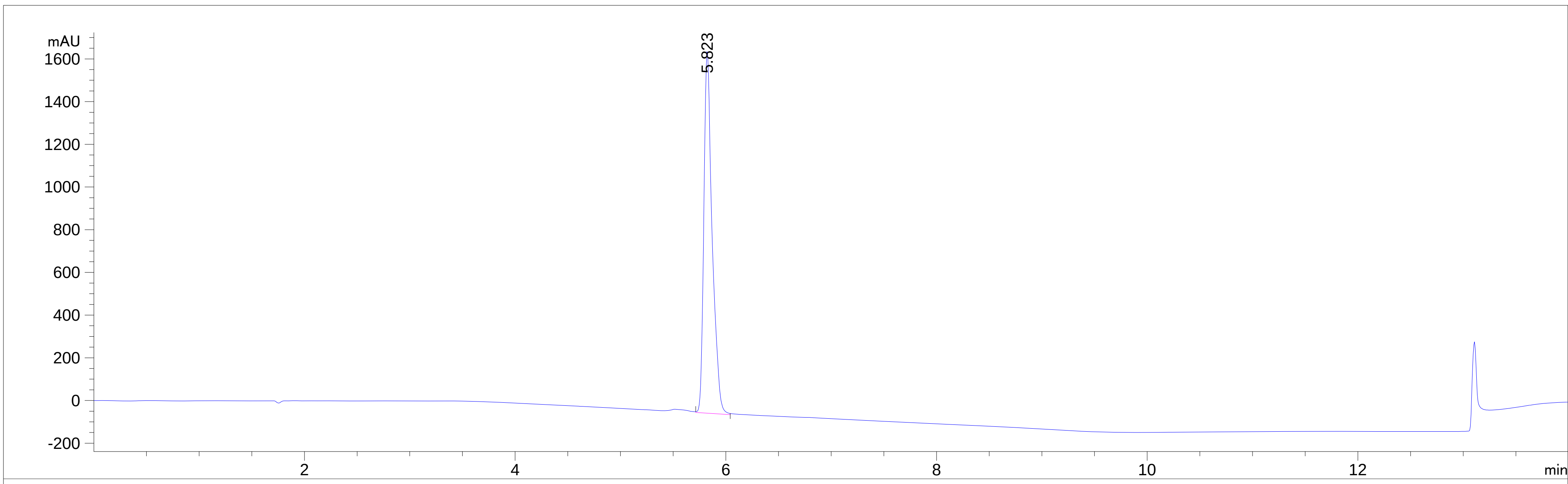

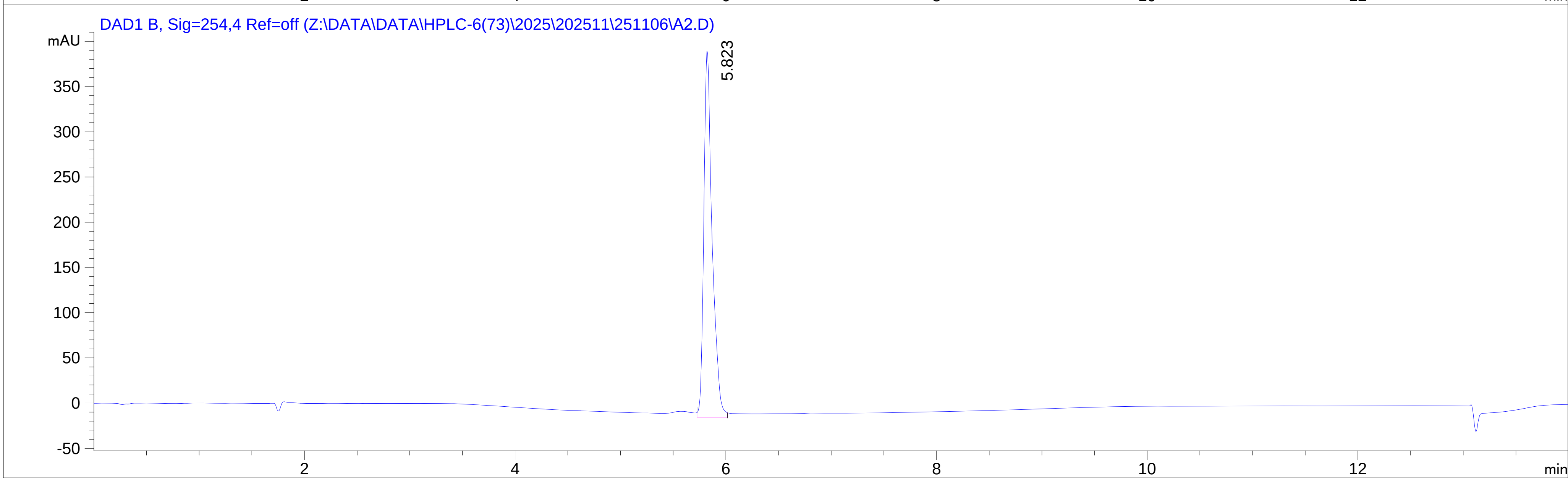

===
Area Percent Report
===

Sorted By   :   Signal
Multiplier: :   1.0000
Dilution: :   1.0000
Use Multiplier & Dilution Factor with ISTDs

Signal 1: DAD1 A, Sig=220,4 Ref=off

Peak #	RetTime [min]	Type	Width [min]	Area [mAU*s]	Height [mAU]	Area %
1	5.823	MM R	0.0909	9290.31348	1703.03394	100.0000

Totals :   9290.31348 1703.03394

Signal 2: DAD1 B, Sig=254,4 Ref=off

Peak #	RetTime [min]	Type	Width [min]	Area [mAU*s]	Height [mAU]	Area %
1	5.823	MM R	0.0913	2231.48657	407.38834	100.0000

Totals :   2231.48657 407.38834

===
*** End of Report ***
